# Estimating inequality in alcohol-related liver disease burden in the UK, 2009 to 2020: a population-based study using routinely collected data

**DOI:** 10.1016/j.lanprc.2025.100002

**Published:** 2025-07

**Authors:** Zhaonan Wang, Krishnarajah Nirantharakumar, Arlene Copland, Darren Quelch, Rasiah Thayakaran, Joht Singh Chandan, James Ferguson, Matthew Brookes, Matthew Lewis, Neil Rajoriya, Nigel Trudgill, Ramesh Arasaradnam, Sally Bradberry, Shamil Haroon, Neeraj Bhala, Nicola J Adderley

**Affiliations:** a Department of Applied Health Sciences, University of Birmingham, Birmingham, UK; b National Institute for Health and Care Research Birmingham Biomedical Research Centre, Birmingham, UK; c Health Data Research UK (HDRUK), London, UK; d Sandwell and West Birmingham NHS Trust, Birmingham, UK; e Addictions Research Group, Applied Psychology Research and Innovation Group, University of South Wales, Pontypridd, Wales, UK; f Birmingham Health Partners, University of Birmingham, Birmingham, UK; g Liver Unit, Queen Elizabeth Hospital, Birmingham, UK; h College of Medicine and Health, University of Birmingham, Edgbaston, UK; i Faculty of Education, Health, and Wellbeing, Psychology Department, University of Wolverhampton, Wolverhampton, UK; j Royal Wolverhampton NHS Trust, Wolverhampton, UK; k University Hospital of North Midlands, Stoke-on-Trent, UK; l Department of Immunology and Immunotherapy, University of Birmingham, Birmingham, UK; m Department of Cancer and Genomic Sciences, University of Birmingham, Birmingham, UK; n Clinical Sciences Research Institute, University of Warwick, Coventry, UK; o Department of Gastroenterology, University Hospital Coventry and Warwickshire, Coventry, UK; p Medical School, University of Nottingham, Nottingham, UK; q Nottingham Digestive Diseases Centre, Translational Medical Sciences and NIHR Nottingham Biomedical Research Centre, Queens Medical Centre, Nottingham University Hospitals NHS Foundation Trust, Nottingham, UK

## Abstract

**Background:**

There is a need to understand the preventable burden of alcohol-related liver disease (ARLD) and to improve the identification of individuals at high risk. We aimed to establish reliable and stratified epidemiological data to understand the burden of ARLD and the inequalities in this burden related to ethnicity, socioeconomic factors, and region in the UK.

**Methods:**

Data were extracted from Clinical Practice Research Datalink Aurum, a primary care database that includes 20% of UK general practices. The study period was Jan 1, 2009, to Dec 31, 2020; all patients aged 18 years and older registered at a participating practice were eligible for inclusion. Hospital admission data were extracted from linked Hospital Episode Statistics (HES) and ARLD-specific mortality data were obtained from Office for National Statistics Death Registration Data. Several analytical approaches were used, as follows: yearly cross-sectional and cohort analyses to calculate the annual prevalence and incidence of ARLD, respectively; a retrospective, matched, open cohort study to assess all-cause mortality rates (in which patients without liver disease were matched with patients with ARLD on the basis of age, sex, ethnicity, and geographical region); and a retrospective, open cohort analysis to evaluate all-cause hospitalisation rates. Hospitalisation rates were calculated in those with ARLD only. We explored different definitions of ARLD, and our primary definition was definite ARLD (ie, a coded clinical record specifying ARLD). Incidence and prevalence were stratified by age, sex, ethnicity, deprivation (Index of Multiple Deprivation [IMD] quintile) and geographical region.

**Findings:**

During the study period, 19 534 887 patients from 1491 practices were eligible for inclusion in our study. For definite ARLD exposure, 257 544 patients were included in the all-cause mortality outcome analysis, of whom 51 510 were diagnosed with definite ARLD; while among the 50 409 patients with definite ARLD for whom HES-linked data were available, 37 142 had one or more hospital admissions. Prevalence of definite ARLD rose from 154 to 243 per 100 000 population from 2009 to 2020. Incidence increased from 18·6 to 30·3 per 100 000 person-years between 2009 and 2019, and then decreased to 24·7 per 100 000 person-years in 2020. Prevalence and incidence of ARLD by age, sex, ethnicity, geographical region, and IMD quintile increased between 2009 and 2020. The overall adjusted all-cause mortality hazard ratio (HR) for those with definite ARLD compared with no liver disease was 4·30 (95% CI 4·20–4·41). The effect of ARLD on mortality was more pronounced in younger than older age groups (eg, adjusted HR of 21·86 [95% CI 18·23–26·20]) in those aged 30–39 years *vs* 2·19 [2·09–2·29] in those ≥70 years) and in females than in males (5·61 [5·35–5·88] *vs* 3·93 [3·83–4·04]). The overall incidence rate for hospitalisations in patients with definite ARLD was 1·17 per person-year. Hospitalisation rates were higher in females (adjusted incidence rate ratio 1·03 [95% CI 1·01–1·06]) and in patients in more deprived groups (1·16 [1·10–1·21] in the most deprived IMD quintile *vs* the least deprived quintile).

**Interpretation:**

Our findings indicate an increasing burden of ARLD in the UK. Raising awareness of disparities in health outcomes in affected groups could facilitate earlier and more targeted interventions.

**Funding:**

National Institute for Health and Care Research Clinical Research Network West Midlands.

## Introduction

Excess alcohol intake is a primary modifiable risk factor associated with high burden of liver disease and cancer, in the UK and worldwide.^1^^,^^2^ Alcohol-related liver disease (ARLD) is one of the main gastrointestinal causes of mortality and morbidity throughout the UK.^3^ Alcohol-related deaths reached 14·0 per 100 000 people in 2020 in the UK, an 18·6% increase compared with 2019, representing the highest annual increase since annual statistical reporting began in 2001.^4^ Populations residing in urban communities have substantially higher mortality due to ARLD than the national average.^5^ Inequalities in this burden exist not only by geography and residential status, but also by social deprivation level and ethnicity.^6^^,^^7^ However, the burden of disease due to social deprivation and ethnicity is not reliably understood. Alongside policy initiatives (eg, price and availability of alcohol), improved methods of alcohol consumption screening in health-care settings are needed to identify individuals at high risk of ARLD.^8^^,^^9^ The effects of the combined impact of disproportionate alcohol-related harms through excessive alcohol consumption and increased clinical risk due to ARLD in groups with increased susceptibility, such as those experiencing socioeconomic disadvantage, are not well understood. As such, increased efforts through large-scale multifactorial studies to understand these inequalities are needed to: resolve uncertainties in preventing ARLD harms, tackle the preventable burden of ARLD, and improve identification of individuals at high risk of ARLD.

**Research in context**

**Evidence before this study**

We conducted a comprehensive literature search in PubMed using the following search terms to identify studies exploring the burden and health inequalities of alcohol-related liver disease (ARLD): (“alcohol-related liver disease” OR “alcohol related liver harm” OR “alcoholic liver disease”) AND (“burden” OR “inequality” OR “missed opportunity” OR “ethnicity” OR “region” OR “socioeconomic” OR “deprivation”) on April 1, 2022 and limited to studies published in English. Previous studies suggested that populations residing in urban communities have substantially higher mortality rates due to ARLD than the national average. Alcohol-related deaths, particularly those due to ARLD, are substantially more common in individuals older than 45 years. However, the burden of disease stratified by social deprivation and ethnicity is not reliably understood. Furthermore, in health-care settings, there is a need for improved methods of screening for alcohol consumption to identify individuals at high risk of ARLD.

**Added value of this study**

This study extends the existing body of previous research by using a large-scale primary care dataset from the UK to generate more reliable, comprehensive, and stratified epidemiological profiles of the incidence, prevalence, and mortality rates associated with ARLD. This study shows the increasing trends in prevalence and incidence of ARLD by age, sex, ethnicity, geographical region, and deprivation quintile between 2009 and 2020. High rates of all-cause mortality were observed among patients with ARLD, with younger adults, females, and those in the most deprived socioeconomic group disproportionately affected. Hospitalisations for those with ARLD were high, again particularly among the most deprived groups.

**Implications of all the available evidence**

The findings underscore the need for screening for and managing alcohol consumption in these groups. Awareness of the disproportionate effect of ARLD on younger people and females might encourage health-care practitioners to take earlier intervention steps within these populations.

The Clinical Practice Research Datalink (CPRD) Aurum is a primary care database that contains pseudonymised electronic health records from general practices across the UK, predominantly in England.^10^ CPRD Aurum includes data from almost 20% percent of the UK population.^11^ Due to its large sample size and broad demographic and geographical coverage, it is generalisable to the UK population and allows exploration of the inequalities and burden of ARLD in the UK.

Therefore, we aimed to establish more reliable, comprehensive, and stratified epidemiological data related to the incidence, prevalence, and mortality rates for ARLD. We focused on inequalities, with the aim of highlighting groups who might be at increased risk of ARLD-related morbidity and mortality, with a view to improving understanding of the burden of ARLD among these groups.

## Methods

### Study design, data sources, and study population

The population-based analyses included routinely collected patient data derived from the CPRD Aurum covering general practices across England and a small number in Northern Ireland. Patient demographic data such as sex and ethnicity, as well as diagnoses and all-cause mortality were obtained from CPRD Aurum.^10^ Linked Hospital Episode Statistics (HES) were used to obtain hospital admissions data; Office for National Statistics (ONS) death registration data were used to obtain ARLD-specific mortality; and linked Index of Multiple Deprivation (IMD) data were used to describe deprivation. The study period (prespecified in the study protocol) was Jan 1, 2009, to Dec 31, 2020.^12^ General practices opt in to the CPRD database; all patients at participating practices except patients who explicitly opt out of data collection are included in the database. HES data and death registrations are only available for practices in England. The Data Extraction for Epidemiological Research tool was used to extract data for the analysis.^13^ Further details on data sources and other aspects of the methods can be found in the appendix (pp 3–5).

We used the following analytical approaches: yearly cross-sectional analyses to calculate the annual prevalence of ARLD (analysis 1); yearly cohort analyses to calculate the annual incidence of ARLD (analysis 2); a retrospective, matched, open cohort analysis to assess all-cause mortality rates (analysis 3); and a retrospective, open cohort analysis to evaluate all-cause hospitalisation rates (analysis 4). In the cohort of patients with ARLD in analysis 4, we also evaluated ARLD-specific mortality and described consultations in primary care among people with ARLD in the periods up to 2 years and 3 years before diagnosis.

The source population for all studies was patients aged 18 years and older who were registered at a general practice contributing to CPRD Aurum and who did not opt out of data collection (eligible patients).

For the prevalence and incidence analyses (analyses 1 and 2), all patients aged 18 years and older who were registered with a participating practice during the study period were included in the denominator population (see appendix p 3).

For the mortality cohort analysis (analysis 3) and the hospital admissions cohort analysis (analysis 4), the index date for exposed patients (ie, those with ARLD) was the later of their ARLD diagnosis date (incident) or the date of being eligible (prevalent). Patients were included from their first ever diagnosis of ARLD recorded during the study period. Patients were followed up until the earliest event of death, individual leaving the practice, practice leaving the dataset, or study end (Dec 31, 2020).

In analysis 4, only patients with a recorded diagnosis of ARLD were included.

For all analyses, individuals with a recorded diagnosis of viral hepatitis or other specified non-ARLD were excluded.

Observational research using CPRD data was approved by the National Research Ethics Service Committee. This study was approved by the independent Research Data Governance Committee (reference 22-002131). The approved protocol is available online.^12^ Informed consent was not required for use of the pseudonymised patient data. A statement on patient and public involvement in the study design is in the appendix (pp 4–5).

### Definitions

A recorded diagnosis of ARLD was ascertained by the presence of a clinical (SNOMED CT) code using primary care records, using the following three definitions of ARLD: definite ARLD (clinically coded ARLD; appendix p 6); probable ARLD (clinically coded ARLD, or a clinical code for a non-specific liver disease [excluding liver disease due to other known causes such as viral hepatitis; appendix pp 7–9] in combination with a clinically coded record of alcohol misuse or excess alcohol consumption [>14 units per week]);^14^ or possible ARLD (clinically coded ARLD, or a clinical code for non-specific liver disease [excluding liver disease due to other known causes such as viral hepatitis; appendix p 10]).

The clinical code list for alcohol misuse included codes for consequences of alcohol misuse (appendix pp 11–15).

For our primary analyses, definite ARLD was used to define ARLD; other definitions (probable and possible) were used in secondary analyses. All three definitions were used for each of analyses 1–4. For analyses 1 and 2, prevalence and incidence of ARLD, using each of the three definitions, was calculated yearly by sociodemographic characteristics. For analysis 3, exposure was ARLD, and outcome was all-cause mortality (comparing participants with ARLD to those without liver disease). ARLD-related mortality outcome was also explored in those with ARLD only, with sociodemographic characteristics as exposures or risk factors. ICD-10 codes were used to identify the cause of deaths related to ARLD (appendix pp 16–17). For analysis 4, exposures and risk factors of interest were sociodemographic characteristics; outcome was any hospital admissions after index date. Sociodemographic characteristics were age, sex, region, ethnicity, and deprivation (IMD quintile).

Covariates in the study were age at index date, sex, geographical region, ethnicity, IMD quintile, smoking status, BMI, and presence of comorbidities at baseline, including cardiovascular disease, hypertension, diabetes (type 1 or type 2), chronic kidney disease (stage 3–5), cancer (any), chronic obstructive pulmonary disease, asthma, depression, anxiety, severe mental illness (including schizophrenia, bipolar disorder, psychosis, paranoid ideation, manic disorders, or delusional disorders), and dementia. Ethnicity was classified on the basis of UK census ethnic groups (White; Black, African, Caribbean, or Black British; South Asian; mixed or multiple ethnic groups; and other ethnic groups).

### Statistical analysis

This population-based study used all available data for the prespecified date range; therefore, we did not perform a sample size calculation.

We calculated yearly ARLD prevalence and annual incidence with 95% CIs at 1-year intervals from 2009 to 2020, with stratification of prevalence and incidence by age at index date (18–29, 30–39, 40–49, 50–59, 60–69, 70–79, and ≥80 years), sex (male and female), geographical region (North East, North West, Yorkshire and the Humber, East Midlands, West Midlands, East of England, South West, South East, and London), ethnicity (White; Black, African, Caribbean, or Black British; South Asian; mixed or multiple ethnic groups; and other ethnic groups), and IMD quintile (quintile 1 [least deprived] to quintile 5 [most deprived]). More details are in the appendix (p 4).

To analyse all-cause mortality, we did a retrospective, matched, open cohort analysis from 2009 to 2020 comparing all-cause mortality rates in adults with ARLD to adults without any liver disease. To mitigate the effect of important potential confounders, unexposed patients (without liver disease) were matched in a 1:4 ratio (without replacement) by age (±1 year), sex, ethnicity, and geographical region on the index date of the corresponding exposed patient.^13^^,^^15^ We used a Cox proportional hazards regression model to calculate crude and adjusted hazard ratios (HRs) with 95% CIs for mortality in the whole cohort and with stratification by age, sex, geographical region, ethnicity, and deprivation, with adjustment for all covariates listed above. More details are in the appendix (p 4).

In only patients with ARLD, we calculated HRs for ARLD-related mortality (using ONS mortality data) by age group, sex, geographical region, ethnicity, and deprivation, treating other causes of mortality as a competing risk.

To analyse hospital admission rates in patients with ARLD, we did a retrospective open cohort analysis from 2009 to 2020 comparing admission rates by age group, sex, geographical region, ethnicity, and IMD quintile. Stratifications were the same as in the mortality analyses. Hospitalisations (due to any cause) were captured using linked HES data. Because of overdispersion, we used a negative binomial regression model to calculate crude and adjusted incidence rate ratios (IRRs) with 95% CIs for admissions, with adjustment for the aforementioned covariates, allowing for multiple admissions per patient. More details are in the appendix (p 4).

Among patients with ARLD, we also described the number and proportion of primary care contacts in the periods up to 2 years and 3 years before ARLD diagnosis. For patients diagnosed early in the 2009–20 study period, primary care contacts up to 3 years before 2009 were captured. The frequency of consultations was classified as no contacts, 1, 2–5, 6–10, 11–20, 21–40, and more than 40.

All analyses were done using Stata 17.0.

### Role of the funding source

The funder of the study had no role in study design, data collection, data analysis, data interpretation, or writing of the report.

## Results

**Figure 1:**
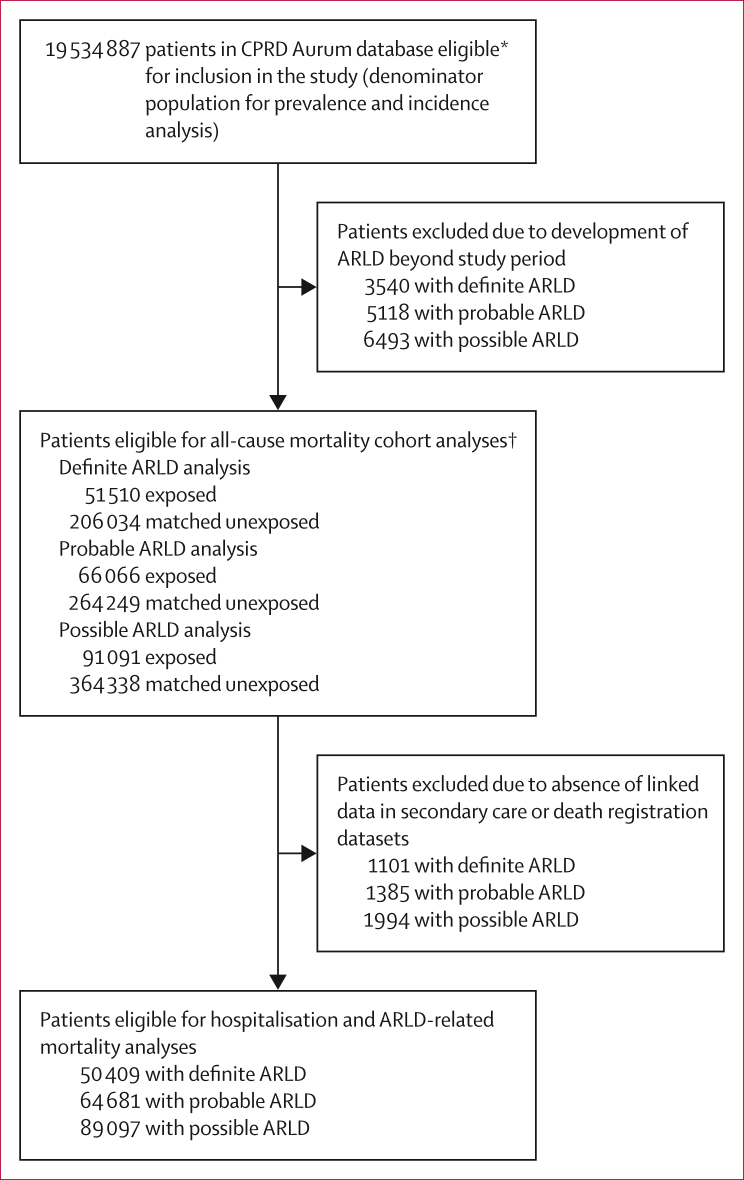
Flow chart of patient inclusion in each of the study analyses ARLD=alcohol-related liver disease. CPRD=Clinical Practice Research Datalink. ∗Eligible patients were those aged 18 years or older who were included in the database between 2009 and 2020. †Patients with ARLD (exposed) were matched in a 1:4 ratio to patients without any liver disease (unexposed), on the basis of age, sex, ethnicity, and geographical region.

From Jan 1, 2009, to Dec 31, 2020, there were 19 534 887 patients from 1491 practices in England and Northern Ireland in the CPRD Aurum database who were eligible for inclusion in our study. Numbers of patients included in each analysis are described in figure 1.

Over the study period, the prevalence of definite ARLD increased from 154 to 243 per 100 000 population, corresponding to an increase of 58%. An increase from 191 to 306 per 100 000 population (60%) was observed in the prevalence of probable ARLD. The prevalence of possible ARLD increased from 257 to 421 per 100 000 population (64%). Overall prevalence trends of ARLD are shown in the appendix (pp 18, 24–32).

**Figure 2:**
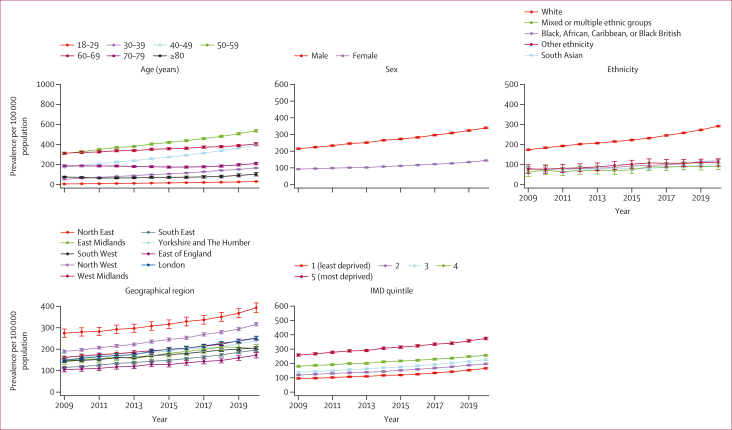
Annual prevalence trends of definite ARLD from 2009 to 2020, by age, sex, ethnicity, geographical region, and IMD quintile 95% CIs are present on all plots, but some are too small to be visible. Northern Ireland and the missing data category are omitted from the geographical region plot due to very small numbers of practices and patients (<100 prevalent patients in most years). Missing categories for ethnicity and IMD were not presented as they do not represent meaningful or interpretable population groups. The scales on the y axes differ between graphs. ARLD=alcohol-related liver disease. IMD=Index of Multiple Deprivation.

ARLD prevalence showed an increasing trend across all strata (figure 2). During the study period, people aged 50–59 years had the highest prevalence of definite ARLD, while those aged 18–29 years had the lowest prevalence. The prevalence in males was consistently more than twice that in females. The White population had a prevalence of definite ARLD that was 2–3 times higher than that of other ethnic groups and that increased from 174 to 292 per 100 000 population between 2009 and 2020. The prevalence of definite ARLD was higher in northern areas of England, with the North East having the highest rate, followed by the North West, while the East of England had the lowest rate. Prevalence in the most deprived group (IMD quintile 5) was more than twice that of the least deprived group (IMD quintile 1). The annual prevalence of definite ARLD is detailed in the appendix (pp 24–26). Similar trends were observed using probable and possible definitions of ARLD (appendix pp 20, 21, 27–32).

From 2009 to 2019, the incidence of definite ARLD increased from 18·6 to 30·3 per 100 000 person-years, corresponding to an increase of 63%, and then decreased to 24·7 per 100 000 person-years in 2020. Increasing incidence was also observed from 2009 to 2019 for probable ARLD (from 23·6 to 40·0 per 100 000 person-years, a 70% increase) and possible ARLD (from 33·6 to 58·6 per 100 000 person-years, a 74% increase), and a marked decrease in 2020 was also observed (to 35·0 and 52·8 per 100 000 person-years for probable and possible ARLD, respectively). These increases from 2019 to 2020 are probably secondary to reduced access to primary care during the first year of the COVID-19 pandemic (appendix pp 19, 33–41).

**Figure 3:**
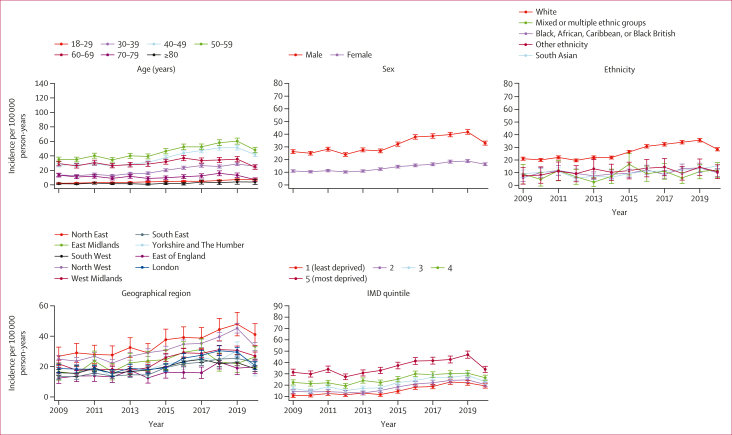
Annual incidence trends of definite ARLD from 2009 to 2020, by age, sex, ethnicity, geographical region, and IMD quintile 95% CIs are present on all plots, but some are too small to be visible. Northern Ireland and the missing data category are omitted from the region plot due to very small numbers of practices and patients (fewer than ten incident patients in most years). Missing categories for ethnicity and IMD were not presented as they do not represent meaningful or interpretable population groups. The scales on the y axes differ between graphs. ARLD=alcohol-related liver disease. IMD=Index of Multiple Deprivation.

Overall, the annual incidence of definite ARLD was highest in people aged 50–59 years, while those aged 80 years and older had the lowest rates (figure 3; appendix pp 33–35). Across the study period, the incidence of definite ARLD was more than twice as high in males as in females. The ethnicity-stratified incidence of definite ARLD was highest among the White population. At the regional level, the annual incidence was higher in northern areas of England, being highest in the North East. Higher levels of deprivation were associated with increasing incidence of ARLD. Similar incidence trends were observed for probable and possible ARLD (appendix pp 22, 23, 36–41).

For definite ARLD exposure, 51 510 patients diagnosed with definite ARLD (exposed) and 206 034 matched patients without liver disease (unexposed) were included in the all-cause mortality outcome analysis (table). Median follow-up was 3·0 years (IQR 1·2–6·4) in the exposed group and 4·4 years (1·9–8·6) in the unexposed group. Mean age was 54·9 years (SD 12·1) in both the exposed group and unexposed group. Most patients were male (36 176 [70·2%] of 51 510 exposed and 144 704 [70·2%] of 206 034 unexposed patients). Compared with matched patients without liver disease, those with definite ARLD were more likely to be in the most deprived IMD quintile (37 535 [18·2%] without liver disease *vs* 15 314 [29·7%] with definite ARLD), be current smokers (76 534 [37·1%] *vs* 33 044 [64·2%]), have a BMI of 30 kg/m^2^ or higher (45 585 [22·1%] *vs* 12 844 [24·9%]), and have more comorbidities (table). A similar distribution of baseline characteristics as in the definite ARLD cohort was observed for the probable and possible ARLD cohorts (table).

**Table:** Baseline characteristics of patients included in the all-cause mortality cohort analysis

	Definite ARLD analysis cohort		Probable ARLD analysis cohort		Possible ARLD analysis cohort	
	Definite ARLD (n=51 510)	Matched patients with no liver disease (n=206 034)	Probable ARLD (n=66 066)	Matched patients with no liver disease (n=264 249)	Possible ARLD (n=91 091)	Matched patients with no liver disease (n=364 338)
Age, years						
Mean (SD)	54·9 (12·1)	54·9 (12·1)	55·6 (12·3)	55·6 (12·4)	56·1 (14·1)	56·1 (14·1)
18–29	865 (1·7%)	3604 (1·7%)	1080 (1·6%)	4404 (1·7%)	3104 (3·4%)	12 484 (3·4%)
30–39	4788 (9·3%)	19 355 (9·4%)	5825 (8·8%)	23 491 (8·9%)	8683 (9·5%)	34 980 (9·6%)
40–49	12 389 (24·1%)	49 242 (23·9%)	15 218 (23·0%)	60 566 (22·9%)	18 912 (20·8%)	75 283 (20·7%)
50–59	15 802 (30·7%)	63 129 (30·6%)	19 880 (30·1%)	79 538 (30·1%)	24 443 (26·8%)	97 908 (26·9%)
60–69	12 019 (23·3%)	48 054 (23·3%)	15 721 (23·8%)	62 844 (23·8%)	20 574 (22·6%)	82 216 (22·6%)
≥70∗	5647 (11·0%)	22 650 (11·0%)	8342 (12·6%)	33 406 (12·6%)	15 375 (16·9%)	61 467 (16·9%)
Sex†						
Male	36 176 (70·2%)	144 704 (70·2%)	46 932 (71·0%)	187 719 (71·0%)	59 607 (65·4%)	238 413 (65·4%)
Female	15 333 (29·8%)	61 330 (29·8%)	19 133 (29·0%)	76 530 (29·0%)	31 483 (34·6%)	125 925 (34·6%)
Ethnicity						
White	39 565 (76·8%)	158 254 (76·8%)	50 982 (77·2%)	203 916 (77·2%)	66 262 (72·7%)	265 032 (72·7%)
Black, African, Caribbean, or Black British	860 (1·7%)	3440 (1·7%)	1116 (1·7%)	4461 (1·7%)	3001 (3·3%)	12 001 (3·3%)
South Asian	1604 (3·1%)	6416 (3·1%)	1998 (3·0%)	7992 (3·0%)	4900 (5·4%)	19 600 (5·4%)
Mixed or multiple	226 (0·4%)	904 (0·4%)	300 (0·5%)	1200 (0·5%)	660 (0·7%)	2637 (0·7%)
Other	305 (0·6%)	1220 (0·6%)	386 (0·6%)	1544 (0·6%)	739 (0·8%)	2952 (0·8%)
Missing data	8950 (17·4%)	35 800 (17·4%)	11 284 (17·1%)	45 136 (17·1%)	15 529 (17·0%)	62 116 (17·0%)
Geographical region						
North East	2589 (5·0%)	10 356 (5·0%)	3312 (5·0%)	13 248 (5·0%)	3899 (4·3%)	15 590 (4·3%)
North West	12 202 (23·7%)	48 808 (23·7%)	15 896 (24·1%)	63 584 (24·1%)	20 436 (22·4%)	81 744 (22·4%)
Yorkshire and The Humber	1804 (3·5%)	7216 (3·5%)	2302 (3·5%)	9208 (3·5%)	3096 (3·4%)	12 384 (3·4%)
East Midlands	1176 (2·3%)	4704 (2·3%)	1475 (2·2%)	5900 (2·2%)	2021 (2·2%)	8083 (2·2%)
West Midlands	8422 (16·4%)	33 688 (16·4%)	10 534 (15·9%)	42 136 (15·9%)	14 335 (15·7%)	57 340 (15·7%)
East of England	1586 (3·1%)	6344 (3·1%)	2069 (3·1%)	8276 (3·1%)	2970 (3·3%)	11 880 (3·3%)
South West	6280 (12·2%)	25 116 (12·2%)	8038 (12·2%)	32 148 (12·2%)	11 161 (12·3%)	44 640 (12·3%)
South East	8387 (16·3%)	33 548 (16·3%)	10 894 (16·5%)	43 576 (16·5%)	15 491 (17·0%)	61 964 (17·0%)
London	8833 (17·1%)	35 332 (17·1%)	11 234 (17·0%)	44 936 (17·0%)	17 214 (18·9%)	68 856 (18·9%)
Northern Ireland	202 (0·4%)	808 (0·4%)	277 (0·4%)	1105 (0·4%)	416 (0·5%)	1661 (0·5%)
Missing data	29 (0·1%)	114 (0·1%)	35 (0·1%)	132 (<0·1%)	52 (0·1%)	196 (0·1%)
IMD quintile						
1 (least deprived)	6617 (12·8%)	42 555 (20·7%)	8687 (13·1%)	54 188 (20·5%)	12 392 (13·6%)	73 157 (20·1%)
2	7932 (15·4%)	43 008 (20·9%)	10 300 (15·6%)	55 403 (21·0%)	14 524 (15·9%)	75 371 (20·7%)
3	9024 (17·5%)	39 347 (19·1%)	11 511 (17·4%)	50 371 (19·1%)	16 133 (17·7%)	69 451 (19·1%)
4	11 497 (22·3%)	38 710 (18·8%)	14 778 (22·4%)	50 158 (19·0%)	20 348 (22·3%)	70 817 (19·4%)
5 (most deprived)	15 314 (29·7%)	37 535 (18·2%)	19 380 (29·3%)	47 831 (18·1%)	25 710 (28·2%)	66 622 (18·3%)
Missing data	1126 (2·2%)	4879 (2·4%)	1410 (2·1%)	6298 (2·4%)	1984 (2·2%)	8920 (2·4%)
Smoking status						
Never smoked	7119 (13·8%)	63 386 (30·8%)	8907 (13·5%)	80 033 (30·3%)	16 859 (18·5%)	115 060 (31·6%)
Ex-smoker	10 426 (20·2%)	54 762 (26·6%)	14 170 (21·4%)	72 579 (27·5%)	22 596 (24·8%)	104 389 (28·7%)
Current smoker	33 044 (64·2%)	76 534 (37·1%)	41 867 (63·4%)	97 536 (36·9%)	49 758 (54·6%)	125 555 (34·5%)
Missing data	921 (1·8%)	11 352 (5·5%)	1122 (1·7%)	14 101 (5·3%)	1878 (2·1%)	19 334 (5·3%)
BMI, kg/m^2^						
<18·5	1895 (3·7%)	2280 (1·1%)	2341 (3·5%)	2866 (1·1%)	2951 (3·2%)	4896 (1·3%)
18·5 to <25	15 326 (29·8%)	57 588 (28·0%)	19 628 (29·7%)	73 366 (27·8%)	26 466 (29·1%)	104 426 (28·7%)
25 to <30	14 848 (28·8%)	66 046 (32·1%)	19 193 (29·1%)	85 619 (32·4%)	25 959 (28·5%)	116 750 (32·0%)
30 to <35	8425 (16·4%)	30 352 (14·7%)	10 975 (16·6%)	39 380 (14·9%)	15 221 (16·7%)	53 061 (14·6%)
35 to <40	3113 (6·0%)	10 335 (5·0%)	4115 (6·2%)	13 254 (5·0%)	6105 (6·7%)	17 846 (4·9%)
≥40	1306 (2·5%)	4898 (2·4%)	1811 (2·7%)	6638 (2·5%)	3040 (3·3%)	8628 (2·4%)
Missing data	6597 (12·8%)	34 535 (16·8%)	8003 (12·1%)	43 126 (16·3%)	11 349 (12·5%)	58 731 (16·1%)
Alcohol misuse	51 510 (100·0%)	10 182 (4·9%)	66 066 (100·0%)	64 197 (24·3%)	64 583 (70·9%)	78 931 (21·7%)
Excess alcohol consumption, units per week						
14–28	12 020 (23·3%)	31 215 (15·2%)	17 614 (26·7%)	40 893 (15·5%)	17 224 (18·9%)	50 550 (13·9%)
>28	19 256 (37·4%)	13 686 (6·6%)	24 406 (36·9%)	17 689 (6·7%)	24 148 (26·5%)	21 165 (5·8%)
Medical and psychiatric comorbidities						
Cardiovascular disease	7456 (14·5%)	18 303 (8·9%)	10 294 (15·6%)	25 018 (9·5%)	15 084 (16·6%)	37 218 (10·2%)
Hypertension	17 883 (34·7%)	47 060 (22·8%)	23 653 (35·8%)	63 244 (23·9%)	32 703 (35·9%)	92 062 (25·3%)
Type 1 diabetes	504 (1·0%)	1356 (0·7%)	746 (1·1%)	1788 (0·7%)	1273 (1·4%)	2385 (0·7%)
Type 2 diabetes	7403 (14·4%)	15 704 (7·6%)	10 679 (16·2%)	21 481 (8·1%)	17 548 (19·3%)	31 234 (8·6%)
Chronic kidney disease	1905 (3·7%)	7270 (3·5%)	2922 (4·4%)	10 302 (3·9%)	5915 (6·5%)	17 733 (4·9%)
Cancer	2428 (4·7%)	8972 (4·4%)	3625 (5·5%)	12 119 (4·6%)	6057 (6·6%)	18 020 (4·9%)
COPD	5024 (9·8%)	7428 (3·6%)	6554 (9·9%)	10 379 (3·9%)	8237 (9·0%)	14 443 (4·0%)
Asthma	9843 (19·1%)	28 848 (14·0%)	12 569 (19·0%)	37 431 (14·2%)	16 926 (18·6%)	51 974 (14·3%)
Dementia	868 (1·7%)	1157 (0·6%)	1130 (1·7%)	1642 (0·6%)	1663 (1·8%)	3445 (0·9%)
Anxiety	15 651 (30·4%)	27 996 (13·6%)	19 424 (29·4%)	35 934 (13·6%)	23 197 (25·5%)	48 405 (13·3%)
Depression	20 667 (40·1%)	37 403 (18·2%)	25 902 (39·2%)	47 753 (18·1%)	31 581 (34·7%)	64 027 (17·6%)
Severe mental illness‡	2172 (4·2%)	2753 (1·3%)	2846 (4·3%)	3571 (1·4%)	3310 (3·6%)	4817 (1·3%)

Data are n (%) except where otherwise specified. ARLD=alcohol-related liver disease. COPD=chronic obstructive pulmonary disease. IMD=Index of Multiple Deprivation.

∗ Patients aged 70–79 years and those aged ≥80 years were combined into a single ≥70 years age group in the mortality analysis due to the limited number of patients in these two groups.

† The number of patients with ARLD stratified by sex adds up to one less than the total as one patient was coded as intersex.

‡ Severe mental illness was defined as schizophrenia, bipolar disorder, psychosis, paranoid ideation, manic disorders, or delusional disorders.

**Figure 4:**
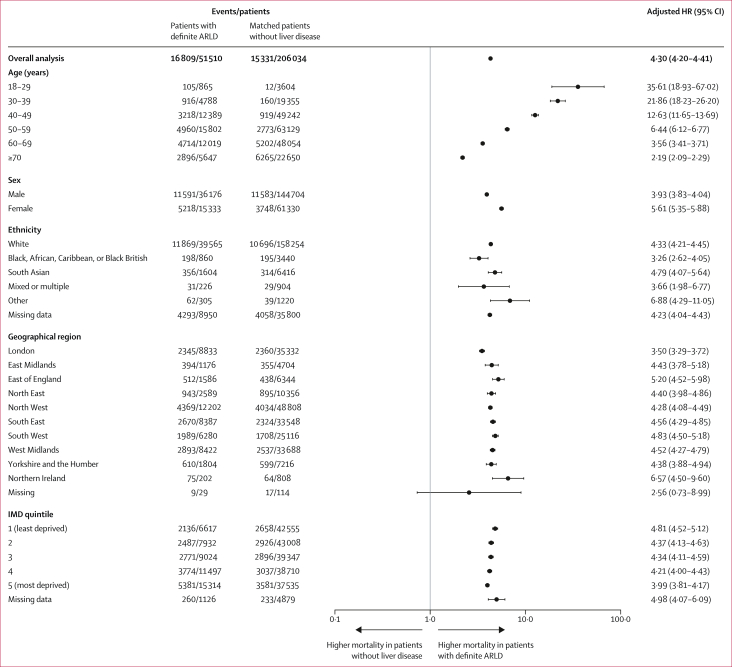
Adjusted HRs for all-cause mortality in patients with definite ARLD compared with those without liver disease, stratified by age, sex, ethnicity, geographical region, and IMD quintile Missing categories were included in the regression analyses to minimise selection bias; however, HRs for missing categories should not be interpreted because many have small numbers and it is not possible to know the characteristics of patients included in this group. ARLD=alcohol-related liver disease. HR=hazard ratio. IMD=Index of Multiple Deprivation.

In this analysis, 16 809 (6·5%) of 51 510 patients with definite ARLD and 15 331 (6·0%) of 206 034 matched patients without liver disease died (adjusted HR 4·30 [4·20–4·41]; figure 4; crude HRs are reported in the appendix [pp 42–43]). Across almost all stratified groups, the hazard of mortality was higher in the definite ARLD group than in the matched group without liver disease. The effect of definite ARLD on death in the younger age group (18–29 years) was much higher (adjusted HR 35·61 [95% CI 18·93–67·02]) than in the other age groups, although the absolute number of deaths in this age group was small (105 deaths in those with definite ARLD *vs* 12 in those without liver disease) and so the result should be interpreted with caution. The hazard of mortality was greater for females (adjusted HR 5·61 [95% CI 5·35–5·88]) than for males (3·93 [3·83–4·04]). By geographical region, London had an adjusted HR of 3·50 (95% CI 3·29–3·72), while all other geographical regions had adjusted HRs of 4·2 or higher, expect for those with missing regional data (figure 4). The increased hazard of death in patients with definite ARLD was similar in each deprivation quintile.

A similar hazard of all-cause mortality and similar trends in associations with sociodemographic factors were observed when comparing patients with probable or possible ARLD to matched patients without liver disease (appendix pp 42–45).

50 409 patients with definite ARLD had available cause-specific mortality and HES-linked data. Median follow-up for these patients was 2·9 years (IQR 1·2–6·4). Among these patients, there were 7618 ARLD-related deaths during the study period. Compared with the youngest age group (18–29 years), the adjusted hazard of ARLD-related death was higher in older age groups (appendix p 50; crude HRs available in the appendix [pp 48–49]). Compared with male patients, female patients with definite ARLD had an adjusted HR for ARLD-related mortality of 1·11 (95% CI 1·06–1·17]). Compared with London, patients with definite ARLD living in other regions had an increased hazard of ARLD-related death. Compared with the least deprived quintile, patients in the most deprived quintile had an adjusted HR for ARLD-related mortality of 1·16 (1·07–1·26), and there was no significant association for other deprivation quintiles. Crude and adjusted HRs for ARLD-related mortality among patients with probable and possible ARLD are shown in the appendix (pp 48–49, 51–52).

Among the 50 409 patients with definite ARLD for whom HES-linked data were available, 37 142 had one or more hospital admissions during the follow-up period. The overall incidence rate for hospitalisations was 1·17 per person-year. The mean age of the patients who were hospitalised was 55·6 years (SD 12·0), and most were male (25 648 [69·1%]), of White ethnicity (28 608 [77·0%]), current smokers (24 138 [65·0%]), and in the most deprived quintile (11 596 [31·2%], appendix pp 46–47).

Among patients with definite ARLD, hospitalisation rates were similar across age groups, but were significantly higher in patients aged 60–69 years than in the youngest age group in the adjusted analysis (appendix p 55; crude analyses are shown in the appendix pp 53–54). Hospitalisation rates were slightly higher in females than in males (adjusted IRR 1·03 [95% CI 1·01–1·06]). Patients with definite ARLD in all regions except Yorkshire and the Humber had a significantly higher rate of admissions than those in London. The most deprived IMD quintile was associated with increased hospitalisation rates compared with the least deprived quintile (eg, for quintile 5, adjusted IRR 1·16 [1·10–1·21]). The crude and adjusted IRRs for hospitalisations among patients with probable and possible ARLD are shown in the appendix (pp 53–54, 56–57).

The vast majority of patients subsequently diagnosed with definite ARLD (49 187 [95·5%] of 51 510) saw a general practitioner (GP) or a nurse at least once within 2 years before the diagnosis (appendix pp 58–59). Most visited the GP or nurse more than 11 times before their diagnosis (30 587 [59·4%] of 51 510). The median number of consultations was 14 among patients with definite (IQR 8–22), probable (8–23), and possible ARLD (8–24) within 2 years before diagnosis of ARLD.

Within 3 years before diagnosis of ARLD, the median number of consultations was 17 (IQR 9–29) among patients with definite ARLD, 18 (10–30) in those with probable ARLD, and 18 (9–31) in those with possible ARLD (appendix pp 60–61).

## Discussion

In this nationally representative observational study in the UK, we aimed to generate granular epidemiological data to understand the burden of ARLD and identify groups that might be at increased risk related to the disease. The population of patients affected by ARLD between 2009 and 2020 has grown. Individuals aged 50–59 years had the highest prevalence of ARLD. More specifically, males, those of White ethnicity, and the most deprived groups were found to have a higher prevalence of ARLD. Regionally, prevalence was higher in northern areas in England, with the North East having the highest rate of ARLD. Incident ARLD showed a similar increasing trend from 2009 to 2019, but dropped in 2020 most likely due to reduced access to health care during the COVID-19 pandemic. Associations between the incidence of ARLD and sociodemographic factors mirrored those observed for prevalence.

The impact of ARLD on risk of death was more pronounced in younger age groups than in older age groups and females than in males. Although individuals were less likely to develop ARLD at a younger age, the risk of all-cause death among patients with ARLD in this age group was comparatively higher than among older age groups. Among patients with ARLD, risk of ARLD-related mortality was higher among those aged 30 years and older than in the youngest age group (18–29 years). Fewer females than males developed ARLD; however, females with ARLD were more likely to die than their male counterparts (both all-cause and ARLD-related mortality).

Patients with ARLD in more deprived groups had higher rates of hospitalisation and death compared with those in the least deprived group. Almost all patients diagnosed with ARLD had visited their GP multiple times in the 2 years before their diagnosis, indicating potential opportunities for intervention.

There has been little research focusing on the prevalence and incidence of ARLD. To our knowledge, this is one of the first studies providing stratified annual prevalence and incidence estimates using a nationally representative database in the UK. A cohort study capturing ARLD cases from primary and secondary care datasets found an incidence of 56 per 100 000 person-years in 2018;^16^ this value is higher than the incidence of probable ARLD (which uses a similar definition to that used in the previous cohort study) of 37·7 per 100 000 person-years that we identified for 2018; however, it is very similar to the incidence of possible ARLD of 55·1 per 100 000 person-years. This finding is likely to reflect the fact that ARLD is often recorded in primary care data using non-specific liver disease codes, and this is the reason why we explored different methods of capturing ARLD using three alternative definitions. Our findings of high prevalence and incidence of ARLD in males and in northern areas of England extend existing evidence for high rates of ARLD in these groups indicated in national liver disease profiles.^17^^,^^18^ As in our study, these data indicate that groups with a higher level of deprivation have higher rates of hospitalisation for ARLD. The profiles have shown that hospital admission rates for ARLD were twice as high in males as in females in England, and that northern regions had the highest rate of admission for ARLD, with the lowest rate being in London.^17^^,^^18^ These profiles focused on hospital admissions specifically for ARLD, whereas we included any admissions, resulting in some differences. A large cross-sectional survey on the use and misuse of alcohol throughout the UK confirmed the widespread nature of binge-drinking (defined as feeling very drunk at least once a month) among young people (aged 18–24 years).^19^ Although smaller in absolute terms, we observed substantial relative effects of ARLD in our study in terms of elevated risks of death among young people with ARLD (HRs >20 in those aged ≤39 years) which have not been previously highlighted. The trend for disproportionate risk in women is also being seen in other areas of alcohol-related harms—for example, alcohol-related brain damage.^20^^,^^21^ Women might be more likely to exhibit signs of cognitive impairment following fewer years of harmful drinking behaviours than their male counterparts.^20^^,^^21^

The present study uses a population-based database which is representative of the UK population, with a long duration of follow-up that allows enough time for the development and diagnosis of the exposures and outcomes. In the mortality cohort study, patients were matched by age, sex, and ethnicity, and a range of potential confounders were adjusted for in the analysis. We did stratified analyses of prevalence and incidence and analysed mortality by sociodemographic characteristics, generating more granular data to identify potential inequalities and groups at increased risk. The analyses incorporated data from primary care and secondary care via linkage with HES to explore the impact of ARLD on both primary and secondary care use.

A limitation of the study is that there were some missing data for covariates including ethnicity, geographical region, IMD quintile, smoking status, alcohol consumption, and BMI; however, data were generally missing for only a small proportion of the total dataset; missing data were categorised as a separate group in the analysis, thereby minimising selection bias and maximising sample size. Alcohol consumption is poorly recorded in primary care and therefore this information was not available for many patients; additionally, recorded data might not always be accurate, particularly due to perceived stigma around reporting higher levels of alcohol consumption. Although patients with alcohol dependency might not interact with GPs, patient data from secondary care or emergency department visits are usually communicated back to primary care and recorded in the patient records; however, this might not always happen, and as a result there could be patients with undiagnosed ARLD in the comparator groups for the mortality analysis, which could lead to underestimated outcomes. ARLD might not be specifically coded in primary care data. To explore the effect of variation and accuracy of ARLD coding, we used three definitions of ARLD, ranging from very specific to more sensitive definitions, to maximise the capture of individuals with ARLD and to explore the impact of more or less sensitive definitions on the outcomes. The findings across all definitions were generally consistent, only differing in absolute number of ARLD cases. Linked secondary care data were not used to capture ARLD cases, which might have led to some ARLD cases not being identified. However, based on a comparison of incidence between our possible ARLD definition and a recent study that did use linked hospital data,^16^ the majority of cases were captured when using this more sensitive definition, although, in using this broad definition, there is a possibility of capturing patients without ARLD. In addition, it would be difficult to track changes in the type or severity of liver disease in this dataset due to the widespread use of generic liver disease codes in the database. Exclusion of viral hepatitis and other non-alcohol-related conditions might unintentionally exclude cases of liver disease related to alcohol use, potentially leading to an underestimation of burden. There were inevitably changes in the diagnosis and recording of ARLD during the COVID-19 pandemic period due to reduced health-care access, which affected the estimates of incidence in particular; however, trends up until this point were clear. Data after 2020 were not included in this analysis because at the time the prespecified study protocol was developed and approved, linked data were not available beyond this timepoint. The majority of practices contributing to CPRD Aurum are in England, with a small number in Northern Ireland;^11^ at present no practices in Wales or Scotland are captured, and it is therefore representative of the UK averages, but specific generalisation to all UK nations should be done with caution.

Our study adds important information on inequalities, both in terms of disease burden and outcomes, for the UK population with ARLD. In particular, age-related and sex-related effects were shown to be key factors, highlighting the important large relative risks of premature mortality in younger age groups and females, as well as higher relative hospital admission rates in females. Raising awareness of this disparity among clinicians and patients could facilitate earlier intervention in these groups. Socioeconomic and geographical characteristics were also notable in terms of their effects on outcomes. Higher rates of ARLD were observed in the most deprived IMD quintile, and it is likely that much of the regional difference is attributable to differences in socioeconomic deprivation, with higher prevalence and incidence observed in more socioeconomically deprived areas such as the North East of England.^22^ Consequently, it might be beneficial to target interventions for tackling alcohol misuse and ARLD in more deprived areas—for instance, interventions to change alcohol behaviours and reduce consumption (especially among heavy drinkers), and policy change with regard to the alcohol retail environment.

While the recorded incidence of ARLD decreased in 2020, most likely due to reduced health-care access during the COVID-19 pandemic, there is evidence of increased alcohol consumption during lockdowns among more deprived populations, in the northern regions of England, and among households that were usually the highest purchasers of alcohol before the pandemic.^23^ This increase in consumption might have led to increased ARLD-related morbidity and mortality in the post-pandemic period.^24^^,^^25^ Therefore, we anticipate that post-pandemic cases of ARLD have most likely returned to or exceeded their pre-pandemic levels; however, epidemiological studies using contemporary data are required to evaluate this.

Most studies to date have used either broad population health data to assess trends, such as alcohol drinking trends in surveys, or small secondary or tertiary liver transplant data to explore detailed phenotypes.^19^^,^^26^ Our study provides a UK-based perspective on the increasing burden of ARLD and sociodemographic variation in both burden and outcomes. Further work is needed to elucidate the reasons for these differences, but awareness of the disproportionate effect of ARLD on younger age groups and females might encourage health-care practitioners to intervene earlier in these groups. Prevention, early detection, and intervention in these at-risk groups should be prioritised. Furthermore, given our observation that patients with ARLD have frequent contact with their GP in the years leading up to diagnosis lends support to the importance of early detection. Stratifying groups at high risk, including inequalities related to protected characteristics and those driven by access to care and treatment, will continue to be important and needs more large-scale data evaluations.

## Data sharing

All data relevant to the study are included in the Article or uploaded as supplementary information in the appendix. Raw data from the study are not publicly available. Data for the study were obtained under licence from CPRD; pseudonymised patient data are available from CPRD subject to Research Data Governance approval; see https://www.cprd.com/how-access-cprd-data for more information.

## Declaration of interests

KN, NJA, and NB report funding from National Institute for Health and Care Research (NIHR) Clinical Research Network (CRN) West Midlands for this project. NJA reports grants from the NIHR and payment from Cegedim outside the submitted work. NB reports grants from Mayo Birmingham Exchange Fellowship supporting the present manuscript. KN reports grants from the NIHR, UK Research and Innovation (UKRI)/Medical Research Council, Kennedy Trust for Rheumatology Research, Health Data Research UK, Wellcome Trust, European Regional Development Fund, Institute for Global Innovation, Boehringer Ingelheim, Action Against Macular Degeneration Charity, Midlands Neuroscience Teaching and Development Funds, South Asian Health Foundation, Vifor Pharma, College of Police, and CSL Behring outside the submitted work; and receives consulting fees from BI, Sanofi, Cegedim, and MSD. KN holds leadership or fiduciary roles in the Network for Improving Critical Care Systems and Training (a charity) and OpenClinical (a social enterprise). DQ reports grants from Sandwell and West Birmingham NHS Trust Research Fellowship scheme supporting the present manuscript. JSC reports grants or contracts from the NIHR Youth Endowment Fund, College of Policing, University of Birmingham, and Birmingham City Council; and support for attending meetings from University of Miami and University of Washington. JF reports the following outside the submitted work: royalties from Wiley for a published book, consulting fees from Ipsen, payment for expert testimony from Hill Dickinson, support for attending meetings and/or travel from the European Association for the Study of the Liver and Ipsen, and being Chair of the British Liver Transplant Group. SH reports grants from the NIHR and UKRI outside the submitted work, and receives royalties from commercial licences issued for the Symptom Burden Questionnaire for Long COVID, and is a member of the Medicines and Healthcare Products Regulatory Agency CPRD Scientific Advisory Group. All other authors declare no competing interests.
