# Supplementary material for: Estimating inequality in alcohol-related liver disease burden in the UK, 2009 to 2020: a population-based study using routinely collected data
Source: Lancet Prim Care. 2025 Jul;1(1):None. doi: 10.1016/j.lanprc.2025.100002 (PMC12379629; doi:10.1016/j.lanprc.2025.100002)
Supplement: Supplementary appendix [file mmc1.pdf]

THE LANCET
Primary Care

Supplementary appendix

This appendix formed part of the original submission and has been peer reviewed. We post it as supplied by the authors.

Supplement to: Wang Z, Nirantharakumar K, Copland A, et al. Estimating inequality in alcohol-related liver disease burden in the UK, 2009 to 2020: a population-based study using routinely collected data. *The Lancet Primary Care* 2025. https://doi.org/10.1016/j.lanprc.2025.100002

Contents

Supplementary methods 3
Data source 3
Study population 3
Covariates 3
Statistical analysis 4
Patient and public involvement 4
Supplementary tables and figures 6
STable 1. Codes list for Definite ARLD 6
STable 2. Codes list for non-specific liver disease 7
STable 3. Codes list for non-alcohol-related liver disease (liver disease due to other known causes) 10
STable 4. Codes list for alcohol misuse 11
STable 5. ICD-10 codes for Definite ARLD 16
STable 6. ICD-10 codes for Probable ARLD 16
STable 7. ICD-10 codes for Possible ARLD 17
SFigure 1. Prevalence trends of ARLD from 2009 to 2020 18
SFigure 2. Annual incidence trends of ARLD from 2009 to 2020 19
SFigure 3. Annual prevalence trends of Probable ARLD from 2009 to 2020, by age, sex, ethnicity, region and IMD quintile 20
SFigure 4. Annual prevalence trends of Possible ARLD from 2009 to 2020, by age, sex, ethnicity, region and IMD quintile 21
SFigure 5. Annual incidence trends of Probable ARLD from 2009 to 2020, by age, sex, ethnicity, region and IMD quintile 22
SFigure 6. Annual incidence trends of Possible ARLD from 2009 to 2020, by age, sex, ethnicity, region and IMD quintile 23
STable 8. Annual Prevalence of Definite ARLD (per 100,000 population) 24
STable 9. Annual Prevalence of Probable ARLD (per 100,000 population) 27
STable 10. Annual Prevalence of Possible ARLD (per 100,000 population) 30
STable 11. Annual Incidence of Definite ARLD (per 100,000 person-years) 33
STable 12. Annual Incidence of Probable ARLD (per 100,000 person-years) 36
STable 13. Annual Incidence of Possible ARLD (per 100,000 person-years) 39
STable 14. Crude hazard ratios for all-cause mortality in people with ARLD compared to those without, stratified by age, sex, ethnicity, region and index of multiple deprivation (IMD) quintile 42
SFigure 7. Adjusted hazard ratios for all-cause mortality in people with Probable ARLD compared to those without liver disease, stratified by age, sex, ethnicity, region and IMD quintile 44
SFigure 8. Adjusted hazard ratios for all-cause mortality in people with Possible ARLD compared to those without liver disease, stratified by age, sex, ethnicity, region and IMD quintile 45
STable 15. Baseline characteristics of patients included in the ARLD-related mortality and hospitalisation cohort analysis 46
STable 16. Crude hazard ratios for ARLD-related mortality among people with ARLD, stratified by age, sex, ethnicity, region and index of multiple deprivation (IMD) quintile 48

SFigure 9. Adjusted hazard ratios for ARLD-related mortality among patients with Definite ARLD by age, sex, ethnicity, region and IMD quintile 50

SFigure 10. Adjusted hazard ratios for ARLD-related mortality among patients with Probable ARLD by age, sex, ethnicity, region and IMD quintile 51

SFigure 11. Adjusted hazard ratios for ARLD-related mortality among patients with Possible ARLD by age, sex, ethnicity, region and IMD quintile 52

STable 17. Crude incidence rate ratios for hospitalisations among patients with ARLD by sociodemographic characteristics 53

SFigure 12. Adjusted incidence rate ratios for hospitalisations among patients with Definite ARLD by age, sex, ethnicity, region and IMD quintile 55

SFigure 13. Adjusted incidence rate ratios for hospitalisations among patients with Probable ARLD by age, sex, ethnicity, region and IMD quintile 56

SFigure 14. Adjusted incidence rate ratios for hospitalisations among patients with Possible ARLD by age, sex, ethnicity, region and IMD quintile 57

SFigure 15. Consultations in primary care within 2 years before diagnosis among patients with ARLD 58

SFigure 16. Percentage of consultations in primary care within 2 years before diagnosis among patients with ARLD 59

SFigure 17. Consultations in primary care within 3 years before diagnosis among patients with ARLD 60

SFigure 18. Percentage of consultations in primary care within years before diagnosis among patients with ARLD 61

Supplementary methods

Data source

CPRD Aurum is a primary care database derived from electronic healthcare records across the UK.[1] It covers approximately 20% of the UK population[2] and has been demonstrated to be broadly representative of the UK population in terms of key demographic information including geographic spread, age and sex,[1,3] as well as ethnicity.[4] Data from patients in CPRD Aurum was linked to additional health-related data sources including small area-level data, Hospital Episode Statistics (HES) and Death Registration Data. Diagnoses, demographic characteristics, examinations, prescriptions and other health related information are recorded in the database.

Study population

Prevalence and annual incidence (Analyses 1 and 2): Denominator population was all individuals aged 18 years and above. Numerator was individuals with ARLD in the database on 1st January of each year (prevalence) or newly diagnosed within the relevant year (incidence). When calculating incidence, individuals with a previous diagnosis of ARLD prior to the start of the year were excluded.

Cohort studies (Analyses 3 and 4): Patients were eligible on the later of: the date of joining the dataset or reaching 18 years of age. Patients must have been registered with the GP practice for a minimum of 12 months to be eligible to participate, to ensure adequate recording of baseline information. An open cohort design was used: patients were able to enter and leave the cohort at any time during the study period; patients enter at the date they become eligible for the study, and leave at their exit date.

For the hospital admissions cohort study (Analysis 4), only patients with a record of ARLD were included. The index date for the exposed patients was the later of the date of ARLD diagnosis (incident patients) and the date the patient became eligible to join the study (the later of the date they joined the dataset or the date they reached 18 years of age; prevalent patients). Patients were followed up until the earliest of the following dates: death, individual left the practice, practice left the dataset, or study end date (31st December 2020).

Covariates

Ethnicity was defined based on UK census ethnic groups (White; Black, African, Caribbean, or Black British; South Asian; mixed or multiple ethnic groups; and other ethnic groups). IMD is a measure of relative deprivation based on small areas (postcode) and was classified as quintiles 1, least deprived, to 5, most deprived.[5] Severe mental illness included schizophrenia, bipolar disorder, psychosis, paranoid ideation, manic disorders or delusional disorders.

Statistical analysis

Prevalence and incidence of ARLD

Point prevalence was calculated using a series of cross-sectional analyses on 1st January in each year by dividing the total number of people with a record of ARLD on or before that date (numerator) by the total number of adults aged 18 or above in the dataset on that date (denominator). Individuals with a record of viral hepatitis and other specified non-alcoholic liver disease were excluded.

We calculated annual ARLD incidence with 95% CI over the period of one year from 1st January to 31st December each year between 2009 and 2020. Incidence was calculated using a series of annual cohort analyses, by dividing the number of newly diagnosed cases of ARLD within the given one-year period (numerator) by the number of person-years contributed by all eligible adults aged 18 and above within that year (denominator). Person-time was calculated by subtracting the index date from the exit date. Index date was the later of the following: 1st January in the given year, date the practice began contributing to CPRD Aurum, date the patient registered with the practice, and date the patient reached 18 years of age. Exit date was the earliest of: date the patient received a diagnosis of ARLD, date the patient was lost to follow-up (left the practice or practice stopped contributing to the dataset), patient died, or 31st December of the given year. Individuals with a record of viral hepatitis and other specified non-alcoholic liver disease on the index date were excluded.

Mortality cohort analysis

Individuals with a record of viral hepatitis and other specified non-alcoholic liver disease were excluded. We used descriptive statistics to summarize the characteristics of the patients. Continuous variables were summarized by mean (SD) or median (IQR) and categorical variables by number and percentage. The proportional hazards assumption was checked using the Schoenfelds residuals test and log-log plots.

Hospital admissions cohort analysis

A retrospective open cohort study from 2009 to 2020 was carried out comparing hospital admission rates (outcome) in adults with ARLD between age groups, males and females, regions, ethnic groups and IMD categories. Individuals with a record of viral hepatitis and other specified non-alcoholic liver disease were excluded.

Patient and public involvement

A meeting was held with a group of patients with experience of ARLD to discuss the findings of this study. Patients expressed the importance of addressing barriers to communication,

early identification of alcohol-related disorders, and improving links between mental health services and alcohol support services.

Supplementary tables and figures

STable 1. Codes list for Definite ARLD

Description	Read_code	SNOMED_CT_code
Esophageal varices in alcoholic cirrhosis of the liver		309783001
Alcoholic liver damage unspecified		589511000000102
Acute alcoholic hepatitis	J611.00	9953008
AH - Alcoholic hepatitis		235875008
Alcoholic cirrhosis of liver	J612.00	420054005
Alcoholic liver disease		41309000
ALD - Alcoholic liver disease		41309000
Alcoholic hepatic failure	J613000	235881000
Oesophageal varices in alcoholic cirrhosis of the liver	G852300	309783001
Chronic alcoholic hepatitis	J617000	307757001
Alcoholic liver damage unspecified	J613.00	41309000
Alcoholic cirrhosis		420054005
Alcoholic liver damage NOS	J613.99	589511000000102
Alcoholic steatosis		50325005
Alcoholic hepatitis	J617.00	235875008
Alcoholic fibrosis and sclerosis of liver	J612000	235880004
Alcoholic fatty liver	J610.00	50325005
Alcoholic liver cirrhosis		420054005
Acute alcoholic liver disease		9953008
Alcoholic fatty liver disease		50325005

STable 2. Codes list for non-specific liver disease

Description	Read_code	SNOMED_CT_code
Pigmentary portal cirrhosis	J615900	197299004
Oesophageal varices with haemorrhage		17709002
Esophageal varices associated with another disorder		195474004
HRF - Hepatorenal failure		51292008
CH - Chronic hepatitis		76783007
BOV - Bleeding esophageal varices		17709002
Cirrhosis secondary to cholestasis		271440004
Fiberoptic endoscopic injection sclerotherapy to esophageal varices		173639004
Esophageal varices without bleeding		14223005
Fiberoptic esophagoscopy and banding of esophageal varices		173641003
Esophageal varices with bleeding		17709002
Rigid esophagoscopy and injection sclerotherapy of varices		173660005
Laennec's cirrhosis		419728003
Bleeding esophageal varices		17709002
CAH - Chronic aggressive hepatitis		197284004
Esophageal varices without bleeding associated with another disorder		195476002
Pipestem portal cirrhosis		197300007
Ligation of esophageal varices		67364009
Bronze cirrhosis		399126000
Esophageal varices with bleeding associated with another disorder		195475003
Cerebrohepatorenal syndrome		88469006
Rigid esophagoscopy and banding of esophageal varices		173661009
Oesophageal varices NOS		675401000000103
Cirrhosis of liver due to cystic fibrosis		1763641000006109
Fibrocystic disease of liver		72925005
OV - Esophageal varices		28670008
Balloon tamponade of oesophageal varices		173679008
Cholestatic cirrhosis		1761006
Rigid esophagoscopic banding of esophageal varices		173661009
BOV - Bleeding oesophageal varices		17709002
Fiberoptic esophagoscopy and injection sclerotherapy of varices		173639004
Toxic portal cirrhosis	J615B00	197301006
Bronzed cirrhosis		399126000
Mixed micro AND macronodular cirrhosis		15999000
Esophageal varices with hemorrhage		17709002
Balloon tamponade of esophageal varices		173679008
Esophageal varices in cirrhosis of the liver		308129003
CLH - Chronic lobular hepatitis		57339008
Multilobar cirrhosis		43904005
Cholangitic cirrhosis		1761006
Open injection sclerotherapy to esophageal varices		173621008
Endoscopic injection sclerotherapy to varices of esophagus using rigid esophagoscope		173660005
Progressive neuronal degeneration with liver cirrhosis		20415001
Toxic liver disease with chronic lobular hepatitis	J635400	197360009
Syphilitic portal cirrhosis	J615F00	197305002
Pipe-stem portal cirrhosis	J615A00	197300007
CAH - Chronic active hepatitis		197284004
Open operations on esophageal varices		173617001
Oesophageal varices	G85..11	28670008

Chronic hepatitis NOS	J614z00	76783007
[X] Liver failure	J625.11	59927004
Fibrosis of liver		62484002
Bleeding oesophageal varices		17709002
Chronic hepatitis unspecified	J614y00	76783007
Hypertrophic portal cirrhosis	J615500	43904005
Oesophageal varices in diseases EC	G852.00	195474004
Chronic aggressive hepatitis	J614200	197284004
Portal cirrhosis unspecified	J615y00	419728003
CL - Cirrhosis of liver		19943007
Laennec's cirrhosis	J612.12	419728003
PHT - Portal hypertension		34742003
Oesophageal varices without bleeding	G851.00	14223005
[RFC] Cirrhosis		906081000006105
Reason for influenza vaccine - chronic liver disease		1850901000006104
Chronic persistent hepatitis	J614000	41889008
Other non-alcoholic chronic liver disease NOS	J61yz00	328383001
Portal cirrhosis	J615.11	419728003
Chronic lobular hepatitis	J614400	57339008
Infectious cirrhosis NOS	J615H00	235896001
Fibreoptic oesophagoscopy and injection sclerotherapy of varices		173639004
Postnecrotic cirrhosis of liver	J615111	43904005
CPH - Chronic persistent hepatitis		41889008
Oesophageal varices NOS	G858.00	28670008
Macronodular cirrhosis of liver	J615z11	43904005
Fibreoptic oesophagoscopy and banding of oesophageal varices		173641003
Cardiac portal cirrhosis	J615700	266470007
Oesophageal varices injected	7609499	173621008
Oesophageal varices in diseases EC NOS	G852z00	195474004
Mixed portal cirrhosis	J615200	15999000
Xanthomatous portal cirrhosis	J615C00	271440004
OV - Oesophageal varices		28670008
Chronic active hepatitis	J614100	197284004
Oesophageal varices with bleeding	G850.00	17709002
Fibreoptic endoscopic banding of oesophageal varices	760C500	173641003
Hepatic failure	J62y.13	59927004
Hepatic fibrosis	J615z15	62484002
Rigid oesophagoscopic banding of oesophageal varices	760F400	173661009
Toxic liver disease with fibrosis and cirrhosis of liver	J635600	197362001
Compensation for liver failure NOS	7L1fz00	773411000000107
Oesophageal varices	G858.99	675401000000103
Capsular portal cirrhosis	J615600	197296006
Other specified open operation on oesophageal varices	7609y00	173617001
Hepatic cirrhosis		19943007
Multilobular portal cirrhosis	J615100	266469006
Cystic fibrosis with liver manifestations		1854791000006100
Chronic yellow liver atrophy	J61y000	81675001
Bacterial portal cirrhosis	J615D00	197303009
Portal hypertension	J623.00	34742003
Chronic hepatitis	J614.00	76783007
Chronic liver disease NOS	J61z.00	328383001
Liver failure NOS	J62y.12	59927004
Fibrocystic liver disease	PB62100	72925005

Diffuse nodular cirrhosis	J615300	197293003
Hepatic failure as a complication of care	SP14200	213230009
Recurrent hepatitis	J614300	197286002
Hepatorenal syndrome as a complication of care	SP14300	213231008
Rigid oesophagoscopic injection sclerotherapy oesoph varices	760F300	173660005
Hepatic fibrosis with hepatic sclerosis	J61y600	235901004
Oesophageal varices without bleeding in diseases EC	G852100	195476002
Toxic liver disease with chronic persistent hepatitis	J635300	197359004
Oesophageal varices with bleeding associated with another disorder		195475003
Autoimmune chronic active hepatitis	J614111	197284004
Hepatic fibrosis	J61y400	62484002
Hepatorenal syndrome	J624.00	51292008
Other sequelae of chronic liver disease	J62y.00	235856003
Liver disease due to cystic fibrosis	C370700	427022004
Rigid oesophagoscopy and banding of oesophageal varices		173661009
Glycogenosis with hepatic cirrhosis	C310400	29633007
Macronodular cirrhosis		43904005
Open operation on oesophageal varices NOS	7609z00	173617001
Liver abscess and chronic liver disease causing sequelae NOS	J62z.00	197324004
Liver disease due to cystic fibrosis		1709341000006101
Congestive cirrhosis		74669004
Oesophageal varices injected	760F399	173660005
Fiberoptic endoscopic banding of esophageal varices		173641003
Unilobular portal cirrhosis	J615000	197291001
Cirrhosis of liver NOS	J615z13	19943007
Cirrhosis and chronic liver disease	J61..00	197279005
Oesophageal varices in cirrhosis of the liver	G852200	308129003
[X] Hepatic failure	J625.00	59927004
Liver abscess and sequelae of chronic liver disease	J62..00	197324004
[X]Oesophageal varices in diseases classified elsewhere	Gyu9400	28670008
Compensation for liver failure	7L1f.00	773411000000107
Portal fibrosis without cirrhosis	J61y300	197316009
Chronic hepatitis annual review - enhanced services admin	9kR..00	362421000000102
Chronic hepatitis annual review	9kR..11	362421000000102
Fatty portal cirrhosis	J615400	197294009
Pigmentary cirrhosis of liver	C350012	399126000
Florid cirrhosis	J612.11	76301009
Congestive cirrhosis	J615711	74669004
Oesophageal varices associated with another disorder		195474004
Esophageal varices		28670008
Hepatorenal failure		51292008
Cryptogenic cirrhosis of liver	J615z12	89580002
[X]Other and unspecified cirrhosis of liver	Jyu7100	19943007
Open injection sclerotherapy to oesophageal varices	7609400	173621008
Local ligation of oesophageal varices	7609300	67364009
Liver failure as a complication of care	SP14211	213230009
Open operations on oesophageal varices	7609.00	173617001
Hepatic sclerosis	J61y500	235899008
Oesophageal varices with bleeding in diseases EC	G852000	195475003
Toxic liver disease with chronic active hepatitis	J635500	197361008
Rigid oesophagoscopy and injection sclerotherapy of varices		173660005
PC - Portal cirrhosis		419728003
Oesophageal varices without bleeding associated with another disorder		195476002

STable 3. Codes list for non-alcohol-related liver disease (liver disease due to other known causes)

Description	Read_code	SNOMED_CT_code
ICC - Indian childhood cirrhosis		6183001
Hanot's cirrhosis		31712002
Laennec cirrhosis		235895002
Cirrhosis nonalcoholic		266468003
Chronic viral hepatitis B without delta-agent	A707100	186639003
Biliary cirrhosis NOS	J616z00	1761006
Other non-alcoholic chronic liver disease NOS	J61yz00	328383001
Laennec's cirrhosis non-alcoholic	J615z14	235895002
Biliary cirrhosis	J616.00	1761006
Chronic viral hepatitis	A707.00	10295004
Other non-alcoholic chronic liver disease	J61y.00	79720007
Secondary biliary cirrhosis	J616100	12368000
[X]Other chronic viral hepatitis	AyuB100	10295004
Chronic type C viral hepatitis		128302006
Non-alcoholic cirrhosis NOS	J615z00	266468003
Primary biliary cirrhosis	J616000	31712002
Chronic viral hepatitis C	A707200	128302006
Chronic type B viral hepatitis		61977001
Juvenile portal cirrhosis	J615800	266471006
Cirrhosis of liver not due to alcohol		266468003
Congenital hepatic fibrosis	Q48yz11	79607001
Childhood function cirrhosis	J615811	197284004
Indian childhood cirrhosis	J615812	6183001
Chronic viral hepatitis B with delta-agent	A707000	235869004
[X]Chronic viral hepatitis unspecified	AyuB200	10295004
Chronic viral hepatitis B	A707300	61977001
Cirrhosis - non alcoholic	J615.00	266468003
Chronic viral hepatitis B with hepatitis D		235869004
Chronic viral hepatitis unspecified	A707X00	10295004
Biliary cirrhosis of children	J616200	197310003

STable 4. Codes list for alcohol misuse

Description	Read_code	SNOMED_CT_code
Dental recall - excessive alcohol use		1662551000006109
Alcohol dependence resolved		1894171000006107
Mental & behav dis due to use alcohol: psychotic disorder predominantly polymorphic		1975341000006103
Mental & behav dis due to use alcohol: resid & late-onset psychot dis, flashbacks		1975511000006108
Mental & behav dis due to use alcohol: psychotic disorder, predominantly manic symptoms		1975411000006107
Dilated cardiomyopathy caused by alcohol		83521008
Alcoholic myositis		19303008
Alcohol induced encephalopathy		192811002
Alcoholic myopathic syndrome		19303008
Pseudo-Cushing syndrome due to alcohol		237738005
Alcoholic psychoses		42344001
FAS - Foetal alcohol syndrome		205788004
Esophageal varices in alcoholic cirrhosis of the liver		309783001
Other alcoholic psychosis		623961000000108
Alcoholic liver damage unspecified		589511000000102
Alcohol-related legal or disciplinary problem		1808491000006109
Alcohol-induced psychotic disorder with hallucinations		7052005
Alcohol induced hallucinosis		7052005
Alcoholic psychosis		42344001
Korsakov alcoholic psychosis with peripheral neuritis		191471000
Mental & behav dis due to use alcohol: acute intoxication with convulsions		1973441000006105
Mental & behav dis due to use alcohol: psychotic disorder schizophrenia-like		1975141000006101
Alcohol-induced pseudo-Cushing syndrome		237738005
Alcohol-induced persisting amnestic disorder		73097000
Brief intervention for excessive alcohol consumptn completed	9k1A.00	366371000000105
Alcohol problem drinking	E23..12	7200002
[X]Acute alcoholic drunkenness	Eu10011	25702006
Alcoholism		7200002
Excessive use of alcohol		961571000006107
Acute alcoholic hepatitis	J611.00	9953008
Chronic alcoholism NOS	E231z00	66590003
Alcoholic polyneuropathy	F375.00	7916009
Nondependent alcohol abuse episodic	E250200	191883007
Other alcoholic psychosis	E01y.00	42344001
[X]Mental & behav dis due to use alcohol: acute intoxication	Eu10000	25702006
[V]Problems related to lifestyle alcohol use	ZV11311	371422002
Delivery of rehabilitation for alcohol addiction	7P22100	231161000000109
Alcoholic peripheral neuropathy		7916009
[X]Mental and behav dis due to use alcohol: withdrawal state	Eu10300	191480000
Alcoholic paranoia	E015.00	191478006
Korsakov's alcoholic psychosis with peripheral neuritis	E011100	191471000
[X]Men & behav dis due alcohl: withdrawl state with delirium	Eu10400	8635005

[X]Alcoholic paranoia	Eu10513	42344001
Dipsomania	E231.11	7200002
Amnesic syndrome due to alcohol		69482004
Mental & behav dis due to use alcohol: psychotic disorder predominantly hallucinatory		1975291000006101
Mental & behav dis due to use alcohol: withdrawl state with delirium without convulsions		1974301000006101
History of alcoholism		161466001
Korsakov syndrome - alcoholic		69482004
AH - Alcoholic hepatitis		235875008
Acute alcoholism		25702006
Alcohol dependence syndrome	E23..00	66590003
Unspecified chronic alcoholism	E231000	66590003
Alcoholic cirrhosis of liver	J612.00	420054005
Chronic alcoholism	E231.00	66590003
[X]Alcohol addiction	Eu10211	66590003
Alcoholic dementia NOS	E012.11	281004
Non-dependent abuse of alcohol	E250.99	268645007
Episodic chronic alcoholism	E231200	191812006
Detoxication psychiatric therapy for alcoholism		64297001
Wernicke-Korsakov syndrome	E011200	69482004
Alcohol-induced epilepsy	F25B.00	361268000
Chronic alcoholic brain syndrome	E012000	191475009
[X]Delirium, not induced by alcohol+other psychoactive subs	Eu04.00	111479008
Alcoholic liver disease		41309000
ALD - Alcoholic liver disease		41309000
Mental & behav dis due to use alcohol: acute intoxication with trauma or other bodily injury		1972161000006108
Detoxication therapy for alcoholism		64297001
Mental & behav dis due to use alcohol: psychotic disorder, predominantly delusional		1975181000006107
Alcohol dependence syndrome NOS	E23z.00	66590003
Nondependent alcohol abuse, unspecified	E250000	268645007
[X]Mental and behavioural disorders due to use of alcohol	Eu10.00	29212009
Acute alcoholic intoxication in alcoholism	E230.00	191802004
Continuous chronic alcoholism	E231100	191811004
Alcoholic psychoses	E01..00	42344001
Alcohol withdrawal delirium	E010.00	8635005
Alcoholic hepatic failure	J613000	235881000
Oesophageal varices in alcoholic cirrhosis of the liver	G852300	309783001
[X]Alcoholic psychosis NOS	Eu10514	42344001
Evidence of alcohol withdrawal		940781000006100
Aversion therapy - alcoholism	8G32.00	183388004
Chronic alcoholic hepatitis	J617000	307757001
Cerebral degeneration due to alcoholism	F11x000	192811002
Mental and behav dis due to use alcohol: dependence syndr, continuous use		1974111000006101
Alcohol amnestic syndrome	E011.00	69482004
Alcohol dependence resolved	2126C00	918321000000104
Mental & behav dis due to use alcohol: acute intoxication, uncomplicated		1972151000006106
Foetal alcohol syndrome		205788004

Mental & behav dis due to use alcohol: withdrawl state with delirium, with convulsions		1974521000006101
Mental & behav dis due to use alcohol: acute intoxication with delirium		1973241000006109
Alcohol-related disorder		29212009
Mental & behav dis due to use alcohol: resid & late-onset psychot dis, residual affective disorder		1975661000006107
Nondependent alcohol abuse	E250.00	268645007
Hazardous alcohol use	136S.00	198421000000108
Alcoholic liver damage unspecified	J613.00	41309000
Extended intervention for excessive alcohol consumptn complt	9k1B.00	366421000000103
[X]Alcohol withdrawal-induced seizure	Eu10800	308742005
Alcoholic cirrhosis		420054005
Korsakov's alcoholic psychosis	E011000	69482004
[X]Chronic alcoholism	Eu10212	66590003
[X]Korsakov's psychosis, alcohol induced	Eu10611	69482004
Nondependent alcohol abuse NOS	E250z00	268645007
Dementia associated with alcoholism		281004
Alcoholic liver damage NOS	J613.99	589511000000102
Other alcoholic dementia	E012.00	281004
Alcohol withdrawal hallucinosis	E013.00	191476005
Episodic acute alcoholic intoxication in alcoholism	E230200	191805002
Alcoholic steatosis		50325005
Alcohol dependence with acute alcoholic intoxication	E230.11	191802004
Dilated cardiomyopathy secondary to alcohol		83521008
Mental & behav dis due to use alcohol: resid & late-onset psychot dis, other persisting cognitive impairment		1976071000006103
Mental and behav dis due to use alcohol: withdrawal state, with convulsions		1974231000006101
Mental & behav dis due to use alcohol: psychotic disorder, predominantly depressive symptoms		1975371000006106
Mental & behav dis due to use alcohol: acute intoxication, with other medical complications		1972271000006108
Korsakov psychosis		69482004
Alcohol abuse prevention education		408947007
Alcoholic cardiomyopathy	G555.00	83521008
Alcohol-induced chronic pancreatitis	J671000	235952002
Alcohol withdrawal-induced convulsion		308742005
Alcohol-induced acute pancreatitis	J670800	235942001
[V]Personal history of alcoholism	ZV11300	371422002
[X]Alcoholic dementia NOS	Eu10711	281004
Excessive alcohol consumption		160592001
[X]Alcoholic hallucinosis	Eu10511	7052005
Disqualified from driving due to excess alcohol	13ZY.00	279651000000104
[X]Mental & behav dis due to use alcohol: psychotic disorder	Eu10500	42344001
Acute alcoholic intoxication, unspecified, in alcoholism	E230000	191802004
Acute alcoholic intoxication in alcoholism NOS	E230z00	191802004
Mental & behav dis due to use alcohol: psychotic disorder, mixed		1975461000006105

Mental and behav dis due to use alcohol: withdrawal state, uncomplicated		1974191000006106
[X]Alcoholic jealousy	Eu10512	42344001
XS - Excessive alcohol consumption		160592001
Extended intervention for excessive alcohol consumption declined		1745961000006107
Mental and behav dis due to use alcohol: dependence syndr, currently on a clinically supervised maintenance or replacement regime [controlled dependence]		1973871000006100
[X]Dipsomania	Eu10213	66590003
Alcohol-induced persisting dementia		281004
AA - Alcohol abuse		15167005
Brief intervention for excessive alcohol consumptn declined	8IAF.00	379411000000105
Alcoholic hepatitis	J617.00	235875008
Alcoholic encephalopathy	F11x011	192811002
Alcoholic fibrosis and sclerosis of liver	J612000	235880004
Alcohol induced hallucinations	1B1c.00	417633001
Alcoholic myopathy	F394100	19303008
Nondependent alcohol abuse in remission	E250300	191884001
[X]Men & behav dis due alcoh: resid & late-onset psychot dis	Eu10700	42344001
Alcoholic polyneuropathy		7916009
Mental & behav dis due to use alcohol: resid & late-onset psychot dis, personality or behaviour disorder		1975541000006107
Alcoholic amnestic syndrome		69482004
Other alcoholic psychosis NOS	E01yz00	42344001
Mental & behav dis due to use alcohol: acute intoxication, pathological intoxication		1973481000006104
Alcoholism	E23..11	66590003
Alcoholic fatty liver	J610.00	50325005
Alcoholic gastritis	J153.00	2043009
[X]Mental and behav dis due to use of alcohol: harmful use	Eu10100	15167005
Nondependent alcohol abuse, continuous	E250100	191882002
[X]Delirium tremens, alcohol induced	Eu10411	8635005
Uncomplicated alcohol withdrawal		85561006
[X]Ment & behav dis due use alcohol: unsp ment & behav dis	Eu10z00	91388009
Alcoholic psychosis NOS	E01z.00	42344001
[X]Mental and behav dis due to use alcohol: amnesic syndrome	Eu10600	73097000
Pathological alcohol intoxication	E014.00	191477001
[X]Men & behav dis due to use alcohol: oth men & behav dis	Eu10y00	29212009
Acute alcoholic intoxication in remission, in alcoholism	E230300	191806001
Mental and behav dis due to use alcohol: dependence syndr, currently abstinent		1973701000006103
Continuous acute alcoholic intoxication in alcoholism	E230100	191804003
Alcohol-induced pseudo-Cushing's syndrome	C150500	237738005
Alcoholic liver cirrhosis		420054005

Mental and behav dis due to use alcohol: dependence syndr, currently using the substance [active dependence]		1974071000006109
Mental and behav dis due to use alcohol: dependence syndr, currently abstinent, but receiving treatment with aversive or blocking drugs		1974021000006108
Mental & behav dis due to use alcohol: resid & late-onset psychot dis, dementia		1975591000006103
Alcohol induced psychosis		42344001
Alcohol withdrawal syndrome	E01y000	191480000
Harmful alcohol use	136T.00	198431000000105
H/O: alcoholism	1462.00	161466001
Cirrhosis - non alcoholic	J615.00	266468003
[X]Mental and behav dis due to use alcohol: dependence syndr	Eu10200	66590003
Extended interven for excessive alcohol consumption declined	8IAt.00	754831000000102
Chronic alcoholism in remission	E231300	191813001
Cerebellar ataxia due to alcoholism	F144000	361272001
Excessive alcohol use		160592001
[X]Chronic alcoholic brain syndrome	Eu10712	191475009
Other alcoholic psychoses	E01y.99	623961000000108
Alcohol amnestic syndrome NOS	E011z00	73097000
Acute alcoholic liver disease		9953008
Alcoholic fatty liver disease		50325005
Mental and behav dis due to use alcohol: dependence syndr, episodic use [dipsomania]		1974141000006102
Mental and behav dis due to use alcohol: dependence syndr, currently abstinent, but in a protected environment		1973811000006109
Mental & behav dis due to use alcohol: resid & late-onset psychot dis, late-onset psychotic disorder		1976111000006106
Mental & behav dis due to use alcohol: acute intoxication, with coma		1973411000006106
Mental & behav dis due to use alcohol: acute intoxication, with perceptual distortions		1973321000006109

STable 5. ICD-10 codes for Definite ARLD

Medical code ID	Description
K70	Alcoholic liver disease
K700	Alcoholic fatty liver
K701	Alcoholic hepatitis
K702	Alcoholic fibrosis and sclerosis of liver
K703	Alcoholic cirrhosis of liver
K704	Alcoholic hepatic failure
K709	Alcoholic liver disease, unspecified

STable 6. ICD-10 codes for Probable ARLD

Medical code ID	Description
K70	Alcoholic liver disease
K700	Alcoholic fatty liver
K701	Alcoholic hepatitis
K702	Alcoholic fibrosis and sclerosis of liver
K703	Alcoholic cirrhosis of liver
K704	Alcoholic hepatic failure
K709	Alcoholic liver disease, unspecified
K74	Fibrosis and cirrhosis of liver
K740	Hepatic fibrosis
K741	Hepatic sclerosis
K742	Hepatic fibrosis with hepatic sclerosis
K746	Other and unspecified cirrhosis of liver

STable 7. ICD-10 codes for Possible ARLD

Medical code ID	Description
K70	Alcoholic liver disease
K700	Alcoholic fatty liver
K701	Alcoholic hepatitis
K702	Alcoholic fibrosis and sclerosis of liver
K703	Alcoholic cirrhosis of liver
K704	Alcoholic hepatic failure
K709	Alcoholic liver disease, unspecified
K74	Fibrosis and cirrhosis of liver
K740	Hepatic fibrosis
K741	Hepatic sclerosis
K742	Hepatic fibrosis with hepatic sclerosis
K746	Other and unspecified cirrhosis of liver
K75	Other inflammatory liver diseases
K750	Abscess of liver
K751	Phlebitis of portal vein
K752	Nonspecific reactive hepatitis
K753	Granulomatous hepatitis, not elsewhere classified
K754	Autoimmune hepatitis
K758	Other specified inflammatory liver diseases
K759	Inflammatory liver disease, unspecified
K76	Other diseases of liver
K760	Fatty (change of) liver, not elsewhere classified
K761	Chronic passive congestion of liver
K762	Central haemorrhagic necrosis of liver
K763	Infarction of liver
K764	Peliosis hepatis
K765	Hepatic veno-occlusive disease
K766	Portal hypertension
K767	Hepatorenal syndrome
K768	Other specified diseases of liver
K769	Liver disease, unspecified
K77	Liver disorders in diseases classified elsewhere
K770	Liver disorders in infectious and parasitic diseases classified elsewhere
K778	Liver disorders in other diseases classified elsewhere

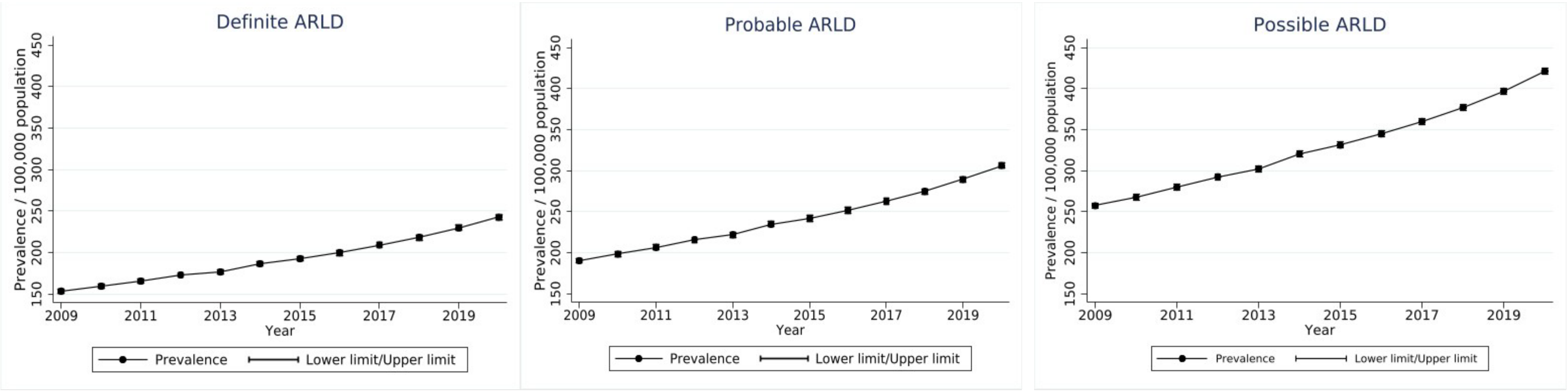

SFigure 1. Prevalence trends of ARLD from 2009 to 2020
ARLD = alcohol related liver disease. (95% confidence intervals are drawn but are too small to be visible.)

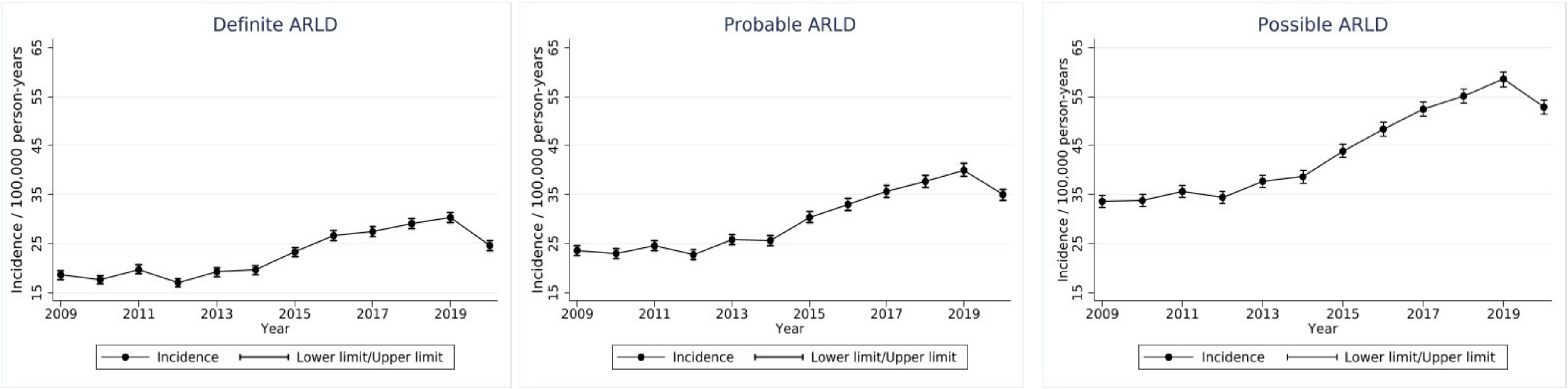

SFigure 2. Annual incidence trends of ARLD from 2009 to 2020

ARLD = alcohol related liver disease. (95% confidence intervals are drawn but are too small to be visible.)

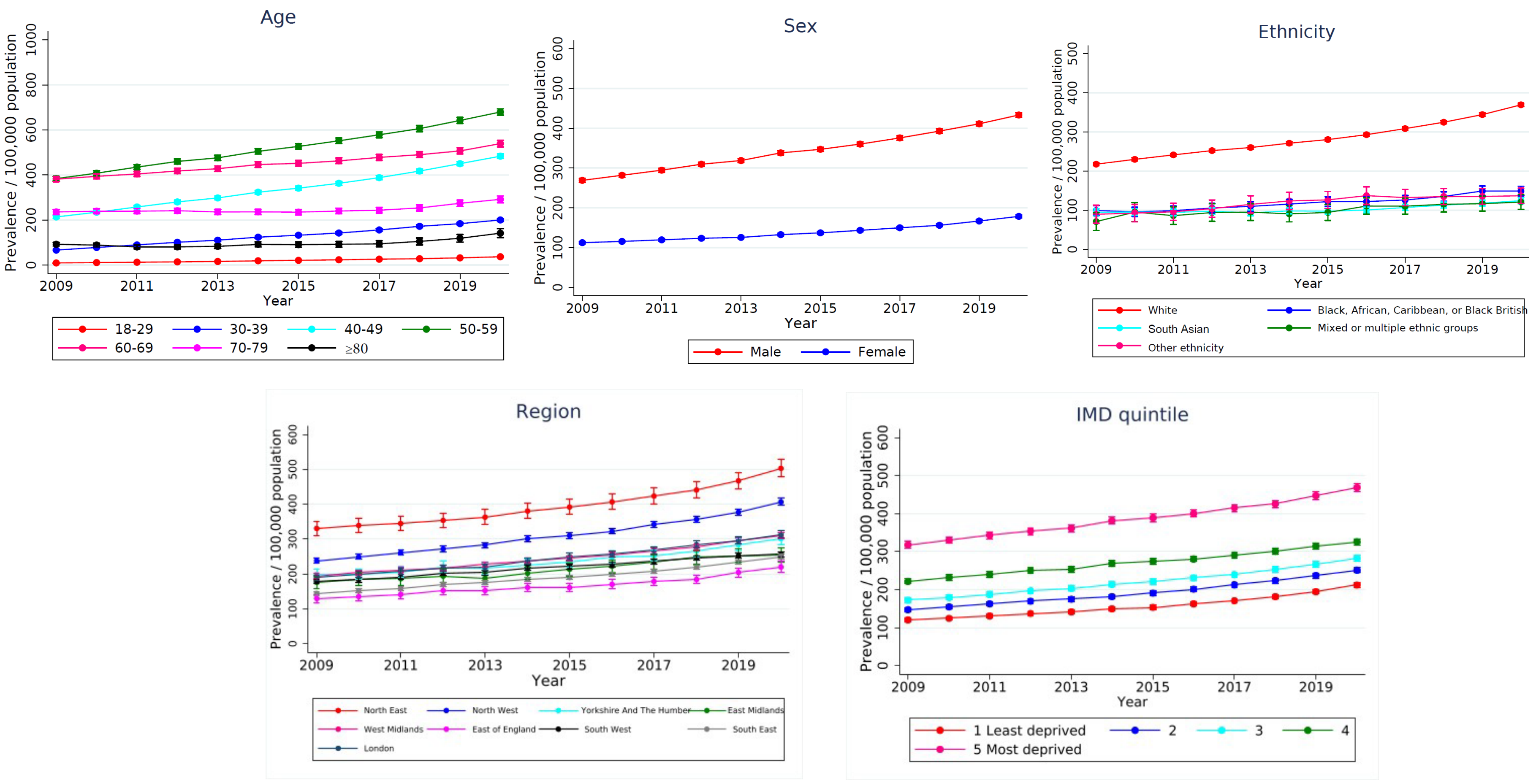

SFigure 3. Annual prevalence trends of Probable ARLD from 2009 to 2020, by age, sex, ethnicity, region and IMD quintile

ARLD = alcohol related liver disease; IMD = Index of Multiple Deprivation. (95% confidence intervals are drawn but are too small to be visible.) Northern Ireland and missing region are omitted from the region plot due to very small numbers of practices and patients.

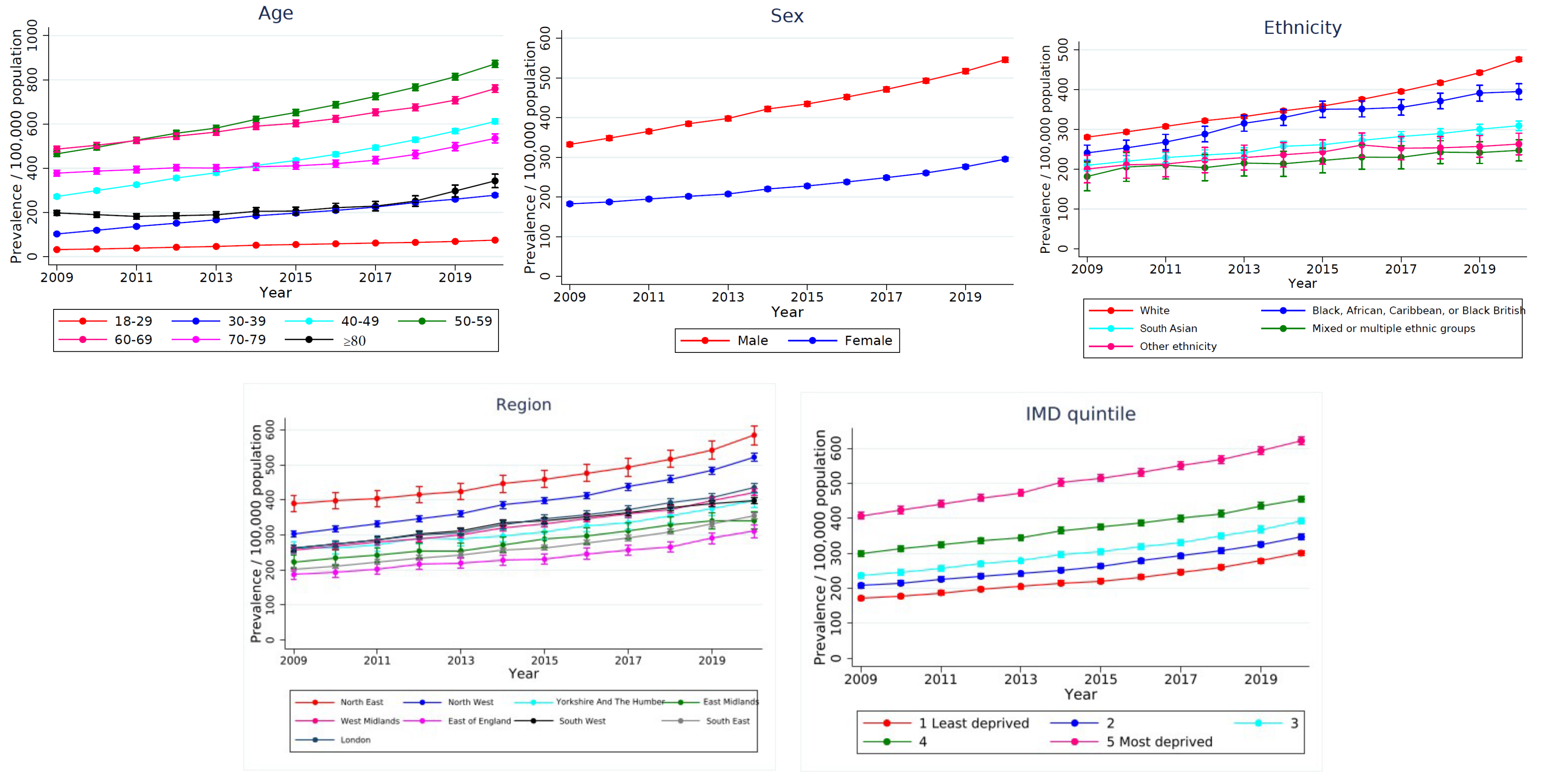

SFigure 4. Annual prevalence trends of Possible ARLD from 2009 to 2020, by age, sex, ethnicity, region and IMD quintile

ARLD = alcohol related liver disease; IMD = Index of Multiple Deprivation. (95% confidence intervals are drawn but are too small to be visible.) Northern Ireland and missing region are omitted from the region plot due to very small numbers of practices and patients.

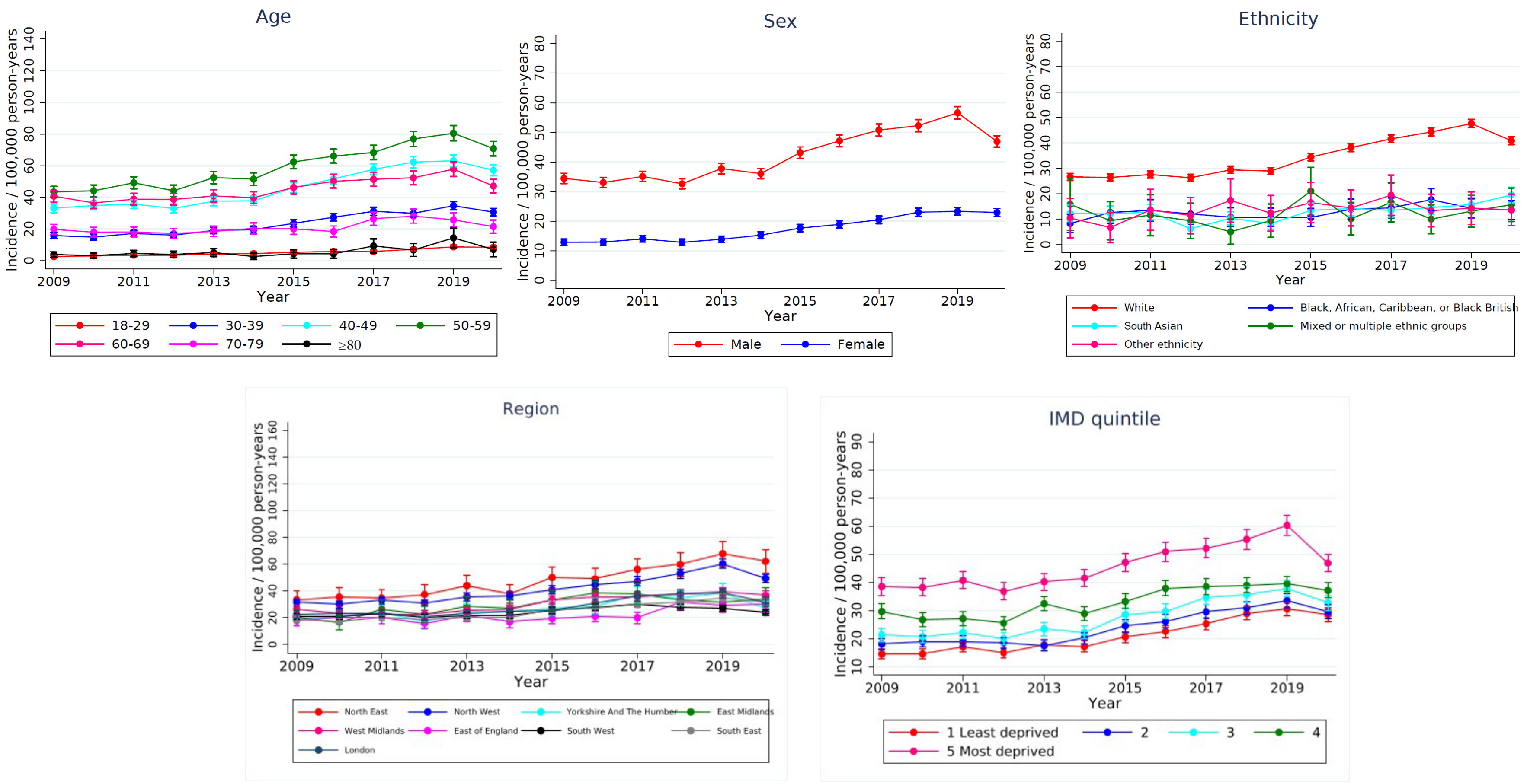

SFigure 5. Annual incidence trends of Probable ARLD from 2009 to 2020, by age, sex, ethnicity, region and IMD quintile

ARLD = alcohol related liver disease; IMD = Index of Multiple Deprivation. (95% confidence intervals are drawn but are too small to be visible.) Northern Ireland and missing region are omitted from the region plot due to very small numbers of practices and patients.

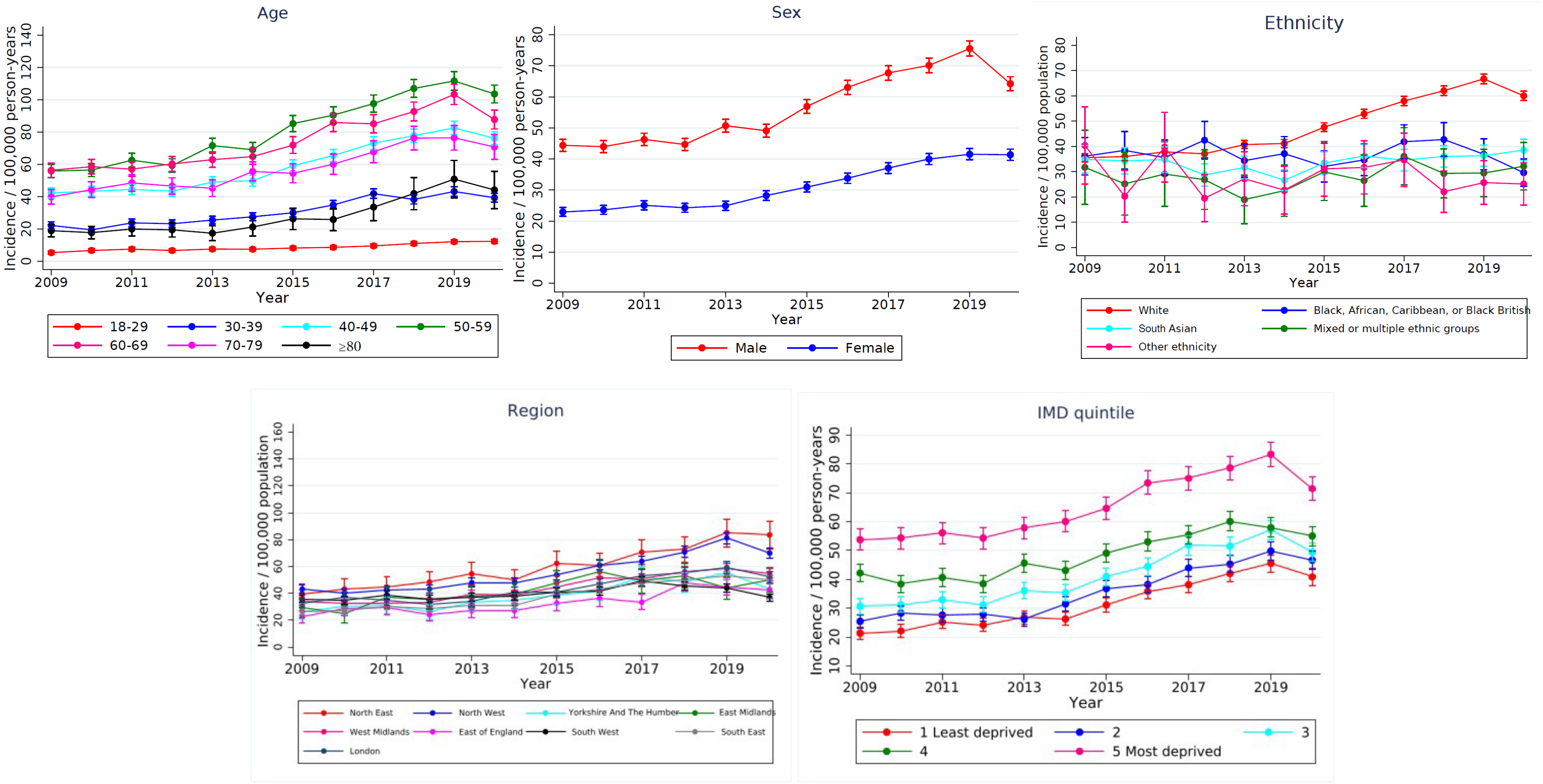

SFigure 6. Annual incidence trends of Possible ARLD from 2009 to 2020, by age, sex, ethnicity, region and IMD quintile

ARLD = alcohol related liver disease; IMD = Index of Multiple Deprivation. (95% confidence intervals are drawn but are too small to be visible.) Northern Ireland and missing region are omitted from the region plot due to very small numbers of practices and patients.

STable 8. Annual Prevalence of Definite ARLD (per 100,000 population)

	2009	2010	2011	2012	2013	2014	2015	2016	2017	2018	2019	2020
**Overall**												
Number of Patients	13,424	14,137	14,808	15,633	16,125	16,709	17,420	18,469	19,771	21,113	22,548	23,679
Prevalence	154	160	166	173	177	187	193	200	209	218	230	243
**Age, years**												
18-29												
Number of Patients	106	148	185	236	284	342	403	487	576	656	773	919
Prevalence	6	8	9	11	13	15	17	19	22	23	26	31
30-39												
Number of Patients	851	1,026	1,190	1,371	1,532	1,700	1,862	2,084	2,393	2,746	3,032	3,341
Prevalence	53	63	72	81	89	100	107	116	128	141	152	165
40-49												
Number of Patients	2,976	3,288	3,600	3,919	4,187	4,412	4,729	5,129	5,593	6,106	6,647	7,038
Prevalence	173	190	208	225	239	258	275	293	315	338	366	392
50-59												
Number of Patients	4,112	4,363	4,623	4,875	5,002	5,211	5,434	5,750	6,098	6,463	6,853	7,117
Prevalence	310	329	351	371	381	405	421	439	460	481	508	537
60-69												
Number of Patients	3,531	3,563	3,579	3,669	3,662	3,707	3,744	3,819	3,940	3,980	4,054	4,089
Prevalence	314	321	326	338	340	352	358	363	374	379	389	407
70-79												
Number of Patients	1,437	1,397	1,343	1,302	1,219	1,138	1,070	1,041	1,022	1,023	1,046	1,033
Prevalence	187	187	186	186	180	179	175	175	178	185	198	210
≥80												
Number of Patients	411	352	288	261	239	199	178	159	149	139	143	142
Prevalence	76	72	66	66	68	72	73	73	77	80	92	103
**Sex**												
Male												
Number of Patients	9,369	9,891	10,395	11,037	11,431	11,852	12,350	13,050	14,000	14,983	15,956	16,670
Prevalence	215	224	233	246	252	266	273	283	296	309	324	340
Female												
Number of Patients	4,055	4,246	4,413	4,596	4,694	4,857	5,070	5,419	5,771	6,130	6,592	7,009
Prevalence	93	96	98	101	102	108	112	117	122	127	135	144
**Ethnicity**												
White												
Number of Patients	9,064	10,076	10,927	11,799	12,410	13,139	13,818	14,782	16,029	17,284	18,622	19,741
Prevalence	174	187	195	204	209	217	225	234	247	259	274	292
Black, African, Caribbean, or Black British												
Number of Patients	194	196	207	236	250	267	299	321	350	380	433	455
Prevalence	80	75	75	81	82	85	93	95	99	103	116	118
South Asian												
Number of Patients	324	354	387	433	459	499	511	543	609	685	743	814
Prevalence	76	75	75	77	75	80	79	80	86	91	96	100
Mixed or Multiple ethnic groups												
Number of Patients	33	44	43	51	55	57	65	84	90	105	111	124
Prevalence	62	72	64	71	71	69	74	89	87	92	89	92
Other ethnicity												
Number of Patients	49	55	63	71	80	91	102	116	122	132	143	150
Prevalence	77	77	81	84	89	96	102	108	106	107	109	110

Missing												
Number of Patients	3,760	3,412	3,181	3,043	2,871	2,656	2,625	2,623	2,571	2,527	2,496	2,395
Prevalence	138	135	137	140	139	153	156	159	158	156	158	157
**Region**												
North East												
Number of Patients	775	809	830	867	891	926	959	1,008	1,045	1,099	1,168	1,208
Prevalence	275	281	283	293	297	308	317	329	337	351	368	393
North West												
Number of Patients	3,147	3,291	3,472	3,634	3,756	3,885	4,072	4,280	4,622	4,874	5,214	5,648
Prevalence	190	197	207	215	222	236	245	253	269	279	294	317
Yorkshire And The Humber												
Number of Patients	530	533	555	597	593	620	649	705	735	780	850	834
Prevalence	166	165	170	181	177	185	191	201	205	212	226	238
East Midlands												
Number of Patients	309	330	343	367	355	381	412	442	475	519	535	435
Prevalence	146	153	156	164	157	169	181	189	199	210	209	202
West Midlands												
Number of Patients	2,243	2,395	2,458	2,551	2,667	2,737	2,835	3,031	3,218	3,417	3,662	3,909
Prevalence	161	170	174	179	186	193	198	205	214	224	240	253
East of England												
Number of Patients	402	422	436	471	488	526	526	571	609	636	687	677
Prevalence	105	108	111	118	120	130	128	137	143	148	160	172
South West												
Number of Patients	2,215	2,331	2,450	2,621	2,701	2,821	2,947	3,145	3,437	3,729	3,963	4,140
Prevalence	144	148	152	161	161	170	175	180	189	195	201	206
South East												
Number of Patients	2,079	2,181	2,316	2,493	2,588	2,666	2,773	2,965	3,164	3396	3,698	3,916
Prevalence	115	119	126	134	138	144	148	156	165	174	186	196
London												
Number of Patients	1,637	1,757	1,856	1,933	1,989	2,052	2,151	2,222	2,361	2,562	2,673	2,796
Prevalence	149	158	165	171	174	191	200	206	215	228	238	251
Northern Ireland												
Number of Patients	75	76	83	94	90	89	91	95	101	97	98	113
Prevalence	207	205	220	246	232	226	227	232	241	227	225	253
Missing												
Number of Patients	12	12	9	5	7	6	5	5	4	4	0	3
Prevalence	314	341	281	189	323	376	390	384	304	310	0	1215
**IMD quintile**												
1 - Least deprived												
Number of Patients	1,727	1,778	1,873	1,979	2,046	2,145	2,204	2,354	2,568	2,774	3,017	3,233
Prevalence	96	97	102	107	110	117	119	125	134	143	153	166
2												
Number of Patients	2,108	2,249	2,364	2,472	2,560	2,626	2,787	2,981	3,205	3,452	3,715	3,849
Prevalence	119	125	131	135	139	144	152	159	168	177	187	197
3												
Number of Patients	2,268	2,406	2,521	2,692	2,811	2,911	3,042	3,256	3,492	3,797	4,056	4,267
Prevalence	138	144	149	158	163	170	176	183	192	203	213	227
4												
Number of Patients	2,995	3,165	3,299	3,484	3,568	3,718	3,875	4,077	4,346	4,632	4,963	5,128
Prevalence	180	187	192	200	201	212	217	222	230	237	248	256
5 - Most deprived												

Number of Patients	4,032	4,229	4,438	4,659	4,775	4,943	5,152	5,434	5,777	6,055	6,388	6,746
Prevalence	259	267	278	288	290	306	314	323	335	341	358	374
Missing												
Number of Patients	294	310	313	347	365	366	360	367	383	403	409	456
Prevalence	104	109	112	124	134	171	178	191	211	227	234	257

Number of Patients = number of patients with a record of alcohol-related liver disease; IMD = Index of Multiple Deprivation·

STable 9. Annual Prevalence of Probable ARLD (per 100,000 population)

	2009	2010	2011	2012	2013	2014	2015	2016	2017	2018	2019	2020
**Overall**												
Number of Patients	16,621	17,577	18,474	19,500	20,247	21,040	21,881	23,251	24,806	26,552	28,386	29,907
Prevalence	191	198	207	216	222	235	242	252	263	275	289	306
**Age, years**												
18-29												
Number of Patients	159	207	254	308	365	442	508	599	701	801	942	1,108
Prevalence	10	11	13	15	16	19	21	24	26	29	32	37
30-39												
Number of Patients	1,065	1,281	1,483	1,704	1,901	2,109	2,309	2,562	2,909	3,339	3,676	4,046
Prevalence	67	78	90	101	111	124	133	143	156	172	184	200
40-49												
Number of Patients	3,686	4,070	4,476	4,880	5,223	5,533	5,878	6,355	6,900	7,541	8,190	8,682
Prevalence	214	235	258	281	298	324	342	363	388	418	450	484
50-59												
Number of Patients	5,099	5,410	5,733	6,051	6,255	6,503	6,797	7,235	7,677	8,135	8,664	9,014
Prevalence	384	408	435	460	476	505	527	552	579	606	642	680
60-69												
Number of Patients	4,294	4,383	4,439	4,542	4,613	4,696	4,727	4,871	5,035	5,150	5,278	5,425
Prevalence	382	394	405	418	428	446	452	463	478	490	507	539
70-79												
Number of Patients	1,819	1,791	1,734	1,694	1,595	1,503	1,440	1,429	1,401	1,405	1,452	1,437
Prevalence	236	239	240	242	236	236	235	240	244	254	275	292
≥80												
Number of Patients	499	435	355	321	295	254	222	200	183	182	185	196
Prevalence	92	89	81	81	83	92	91	92	95	105	119	142
**Sex**												
Male												
Number of Patients	11,704	12,441	13,117	13,900	14,489	15,080	15,677	16,621	17,735	19,025	20,229	21,236
Prevalence	269	282	295	309	319	338	347	360	376	393	411	433
Female												
Number of Patients	4,917	5,136	5,357	5,600	5,758	5,960	6,204	6,630	7,071	7,527	8,157	8,671
Prevalence	112	116	120	124	126	133	137	143	150	156	167	179
**Ethnicity**												
White												
Number of Patients	11,344	12,587	13,686	14,749	15,600	16,566	17,413	18,673	20,151	21,738	23,464	24,968
Prevalence	218	230	241	252	260	271	280	293	308	325	344	369
Black, African, Caribbean, or Black British												
Number of Patients	242	252	274	307	335	362	394	412	445	498	559	574
Prevalence	100	96	99	106	110	116	122	122	126	135	149	149
South Asian												
Number of Patients	403	449	487	542	561	614	630	678	761	852	924	1012
Prevalence	95	96	95	98	94	98	98	101	107	113	118	124
Mixed or Multiple ethnic groups												
Number of Patients	38	58	58	68	74	75	83	105	114	132	146	163
Prevalence	71	95	86	94	95	91	94	111	111	115	117	121
Other ethnicity												
Number of Patients	57	66	75	88	104	118	127	147	152	166	177	187
Prevalence	90	93	96	104	115	124	127	137	132	135	135	137

Missing												
Number of Patients	4,537	4,165	3,894	3,746	3,573	3,305	3,234	3,236	3,183	3,167	3,117	3,004
Prevalence	166	165	167	172	174	191	193	196	196	196	197	197
**Region**												
North East												
Number of Patients	934	978	1,012	1,048	1,090	1,146	1,188	1,247	1,311	1,380	1,482	1,545
Prevalence	331	339	345	354	364	381	392	407	423	440	467	503
North West												
Number of Patients	3,950	4,159	4,383	4,584	4,779	4,964	5,163	5,461	5,877	6,233	6,691	7,269
Prevalence	238	249	261	272	283	301	311	323	342	357	377	407
Yorkshire And The Humber												
Number of Patients	636	644	671	725	724	759	797	870	907	978	1,070	1,053
Prevalence	199	199	206	220	216	227	235	249	253	266	285	301
East Midlands												
Number of Patients	374	399	410	434	426	456	484	518	561	612	645	548
Prevalence	177	185	186	194	188	202	213	222	234	248	252	255
West Midlands												
Number of Patients	2,701	2,886	2,991	3,102	3,254	3,375	3,502	3,776	3,996	4,232	4,502	4,820
Prevalence	194	204	212	218	227	239	245	256	266	277	295	311
East of England												
Number of Patients	500	531	558	609	620	656	661	712	762	794	877	869
Prevalence	130	136	142	152	153	162	161	171	179	184	204	221
South West												
Number of Patients	2,744	2,895	3,080	3,302	3,423	3,603	3,735	3,968	4,311	4,692	4,982	5,166
Prevalence	179	184	191	202	204	218	222	227	237	246	253	257
South East												
Number of Patients	2,605	2,765	2,929	3,142	3,299	3,413	3,541	3,791	4,005	4,297	4,668	4,993
Prevalence	145	151	159	169	176	184	189	200	208	220	234	250
London												
Number of Patients	2,076	2,218	2,331	2,440	2,515	2,550	2,693	2,786	2,947	3,201	3,329	3,482
Prevalence	189	199	208	215	220	237	250	258	268	285	296	313
Northern Ireland												
Number of Patients	88	89	99	108	108	110	112	117	125	129	140	158
Prevalence	243	240	263	283	278	279	279	286	299	302	321	354
Missing												
Number of Patients	13	13	10	6	9	8	5	5	4	4	0	4
Prevalence	340	370	313	226	415	501	390	384	304	310	0	1619
**IMD quintile**												
1 - Least deprived												
Number of Patients	2,177	2,271	2,387	2,537	2,626	2,744	2,837	3,056	3,274	3,534	3,824	4,133
Prevalence	121	124	130	138	141	149	153	162	171	182	194	212
2												
Number of Patients	2,618	2,776	2,940	3,101	3,222	3,313	3,502	3,772	4,054	4,366	4,701	4,910
Prevalence	148	154	162	170	175	182	191	201	212	224	237	251
3												
Number of Patients	2,834	3,007	3,177	3,355	3,522	3,669	3,836	4,098	4,348	4,729	5,068	5,324
Prevalence	172	180	188	196	204	214	221	231	239	253	266	283
4												
Number of Patients	3,685	3,933	4,127	4,371	4,505	4,717	4,894	5,149	5,500	5,879	6,288	6,525
Prevalence	222	232	240	250	254	269	274	281	291	301	315	326
5 - Most deprived												

Number of Patients	4,960	5,222	5,473	5,732	5,940	6,160	6,379	6,736	7,169	7,547	7,987	8,436
Prevalence	318	330	343	354	361	382	389	401	415	425	447	468
Missing												
Number of Patients	347	368	370	404	432	437	433	440	461	498	519	580
Prevalence	122	129	132	145	158	204	214	229	253	281	297	327

Number of Patients = number of patients with a record of alcohol-related liver disease; IMD = Index of Multiple Deprivation·

STable 10. Annual Prevalence of Possible ARLD (per 100,000 population)

	2009	2010	2011	2012	2013	2014	2015	2016	2017	2018	2019	2020
**Overall**												
Number of Patients	22,455	23,712	25,002	26,422	27,601	28,720	29,943	31,861	34,007	36,457	38,953	41,117
Prevalence	257	268	280	293	302	321	331	345	360	377	397	421
**Age, years**												
18-29												
Number of Patients	517	623	749	889	1,024	1,180	1,304	1,457	1,627	1,795	1,998	2,218
Prevalence	32	34	38	42	46	51	55	58	61	64	68	74
30-39												
Number of Patients	1,634	1,940	2,254	2,535	2,853	3,138	3,418	3,753	4,173	4,743	5,181	5,608
Prevalence	102	119	136	151	166	185	197	209	224	245	260	278
40-49												
Number of Patients	4,685	5,179	5,641	6,187	6,640	7,049	7,484	8,098	8,769	9,548	10,329	10,982
Prevalence	272	299	325	356	379	413	435	463	494	529	568	612
50-59												
Number of Patients	6,174	6,551	6,947	7,337	7,638	7,994	8,412	9,005	9,616	10,288	10,982	11,561
Prevalence	465	494	527	558	581	621	652	687	725	766	814	872
60-69												
Number of Patients	5,472	5,599	5,763	5,923	6,072	6,208	6,309	6,569	6,872	7,092	7,372	7,643
Prevalence	486	504	525	545	564	590	603	624	653	675	708	760
70-79												
Number of Patients	2,908	2,893	2,850	2,818	2,706	2,585	2,513	2,500	2,508	2,558	2,631	2,633
Prevalence	377	387	394	402	400	406	411	420	436	462	497	535
≥80												
Number of Patients	1,065	927	798	733	668	566	503	479	442	434	461	473
Prevalence	197	190	182	185	189	205	206	221	228	251	297	342
**Sex**												
Male												
Number of Patients	14,470	15,385	16,273	17,277	18,088	18,826	19,638	20,864	22,264	23,890	25,450	26,765
Prevalence	333	348	365	385	398	422	435	452	471	493	517	546
Female												
Number of Patients	7,985	8,327	8,729	9,145	9,513	9,894	10,305	10,997	11,743	12,567	13,503	14,352
Prevalence	183	187	195	202	208	220	228	238	249	261	276	295
**Ethnicity**												
White												
Number of Patients	14,619	16,067	17,436	18,811	19,912	21,164	22,266	23,928	25,820	27,922	30,166	32,182
Prevalence	280	294	308	322	332	347	359	375	395	417	442	476
Black, African, Caribbean, or Black British												
Number of Patients	586	664	741	836	960	1,030	1,130	1,183	1,252	1,366	1,465	1,520
Prevalence	241	254	268	288	315	330	350	351	355	371	391	395
South Asian												
Number of Patients	892	1,034	1,178	1,308	1,447	1,607	1,686	1,834	2,005	2,179	2,348	2,514
Prevalence	210	220	229	236	241	258	261	272	282	290	300	309
Mixed or Multiple ethnic groups												
Number of Patients	97	125	141	147	167	176	195	218	237	278	302	333
Prevalence	182	205	210	204	215	214	222	230	230	243	242	247
Other ethnicity												
Number of Patients	127	150	166	188	207	225	244	279	290	312	336	359
Prevalence	200	211	213	223	229	236	243	261	253	254	257	263

Missing												
Number of Patients	6,134	5,672	5,340	5,132	4,908	4,518	4,422	4,419	4,403	4,401	4,337	4,210
Prevalence	225	225	229	236	238	261	264	267	270	272	274	276
**Region**												
North East												
Number of Patients	1,102	1,144	1,188	1,229	1,269	1,341	1,390	1,459	1,527	1,619	1,718	1,793
Prevalence	391	397	405	415	423	446	459	477	493	517	541	584
North West												
Number of Patients	5,032	5,315	5,560	5,835	6,111	6,363	6,609	6,979	7,522	8,007	8,580	9,317
Prevalence	304	318	332	346	361	386	398	413	438	459	484	522
Yorkshire And The Humber												
Number of Patients	840	849	888	960	964	994	1,046	1,142	1,198	1,302	1,408	1,391
Prevalence	263	263	272	292	288	297	308	326	334	354	374	397
East Midlands												
Number of Patients	474	508	532	569	579	615	653	694	746	815	869	733
Prevalence	224	235	242	255	255	272	288	297	312	330	340	341
West Midlands												
Number of Patients	3,595	3,790	3,951	4,100	4,320	4,537	4,744	5,106	5,405	5,701	6,074	6,535
Prevalence	258	269	280	288	301	321	332	346	359	373	398	422
East of England												
Number of Patients	719	754	797	869	894	924	944	1,026	1,095	1,143	1,249	1,220
Prevalence	187	193	203	217	220	228	230	246	257	265	291	310
South West												
Number of Patients	4,042	4,314	4,620	4,962	5,239	5,518	5,743	6,148	6,633	7,213	7,690	7,992
Prevalence	263	274	287	304	313	333	341	352	364	378	390	398
South East												
Number of Patients	3,650	3,852	4,101	4,358	4,566	4,732	4,924	5,264	5,606	6,053	6,595	7,080
Prevalence	203	211	223	234	243	256	263	277	292	309	331	355
London												
Number of Patients	2,867	3,051	3,220	3,387	3,498	3,533	3,729	3,877	4,100	4,416	4,577	4,827
Prevalence	261	274	287	299	306	328	346	359	373	393	407	434
Northern Ireland												
Number of Patients	117	117	130	141	146	149	153	158	168	180	193	225
Prevalence	323	316	345	369	376	378	381	386	401	421	443	504
Missing												
Number of Patients	17	18	15	12	15	14	8	8	7	8	0	4
Prevalence	445	512	469	453	692	877	624	614	532	620	0	1619
**IMD quintile**												
1 - Least deprived												
Number of Patients	3,097	3,244	3,437	3,650	3,818	3,954	4,079	4,385	4,718	5,067	5,475	5,861
Prevalence	172	178	188	198	205	215	221	233	246	261	278	301
2												
Number of Patients	3,687	3,872	4,092	4,297	4,466	4,584	4,843	5,217	5,584	6,005	6,460	6,771
Prevalence	208	215	226	235	242	252	264	278	292	308	326	347
3												
Number of Patients	3,889	4,106	4,344	4,610	4,820	5,072	5,297	5,653	6,023	6,548	7,007	7,385
Prevalence	236	245	257	270	279	296	306	318	331	351	368	393
4												
Number of Patients	4,975	5,297	5,573	5,886	6,124	6,418	6,690	7,103	7,557	8,090	8,705	9,083
Prevalence	300	313	324	337	345	366	375	388	400	414	436	454
5 - Most deprived												

Number of Patients	6,337	6,701	7,046	7,423	7,778	8,096	8,450	8,912	9,504	10,076	10,600	11,209
Prevalence	407	424	441	458	473	502	515	530	550	568	594	622
Missing												
Number of Patients	470	492	510	556	595	596	584	591	621	672	707	809
Prevalence	166	173	182	199	218	278	289	308	341	379	404	456

Number of Patients = number of patients with a record of alcohol-related liver disease; IMD = Index of Multiple Deprivation.

STable 11. Annual Incidence of Definite ARLD (per 100,000 person-years)

	2009	2010	2011	2012	2013	2014	2015	2016	2017	2018	2019	2020
**Overall**												
Number of Patients	1,630	1,565	1,761	1,547	1,729	1,759	2,117	2,482	2,610	2,822	2,949	2,386
Person years	8760375	8856617	8933468	9062655	8976372	8949984	9095383	9323846	9515972	9710170	9718049	9677562
Incidence	18·6	17·7	19·7	17·1	19·3	19·7	23·3	26·6	27·4	29·1	30·3	24·7
**Age, years**												
18-29												
Number of Patients	38	46	63	65	78	96	114	129	133	172	216	220
Person years	1716554	1878541	2018954	2163666	2243507	2323683	2434846	2574455	2713683	2854568	2928470	2999310
Incidence	2·2	2·4	3·1	3·0	3·5	4·1	4·7	5·0	4·9	6·0	7·4	7·3
30-39												
Number of Patients	221	206	240	216	264	274	356	432	505	498	577	519
Person years	1612999	1639019	1660968	1698797	1697605	1713282	1762100	1829752	1897474	1966293	1997153	2026350
Incidence	13·7	12·6	14·4	12·7	15·6	16·0	20·2	23·6	26·6	25·3	28·9	25·6
40-49												
Number of Patients	469	478	503	458	492	530	648	773	846	924	921	741
Person years	1724686	1728663	1730200	1744517	1719564	1706876	1728550	1761529	1784586	1806589	1794734	1772960
Incidence	27·2	27·7	29·1	26·3	28·6	31·1	37·5	43·9	47·4	51·1	51·3	41·8
50-59												
Number of Patients	464	461	527	453	518	503	601	690	697	779	801	627
Person years	1322050	1315940	1309635	1311713	1292719	1281172	1294566	1316344	1328263	1339530	1327229	1303905
Incidence	35·1	35·0	40·2	34·5	40·1	39·3	46·4	52·4	52·5	58·2	60·4	48·1
60-69												
Number of Patients	328	288	334	287	295	299	334	389	352	358	361	240
Person years	1114478	1099035	1087202	1080510	1059366	1043702	1045084	1051586	1047423	1041543	1016776	978800
Incidence	29·4	26·2	30·7	26·6	27·8	28·6	32·0	37·0	33·6	34·4	35·5	24·5
70-79												
Number of Patients	102	80	84	62	77	55	59	66	70	86	67	34
Person years	757409	733529	710445	688316	652842	621979	601649	585104	562420	539151	507884	469162
Incidence	13·5	10·9	11·8	9·0	11·8	8·8	9·8	11·3	12·4	16·0	13·2	7·2
≥80												
Number of Patients	8	6	10	6	5	2	5	3	7	5	6	5
Person years	512199	461889	416064	375135	310771	259291	228589	205076	182124	162496	145803	127076
Incidence	1·6	1·3	2·4	1·6	1·6	0·8	2·2	1·5	3·8	3·1	4·1	3·9
**Sex**												
Male												
Number of Patients	1,147	1,103	1,254	1,081	1,234	1,199	1,460	1,762	1,836	1,933	2,037	1,600
Person years	4366445	4413585	4445673	4508513	4464582	4466236	4541507	4657945	4760915	4865632	4876208	4854790
Incidence	26·3	25·0	28·2	24·0	27·6	26·8	32·1	37·8	38·6	39·7	41·8	33·0
Female												
Number of Patients	483	462	507	466	495	560	657	720	774	889	912	786
Person years	4393931	4443033	4487796	4554143	4511791	4483748	4553876	4665901	4755058	4844538	4841841	4822773
Incidence	11·0	10·4	11·3	10·2	11·0	12·5	14·4	15·4	16·3	18·4	18·8	16·3
**Ethnicity**												
White												
Number of Patients	1,121	1,115	1,264	1,165	1,329	1,346	1,648	1,988	2,135	2,292	2,406	1,909
Person years	5331004	5550618	5731102	5912421	6027102	6129431	6264803	6445836	6588589	6729208	6745736	6708327
Incidence	21·0	20·1	22·1	19·7	22·1	22·0	26·3	30·8	32·4	34·1	35·7	28·5
Black, African, Caribbean, or Black British												

Number of Patients	15	27	33	28	23	28	31	42	31	49	52	39
Person years	252218	268307	281755	297237	305983	315644	328031	343968	359299	372965	377544	389098
Incidence	5·9	10·1	11·7	9·4	7·5	8·9	9·5	12·2	8·6	13·1	13·8	10·0
South Asian												
Number of Patients	41	50	58	32	44	40	61	77	81	92	103	126
Person years	444811	487590	529422	575240	606561	627964	652825	688827	727078	767326	791598	823791
Incidence	9·2	10·3	11·0	5·6	7·3	6·4	9·3	11·2	11·1	12·0	13·0	15·3
Mixed or Multiple ethnic groups												
Number of Patients	5	3	8	5	2	6	15	9	12	7	14	17
Person years	56786	63498	68857	74513	79090	84477	90373	98280	108204	119526	129010	140081
Incidence	8·8	4·7	11·6	6·7	2·5	7·1	16·6	9·2	11·1	5·9	10·9	12·1
Other ethnicity												
Number of Patients	5	6	9	8	12	10	12	15	17	12	19	15
Person years	66954	73997	80650	87180	91971	96999	102885	110608	118339	126855	132464	139276
Incidence	7·5	8·1	11·2	9·2	13·0	10·3	11·7	13·6	14·4	9·5	14·3	10·8
Missing												
Number of Patients	443	364	389	309	319	329	350	351	334	370	355	280
Person years	2608602	2412608	2241683	2116064	1865666	1695470	1656466	1636327	1614463	1594290	1541697	1476990
Incidence	17·0	15·1	17·4	14·6	17·1	19·4	21·1	21·5	20·7	23·2	23·0	19·0
**Region**												
North East												
Number of Patients	76	84	82	82	97	87	114	120	120	139	148	126
Person years	283522	289407	293060	296733	297983	299552	302148	306462	309358	313191	308584	306506
Incidence	26·8	29·0	28·0	27·6	32·6	29·0	37·7	39·2	38·8	44·4	48·0	41·1
North West												
Number of Patients	413	393	448	375	443	480	513	591	608	693	798	591
Person years	1658181	1664019	1672539	1684098	1659528	1647046	1668903	1699966	1724114	1749624	1765744	1776888
Incidence	24·9	23·6	26·8	22·3	26·7	29·1	30·7	34·8	35·3	39·6	45·2	33·3
Yorkshire And The Humber												
Number of Patients	50	51	64	44	58	59	75	82	93	89	107	60
Person years	319443	322695	324505	330525	331942	334316	342367	352440	360662	369215	350914	324661
Incidence	15·7	15·8	19·7	13·3	17·5	17·6	21·9	23·3	25·8	24·1	30·5	18·5
East Midlands												
Number of Patients	35	33	52	37	50	53	56	69	75	57	51	57
Person years	211420	216079	219070	223463	223253	223224	227644	234666	239755	248481	231621	216181
Incidence	16·6	15·3	23·7	16·6	22·4	23·7	24·6	29·4	31·3	22·9	22·0	26·4
West Midlands												
Number of Patients	305	248	251	260	251	271	377	433	433	479	471	411
Person years	1399415	1403977	1409044	1428443	1415005	1415013	1448989	1489104	1509517	1535536	1532853	1523192
Incidence	21·8	17·7	17·8	18·2	17·7	19·2	26·0	29·1	28·7	31·2	30·7	27·0
East of England												
Number of Patients	48	54	55	53	71	51	67	68	68	98	78	75
Person years	387371	392028	395683	403200	405648	406832	412323	421766	427280	426427	411262	379686
Incidence	12·4	13·8	13·9	13·1	17·5	12·5	16·2	16·1	15·9	23·0	19·0	19·8
South West												
Number of Patients	249	246	306	258	275	293	338	414	462	425	446	375
Person years	1551273	1589181	1614175	1655290	1646258	1659121	1704204	1782123	1863350	1934994	1974618	2018835
Incidence	16·1	15·5	19·0	15·6	16·7	17·7	19·8	23·2	24·8	22·0	22·6	18·6
South East												
Number of Patients	243	244	290	256	270	269	362	417	441	491	503	451
Person years	1808478	1826141	1841181	1863503	1854466	1851996	1873576	1907297	1931440	1965081	1982310	1974083

Incidence	13·4	13·4	15·8	13·7	14·6	14·5	19·3	21·9	22·8	25·0	25·4	22·8
London												
Number of Patients	206	204	205	178	208	193	206	278	304	342	329	230
Person years	1101146	1112594	1123532	1136548	1101461	1071837	1073538	1087400	1107030	1123360	1116172	1111732
Incidence	18·7	18·3	18·2	15·7	18·9	18·0	19·2	25·6	27·5	30·4	29·5	20·7
Northern Ireland												
Number of Patients	4	8	8	2	6	2	9	10	6	9	18	10
Person years	36459	37158	37757	38455	38961	39650	40394	41309	42166	43090	43886	45323
Incidence	11·0	21·5	21·2	5·2	15·4	5·0	22·3	24·2	14·2	20·9	41·0	22·1
Missing												
Number of Patients	1	0	0	2	0	1	0	0	0	0	0	0
Person years	3668	3338	2922	2398	1868	1397	1299	1312	1302	1170	86	477
Incidence	27·3	0	0	83·4	0	71·6	0	0	0	0	0	0
**IMD quintile**												
1 - Least deprived												
Number of Patients	194	203	231	213	239	215	272	346	361	444	428	372
Person years	1810130	1823491	1831827	1849326	1840900	1835130	1859932	1898437	1923581	1949740	1945801	1923490
Incidence	10·7	11·1	12·6	11·5	13·0	11·7	14·6	18·2	18·8	22·8	22·0	19·3
2												
Number of Patients	256	253	258	249	238	277	341	397	425	474	481	395
Person years	1779569	1796687	1810796	1831184	1821282	1818491	1847315	1888701	1921260	1958634	1953765	1922433
Incidence	14·4	14·1	14·2	13·6	13·1	15·2	18·5	21·0	22·1	24·2	24·6	20·5
3												
Number of Patients	282	249	314	263	294	303	389	432	495	504	540	428
Person years	1653887	1675556	1691084	1716265	1710410	1713952	1745180	1793561	1834753	1876768	1882960	1865969
Incidence	17·1	14·9	18·6	15·3	17·2	17·7	22·3	24·1	27·0	26·9	28·7	22·9
4												
Number of Patients	374	363	380	340	421	392	454	556	562	596	605	529
Person years	1670237	1699740	1723233	1758335	1748852	1758491	1796038	1857135	1915417	1969747	1981683	1992305
Incidence	22·4	21·4	22·1	19·3	24·1	22·3	25·3	29·9	29·3	30·3	30·5	26·6
5 - Most deprived												
Number of Patients	490	471	543	446	497	533	616	702	725	759	834	609
Person years	1563298	1579352	1597539	1630820	1615487	1616724	1650864	1699466	1741880	1780782	1778479	1793834
Incidence	31·3	29·8	34·0	27·3	30·8	33·0	37·3	41·3	41·6	42·6	46·9	33·9
Missing												
Number of Patients	34	26	35	36	40	39	45	49	42	45	61	53
Person years	283255	281791	278991	276726	239442	207197	196054	186546	179082	174500	175361	179530
Incidence	12·0	9·2	12·5	13·0	16·7	18·8	23·0	26·3	23·5	25·8	34·8	29·5

Number of Patients = number of patients with a record of alcohol-related liver disease; Person years = total number of person years in the denominator population; IMD = Index of Multiple Deprivation·

STable 12. Annual Incidence of Probable ARLD (per 100,000 person-years)

	2009	2010	2011	2012	2013	2014	2015	2016	2017	2018	2019	2020
**Overall**												
Number of Patients	2,070	2,034	2,189	2,056	2,313	2,295	2,764	3,076	3,389	3,656	3,888	3,382
Person years	8757050	8853082	8929707	9058654	8972146	8945586	9090775	9318943	9510757	9704539	9712044	9671161
Incidence	23·6	23·0	24·5	22·7	25·8	25·7	30·4	33·0	35·6	37·7	40·0	35·0
**Age, years**												
18-29												
Number of Patients	44	58	71	76	94	105	129	151	158	206	256	255
Person years	1716525	1878509	2018919	2163630	2243458	2323625	2434790	2574405	2713636	2854526	2928436	2999280
Incidence	2·6	3·1	3·5	3·5	4·2	4·5	5·3	5·9	5·8	7·2	8·7	8·5
30-39												
Number of Patients	256	244	286	274	325	336	416	502	592	589	694	622
Person years	1612777	1638764	1660667	1698456	1697229	1712869	1761649	1829270	1896949	1965701	1996516	2025639
Incidence	15·9	14·9	17·2	16·1	19·1	19·6	23·6	27·4	31·2	30·0	34·8	30·7
40-49												
Number of Patients	572	603	618	576	646	645	798	910	1,029	1,124	1,132	1,012
Person years	1723955	1727852	1729299	1743534	1718494	1705767	1727391	1760277	1783242	1805121	1793163	1771264
Incidence	33·2	34·9	35·7	33·0	37·6	37·8	46·2	51·7	57·7	62·3	63·1	57·1
50-59												
Number of Patients	574	581	644	579	678	660	807	869	907	1,029	1,067	922
Person years	1321040	1314881	1308509	1310507	1291463	1279849	1293158	1314826	1326639	1337791	1325377	1301958
Incidence	43·5	44·2	49·2	44·2	52·5	51·6	62·4	66·1	68·4	76·9	80·5	70·8
60-69												
Number of Patients	454	401	422	418	433	415	483	527	538	545	587	461
Person years	1113693	1098206	1086347	1079607	1058407	1042718	1044072	1050528	1046308	1040356	1015505	977444
Incidence	40·8	36·5	38·8	38·7	40·9	39·8	46·3	50·2	51·4	52·4	57·8	47·2
70-79												
Number of Patients	150	132	129	118	121	127	121	108	149	152	131	101
Person years	757028	733144	710061	687937	652470	621605	601267	584717	562038	538757	507486	468770
Incidence	19·8	18·0	18·2	17·2	18·5	20·4	20·1	18·5	26·5	28·2	25·8	21·5
≥80												
Number of Patients	20	15	19	15	16	7	10	9	17	11	21	9
Person years	512123	461822	416006	375086	310719	259245	228549	205038	182085	162457	145758	127027
Incidence	3·9	3·2	4·6	4·0	5·1	2·7	4·4	4·4	9·3	6·8	14·4	7·1
**Sex**												
Male												
Number of Patients	1,504	1,458	1,560	1,470	1,686	1,610	1,959	2,194	2,416	2,541	2,758	2,276
Person years	4364004	4410966	4442882	4505547	4461447	4462956	4538074	4654304	4757042	4861474	4871811	4850126
Incidence	34·5	33·1	35·1	32·6	37·8	36·1	43·2	47·1	50·8	52·3	56·6	46·9
Female												
Number of Patients	566	576	629	586	627	685	805	882	973	1,115	1,130	1,106
Person years	4393047	4442117	4486826	4553107	4510699	4482631	4552702	4664639	4753715	4843065	4840234	4821036
Incidence	12·9	13·0	14·0	12·9	13·9	15·3	17·7	18·9	20·5	23·0	23·3	22·9
**Ethnicity**												
White												
Number of Patients	1,422	1,467	1,580	1,556	1,774	1,774	2,157	2,460	2,741	2,982	3,211	2,741
Person years	5328642	5548044	5728306	5909391	6023843	6125979	6261135	6441918	6584409	6724683	6740853	6703081
Incidence	26·7	26·4	27·6	26·3	29·4	29·0	34·5	38·2	41·6	44·3	47·6	40·9
Black, African, Caribbean, or Black British												

Number of Patients	21	34	38	36	33	34	35	48	52	66	54	53
Person years	252168	268247	281688	297160	305893	315549	327941	343877	359198	372843	377422	388975
Incidence	8·3	12·7	13·5	12·1	10·8	10·8	10·7	14·0	14·5	17·7	14·3	13·6
South Asian												
Number of Patients	55	59	68	36	63	52	88	98	98	112	125	161
Person years	444722	487497	529318	575138	606458	627850	652698	688688	726926	767158	791414	823592
Incidence	12·4	12·1	12·8	6·3	10·4	8·3	13·5	14·2	13·5	14·6	15·8	19·5
Mixed or Multiple ethnic groups												
Number of Patients	9	6	8	7	4	8	19	10	18	12	17	22
Person years	56779	63483	68843	74498	79073	84463	90356	98260	108181	119494	128974	140044
Incidence	15·9	9·5	11·6	9·4	5·1	9·5	21·0	10·2	16·6	10·0	13·2	15·7
Other ethnicity												
Number of Patients	7	5	11	10	16	12	17	16	23	17	19	19
Person years	66943	73985	80634	87160	91946	96975	102856	110581	118308	126824	132434	139242
Incidence	10·5	6·8	13·6	11·5	17·4	12·4	16·5	14·5	19·4	13·4	14·3	13·6
Missing												
Number of Patients	556	463	484	411	423	415	448	444	458	467	462	386
Person years	2607888	2411923	2241021	2115411	1865027	1694862	1655891	1635738	1613874	1593707	1541143	1476448
Incidence	21·3	19·2	21·6	19·4	22·7	24·5	27·1	27·1	28·4	29·3	30·0	26·1
**Region**												
North East												
Number of Patients	95	102	101	111	131	114	151	150	173	187	209	190
Person years	283360	289235	292879	296542	297777	299327	301916	306208	309085	312897	308263	306147
Incidence	33·5	35·3	34·5	37·4	44·0	38·1	50·0	49·0	56·0	59·8	67·8	62·1
North West												
Number of Patients	523	498	550	520	592	594	679	756	816	929	1065	877
Person years	1657341	1663128	1671607	1683108	1658465	1645957	1667772	1698749	1722810	1748219	1764206	1775217
Incidence	31·6	29·9	32·9	30·9	35·7	36·1	40·7	44·5	47·4	53·1	60·4	49·4
Yorkshire And The Humber												
Number of Patients	59	67	77	57	83	83	93	102	133	127	136	88
Person years	319336	322583	324384	330396	331807	334170	342213	352269	360475	369007	350702	324455
Incidence	18·5	20·8	23·7	17·3	25·0	24·8	27·2	29·0	36·9	34·4	38·8	27·1
East Midlands												
Number of Patients	43	36	58	50	63	61	75	91	91	83	73	74
Person years	211357	216013	219004	223397	223182	223151	227572	234587	239664	248381	231507	216062
Incidence	20·3	16·7	26·5	22·4	28·2	27·3	33·0	38·8	38·0	33·4	31·5	34·2
West Midlands												
Number of Patients	370	328	312	324	359	375	480	528	538	584	607	559
Person years	1398940	1403467	1408499	1427871	1414396	1414358	1448275	1488344	1508730	1534703	1531976	1522251
Incidence	26·4	23·4	22·2	22·7	25·4	26·5	33·1	35·5	35·7	38·1	39·6	36·7
East of England												
Number of Patients	69	78	79	64	88	68	80	89	85	136	121	113
Person years	387263	391914	395554	403065	405515	406695	412186	421622	427126	426250	411069	379494
Incidence	17·8	19·9	20·0	15·9	21·7	16·7	19·4	21·1	19·9	31·9	29·4	29·8
South West												
Number of Patients	322	337	378	335	361	365	432	492	563	542	537	481
Person years	1550723	1588587	1613529	1654590	1645505	1658345	1703397	1781267	1862445	1933998	1973602	2017799
Incidence	20·8	21·2	23·4	20·2	21·9	22·0	25·4	27·6	30·2	28·0	27·2	23·8
South East												
Number of Patients	337	317	366	351	368	369	490	519	577	626	692	624
Person years	1807921	1825544	1840548	1862828	1853744	1851234	1872786	1906477	1930567	1964149	1981293	1972976

Incidence	18·6	17·4	19·9	18·8	19·9	19·9	26·2	27·2	29·9	31·9	34·9	31·6
London												
Number of Patients	247	262	257	235	258	262	271	337	399	422	427	355
Person years	1100697	1112130	1123042	1136024	1100948	1071323	1072987	1086824	1106417	1122712	1115499	1111011
Incidence	22·4	23·6	22·9	20·7	23·4	24·5	25·3	31·0	36·1	37·6	38·3	32·0
Northern Ireland												
Number of Patients	4	9	10	7	10	3	13	12	14	20	21	21
Person years	36446	37144	37742	38438	38941	39629	40373	41286	42138	43053	43842	45275
Incidence	11·0	24·2	26·5	18·2	25·7	7·6	32·2	29·1	33·2	46·5	47·9	46·4
Missing												
Number of Patients	1	0	1	2	0	1	0	0	0	0	0	0
Person years	3667	3337	2921	2396	1866	1396	1299	1312	1302	1170	85	475
Incidence	27·3	0	34·2	83·5	0·0	71·6	0	0	0	0	0	0
**IMD quintile**												
1 - Least deprived												
Number of Patients	262	265	312	278	327	316	382	429	486	564	593	548
Person years	1809663	1822996	1831302	1848773	1840318	1834515	1859276	1897750	1922870	1948985	1944981	1922586
Incidence	14·5	14·5	17·0	15·0	17·8	17·2	20·5	22·6	25·3	28·9	30·5	28·5
2												
Number of Patients	321	340	344	338	319	367	452	492	566	604	654	569
Person years	1779055	1796156	1810209	1830543	1820625	1817805	1846585	1887903	1920408	1957713	1952779	1921378
Incidence	18·0	18·9	19·0	18·5	17·5	20·2	24·5	26·1	29·5	30·9	33·5	29·6
3												
Number of Patients	351	346	376	342	400	381	499	533	636	669	710	610
Person years	1653315	1674941	1690443	1715603	1709693	1713190	1744381	1792727	1833886	1875825	1881966	1864923
Incidence	21·2	20·7	22·2	19·9	23·4	22·2	28·6	29·7	34·7	35·7	37·7	32·7
4												
Number of Patients	495	453	466	448	567	508	597	704	736	767	781	740
Person years	1669529	1698970	1722397	1757442	1747899	1757502	1795009	1856049	1914249	1968500	1980367	1990898
Incidence	29·6	26·7	27·1	25·5	32·4	28·9	33·3	37·9	38·4	39·0	39·4	37·2
5 - Most deprived												
Number of Patients	602	603	651	601	648	671	777	863	907	984	1,072	840
Person years	1562361	1578364	1596510	1629715	1614319	1615531	1649633	1698153	1740476	1779274	1776888	1792180
Incidence	38·5	38·2	40·8	36·9	40·1	41·5	47·1	50·8	52·1	55·3	60·3	46·9
Missing												
Number of Patients	39	27	40	49	52	52	57	55	59	68	78	75
Person years	283219	281752	278947	276682	239386	207133	195993	186479	179007	174412	175259	179416
Incidence	13·8	9·6	14·3	17·7	21·7	25·1	29·1	29·5	33·0	39·0	44·5	41·8

Number of Patients = number of patients with a record of alcohol-related liver disease; Person years = total number of person years in the denominator population; IMD = Index of Multiple Deprivation·

STable 13. Annual Incidence of Possible ARLD (per 100,000 person-years)

	2009	2010	2011	2012	2013	2014	2015	2016	2017	2018	2019	2020
**Overall**												
Number of Patients	2,943	2,986	3,180	3,116	3,384	3,453	3,988	4,506	4,978	5,339	5,682	5,103
Person years	8750978	8846664	8922904	9051389	8964563	8937621	9082313	9309892	9501049	9694130	9701055	9659633
Incidence	33·6	33·8	35·6	34·4	37·7	38·6	43·9	48·4	52·4	55·1	58·6	52·8
**Age, years**												
18-29												
Number of Patients	92	126	152	145	170	174	200	223	259	315	356	371
Person years	1716133	1878048	2018381	2163002	2242758	2322856	2433955	2573514	2712668	2853492	2927348	2998143
Incidence	5·4	6·7	7·5	6·7	7·6	7·5	8·2	8·7	9·5	11·0	12·2	12·4
30-39												
Number of Patients	358	319	395	395	433	472	529	638	794	752	862	797
Person years	1612158	1638042	1659860	1697545	1696236	1711787	1760489	1828030	1895596	1964230	1994973	2024012
Incidence	22·2	19·5	23·8	23·3	25·5	27·6	30·0	34·9	41·9	38·3	43·2	39·4
40-49												
Number of Patients	730	749	769	757	843	850	1,021	1,152	1,303	1,402	1,479	1,346
Person years	1722881	1726700	1728043	1742143	1717018	1704180	1725696	1758437	1781289	1803010	1790926	1768868
Incidence	42·4	43·4	44·5	43·5	49·1	49·9	59·2	65·5	73·1	77·8	82·6	76·1
50-59												
Number of Patients	738	742	818	776	924	883	1,101	1,188	1,293	1,429	1,477	1,346
Person years	1319911	1313679	1307240	1309151	1290002	1278284	1291421	1312929	1324534	1335500	1322912	1299313
Incidence	55·9	56·5	62·6	59·3	71·6	69·1	85·3	90·5	97·6	107·0	111·6	103·6
60-69												
Number of Patients	627	643	619	650	665	675	750	901	889	963	1,047	856
Person years	1112479	1096920	1084981	1078150	1056901	1041151	1042403	1048719	1044382	1038298	1013322	975156
Incidence	56·4	58·6	57·1	60·3	62·9	64·8	71·9	85·9	85·1	92·7	103·3	87·8
70-79												
Number of Patients	301	325	344	320	295	345	327	351	380	410	387	331
Person years	755923	732030	708925	686806	651364	620509	600181	583609	560896	537581	506292	467596
Incidence	39·8	44·4	48·5	46·6	45·3	55·6	54·5	60·1	67·7	76·3	76·4	70·8
≥80												
Number of Patients	97	82	83	73	54	55	60	53	61	68	74	56
Person years	511586	461341	415573	374694	310377	258945	228269	204773	181825	162189	145479	126766
Incidence	19·0	17·8	20·0	19·5	17·4	21·2	26·3	25·9	33·5	41·9	50·9	44·2
**Sex**												
Male												
Number of Patients	1,936	1,938	2,055	2,011	2,260	2,189	2,580	2,931	3,217	3,405	3,675	3,111
Person years	4361114	4407871	4439567	4501998	4457738	4459053	4533902	4649837	4752260	4856359	4866397	4844434
Incidence	44·4	44·0	46·3	44·7	50·7	49·1	56·9	63·0	67·7	70·1	75·5	64·2
Female												
Number of Patients	1,007	1,048	1,125	1,105	1,124	1,264	1,408	1,575	1,761	1,934	2,007	1,992
Person years	4389865	4438793	4483337	4549392	4506826	4478569	4548412	4660055	4748789	4837772	4834659	4815200
Incidence	22·9	23·6	25·1	24·3	24·9	28·2	31·0	33·8	37·1	40·0	41·5	41·4
**Ethnicity**												
White												
Number of Patients	1,893	1,996	2,166	2,191	2,452	2,520	2,979	3,404	3,814	4,168	4,493	4,019
Person years	5325187	5544370	5724338	5905135	6019327	6121185	6255993	6436356	6578376	6718120	6733819	6695591
Incidence	35·5	36·0	37·8	37·1	40·7	41·2	47·6	52·9	58·0	62·0	66·7	60·0
Black, African, Caribbean, or Black British												

Number of Patients	91	103	100	126	105	117	105	119	150	159	139	115
Person years	251786	267799	281182	296570	305241	314839	327176	343083	358351	371946	376494	388019
Incidence	36·1	38·5	35·6	42·5	34·4	37·2	32·1	34·7	41·9	42·7	36·9	29·6
South Asian												
Number of Patients	155	167	184	165	192	168	218	249	251	276	287	317
Person years	444180	486847	528581	574296	605516	626818	651590	687472	725623	765759	789932	822042
Incidence	34·9	34·3	34·8	28·7	31·7	26·8	33·5	36·2	34·6	36·0	36·3	38·6
Mixed or Multiple ethnic groups												
Number of Patients	18	16	20	20	15	19	27	26	39	35	38	45
Person years	56717	63407	68764	74414	78975	84355	90241	98138	108050	119342	128807	139861
Incidence	31·7	25·2	29·1	26·9	19·0	22·5	29·9	26·5	36·1	29·3	29·5	32·2
Other ethnicity												
Number of Patients	27	15	32	17	25	22	32	35	41	28	34	35
Person years	66864	73896	80536	87054	91845	96864	102729	110444	118164	126668	132266	139069
Incidence	40·4	20·3	39·7	19·5	27·2	22·7	31·1	31·7	34·7	22·1	25·7	25·2
Missing												
Number of Patients	759	689	678	597	595	608	627	673	684	673	691	572
Person years	2606336	2410441	2239604	2114023	1863753	1693650	1654685	1634519	1612625	1592464	1539933	1475272
Incidence	29·1	28·6	30·3	28·2	31·9	35·9	37·9	41·2	42·4	42·3	44·9	38·8
**Region**												
North East												
Number of Patients	112	125	130	143	162	149	188	187	218	227	262	255
Person years	283190	289061	292695	296353	297589	299126	301704	305986	308856	312649	308019	305880
Incidence	39·5	43·2	44·4	48·3	54·4	49·8	62·3	61·1	70·6	72·6	85·1	83·4
North West												
Number of Patients	711	670	711	726	796	791	892	1,037	1,100	1,240	1,430	1,242
Person years	1656203	1661941	1670384	1681806	1657081	1644519	1666268	1697138	1721074	1746358	1762209	1773082
Incidence	42·9	40·3	42·6	43·2	48·0	48·1	53·5	61·1	63·9	71·0	81·1	70·0
Yorkshire And The Humber												
Number of Patients	85	98	100	86	109	115	132	146	188	178	193	139
Person years	319125	322373	324161	330156	331569	333931	341954	351985	360164	368673	350371	324119
Incidence	26·6	30·4	30·8	26·0	32·9	34·4	38·6	41·5	52·2	48·3	55·1	42·9
East Midlands												
Number of Patients	62	53	81	79	84	88	109	131	118	132	102	108
Person years	211249	215897	218875	223254	223024	222982	227403	234404	239470	248163	231302	215863
Incidence	29·3	24·5	37·0	35·4	37·7	39·5	47·9	55·9	49·3	53·2	44·1	50·0
West Midlands												
Number of Patients	480	458	458	478	557	549	651	764	769	857	891	836
Person years	1398037	1402534	1407515	1426814	1413289	1413152	1446967	1486948	1507265	1533141	1530321	1520477
Incidence	34·3	32·7	32·5	33·5	39·4	38·8	45·0	51·4	51·0	55·9	58·2	55·0
East of England												
Number of Patients	88	113	116	97	109	110	135	152	143	205	184	162
Person years	387038	391685	395301	402795	405241	406423	411885	421291	426776	425889	410705	379143
Incidence	22·7	28·8	29·3	24·1	26·9	27·1	32·8	36·1	33·5	48·1	44·8	42·7
South West												
Number of Patients	556	550	624	592	610	629	697	741	910	873	868	744
Person years	1549343	1587076	1611901	1652816	1643615	1656357	1701277	1778994	1860003	1931353	1970822	2014948
Incidence	35·9	34·7	38·7	35·8	37·1	38·0	41·0	41·7	48·9	45·2	44·0	36·9
South East												
Number of Patients	483	491	554	537	571	576	730	822	917	980	1,054	986
Person years	1806837	1824398	1839352	1861568	1852427	1849859	1871331	1904910	1928846	1962281	1979257	1970825

Incidence	26·7	26·9	30·1	28·8	30·8	31·1	39·0	43·2	47·5	49·9	53·3	50·0
London												
Number of Patients	357	414	389	360	374	437	436	507	587	619	661	579
Person years	1099876	1111251	1122095	1135036	1099964	1070292	1071898	1085686	1105205	1121461	1114178	1109623
Incidence	32·5	37·3	34·7	31·7	34·0	40·8	40·7	46·7	53·1	55·2	59·3	52·2
Northern Ireland												
Number of Patients	6	12	16	14	12	8	18	19	27	28	37	52
Person years	36417	37115	37709	38401	38903	39589	40331	41243	42091	42998	43787	45199
Incidence	16·5	32·3	42·4	36·5	30·8	20·2	44·6	46·1	64·1	65·1	84·5	115·0
Missing												
Number of Patients	3	2	1	4	0	1	0	0	1	0	0	0
Person years	3663	3333	2915	2390	1860	1393	1296	1309	1298	1166	85	474
Incidence	81·9	60·0	34·3	167·4	0	71·8	0	0	77·0	0	0	0
**IMD quintile**												
1 - Least deprived												
Number of Patients	387	403	462	446	493	482	578	680	732	817	885	784
Person years	1808703	1821981	1830206	1847602	1839112	1833278	1857969	1896348	1921344	1947371	1943269	1920831
Incidence	21·4	22·1	25·2	24·1	26·8	26·3	31·1	35·9	38·1	42·0	45·5	40·8
2												
Number of Patients	451	507	499	508	474	572	679	722	844	886	972	895
Person years	1777959	1795018	1809028	1829303	1819351	1816479	1845168	1886391	1918788	1955995	1950956	1919466
Incidence	25·4	28·2	27·6	27·8	26·1	31·5	36·8	38·3	44·0	45·3	49·8	46·6
3												
Number of Patients	506	522	556	534	616	603	713	795	949	964	1,073	921
Person years	1652211	1673787	1689217	1714293	1708335	1711736	1742852	1791089	1832118	1873922	1879933	1862795
Incidence	30·6	31·2	32·9	31·1	36·1	35·2	40·9	44·4	51·8	51·4	57·1	49·4
4												
Number of Patients	704	651	699	674	798	756	879	984	1,060	1,182	1,146	1,093
Person years	1668184	1697543	1720887	1755851	1746231	1755729	1793099	1854011	1912084	1966137	1977869	1988296
Incidence	42·2	38·3	40·6	38·4	45·7	43·1	49·0	53·1	55·4	60·1	57·9	55·0
5 - Most deprived												
Number of Patients	840	856	895	883	932	970	1,064	1,246	1,304	1,396	1,480	1,279
Person years	1560916	1576815	1594867	1627923	1612408	1613510	1647491	1695850	1738015	1776649	1774174	1789294
Incidence	53·8	54·3	56·1	54·2	57·8	60·1	64·6	73·5	75·0	78·6	83·4	71·5
Missing												
Number of Patients	55	47	69	71	71	71	75	79	90	94	126	131
Person years	283098	281616	278800	276521	239221	206979	195836	186321	178840	174226	175051	179170
Incidence	19·4	16·7	24·7	25·7	29·7	34·3	38·3	42·4	50·3	54·0	72·0	73·1

Number of Patients = number of patients with a record of alcohol-related liver disease; Person years = total number of person years in the denominator population; IMD = Index of Multiple Deprivation·

STable 14. Crude hazard ratios for all-cause mortality in people with ARLD compared to those without, stratified by age, sex, ethnicity, region and index of multiple deprivation (IMD) quintile

	Definite ARLD				Probable ARLD				Possible ARLD			
	Number of patients	Person years	IR	Crude HR (95%CI)	Number of patients	Person years	IR	Crude HR (95%CI)	Number of patients	Person years	IR	Crude HR (95%CI)
**Overall**	16809	216598	77·6	5·56 (5·43, 5·68)	22089	270356	81·70	5·31 (5·21, 5·41)	29335	375450	78·13	4·41 (4·34, 4·48)
**Age, years**												
18-29	105	3312	31·70	39·13 (21·54, 71·12)	131	4130	31·72	40·46 (23·32, 70·21)	206	12768	16·13	20·64 (14·71, 20·79)
30-39	916	19268	47·54	26·48 (22·39, 31·33)	1103	23192	47·56	25·00 (21·51, 29·04)	1221	36349	33·59	23·35 (20·26, 26·92)
40-49	3218	54357	59·20	16·95 (15·75, 18·24)	3988	66682	59·81	16·65 (15·59, 17·78)	4430	86267	51·35	14·20 (13·38, 15·07)
50-59	4960	69173	71·70	8·87 (8·47, 9·30)	6282	85468	73·50	8·41 (8·07, 8·76)	7242	106967	67·70	7·96 (7·67, 8·26)
60-69	4714	50698	92·98	4·86 (4·67, 5·06)	6219	63993	97·18	4·76 (4·60, 4·92)	7954	85021	93·55	4·51 (4·38, 4·65)
≥70	2896	19789	146·34	2·68 (2·57, 2·80)	4366	26892	162·36	2·85 (2·75, 2·95)	8282	48078	172·26	2·73 (2·66, 2·80)
**Sex**												
Male	11591	153031	75·70	4·99 (4·86, 5·12)	15590	192989	80·78	4·79 (4·69, 4·90)	19267	245155	78·59	4·25 (4·16, 4·33)
Female	5218	63563	82·09	7·33 (7·02, 7·64)	6499	77364	84·01	7·04 (6·78, 7·31)	10068	130291	77·27	4·74 (4·62, 4·88)
**Ethnicity**												
White	11869	171259	69·30	5·64 (5·50, 5·79)	15757	214424	73·49	5·39 (5·27, 5·51)	20366	279218	72·94	4·56 (4·47, 4·65)
Black, African, Caribbean, or Black British	198	3663	54·05	4·46 (3·66, 5·44)	256	4609	55·55	3·78 (3·20, 4·47)	472	13199	35·76	2·99 (2·65, 3·36)
South Asian	356	6547	54·38	5·37 (4·61, 6·25)	461	8009	57·56	5·40 (4·72, 6·18)	974	20892	46·62	4·15 (3·80, 4·53)
Mixed or Multiple ethnic groups	31	918	33·79	4·49 (2·71, 7·46)	53	1165	45·49	6·15 (4·03, 9·40)	87	2543	34·21	4·38 (3·25, 5·91)
Other ethnicity	62	1221	5·78	7·52 (5·03, 11·22)	84	1512	55·56	6·19 (4·48, 8·57)	137	3002	45·63	5·09 (3·99, 6·49)
Missing	4293	32990	130·13	5·50 (5·27, 5·74)	5478	40638	134·80	5·27 (5·07, 5·47)	7299	56594	128·97	4·25 (4·12, 4·39)
**Region**												
London	2345	37078	63·25	4·56 (4·30, 4·82)	3110	46255	67·24	4·63 (4·41, 4·87)	4338	72041	60·22	3·74 (3·59, 3·90)
East Midlands	394	4925	80·00	5·94 (5·14, 6·86)	495	5893	84·00	6·12 (5·38, 6·96)	666	7894	84·37	5·27 (4·73, 5·87)
East of England	512	6493	78·85	6·23 (5·48, 7·08)	687	8199	83·79	6·06 (5·43, 6·76)	989	11851	83·45	4·91 (4·51, 5·36)
North East	943	11700	80·60	5·65 (5·16, 6·20)	1212	14383	84·27	5·18 (4·79, 5·61)	1418	17145	82·71	4·54 (4·23, 4·87)
North West	4369	50584	86·37	5·63 (5·39, 5·88)	5786	64147	90·20	5·30 (5·11, 5·50)	7253	83429	86·94	4·60 (4·45, 4·74)
South East	2670	34844	76·56	5·74 (5·43, 6·07)	3604	43892	82·11	5·27 (5·03, 5·52)	5070	62463	81·17	4·30 (4·17, 4·47)
South West	1989	26157	76·04	6·00 (5·62, 6·40)	2607	32710	79·70	5·84 (5·52, 6·17)	3537	46072	76·77	4·62 (4·41, 4·84)

West Midlands	2893	35587	81·29	5·84 (5·54, 6·16)	3690	43551	84·73	5·57 (5·32, 5·84)	4891	59368	82·38	4·60 (4·42, 4·78)
Yorkshire And The Humber	610	8009	76·17	5·44 (4·86, 6·09)	797	9840	81·00	5·27 (4·78, 5·81)	1033	13127	78·69	4·63 (4·25, 5·03)
Northern Ireland	75	1118	67·08	5·55 (5·43, 5·68)	96	1405	68·33	5·31 (5·21, 5·41)	125	1935	64·60	4·41 (4·34, 4·48)
Missing	9	70	127·99	2·61 (1·16, 5·87)	10	80	124·44	2·46 (1·14, 5·32)	15	124	120·51	2·22 (1·19, 4·14)
**IMD quintile**												
1 - Least deprived	2136	28162	75·85	6·78 (6·40, 7·17)	2942	35830	82·11	6·63 (6·32, 6·96)	4149	52100	79·64	5·28 (5·08, 5·50)
2	2487	34855	71·35	5·78 (5·48, 6·10)	3363	43684	76·98	5·61 (5·36, 5·87)	4680	61369	76·26	4·63 (4·46, 4·81)
3	2771	38103	72·72	5·27 (5·00, 5·55)	3685	47361	77·81	5·29 (5·06, 5·54)	5097	66497	76·65	4·47 (4·31, 4·64)
4	3774	47878	78·82	5·18 (4·93, 5·43)	4916	60021	81·91	4·81 (4·62, 5·02)	6400	83575	76·58	4·05 (3·91, 4·19)
5 - Most deprived	5381	63177	85·17	4·55 (4·36, 4·75)	6852	78102	87·73	4·24 (4·09, 4·40)	8564	104527	81·93	3·67 (3·56, 3·79)
Missing	260	4422	58·80	5·88 (4·92, 7·01)	331	5358	61·78	5·59 (4·78, 6·53)	445	7381	60·29	4·46 (3·93, 5·06)

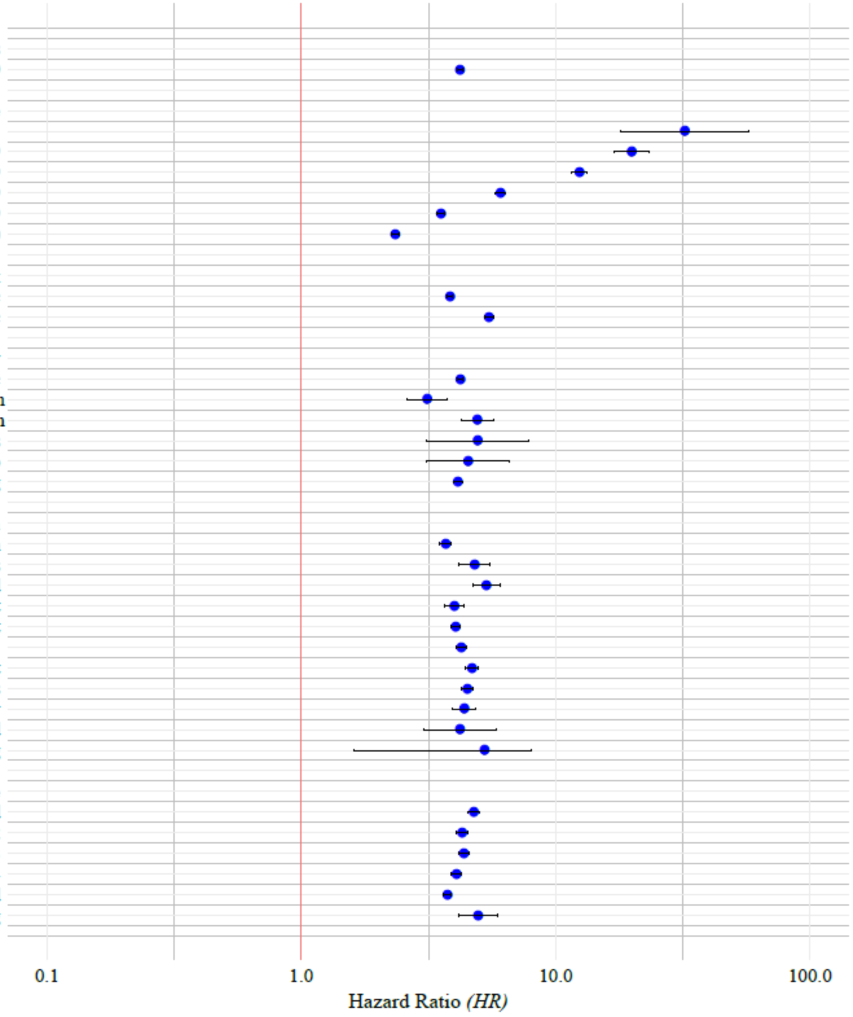

	Events/Patients: Patients with Probable ARLD	Events/Patients: Matched patients with no liver disease (unexposed)	HR	95% CI
**Overall analysis**				
Probable ARLD	22,089/66,066	20,939/264,249	4.2	4.12–4.29
**Stratified by Age**				
18–29	131/1,080	14/4,404	32.13	18.00–57.36
30–39	1,103/5,825	202/23,491	19.89	16.90–23.42
40–49	3,988/15,218	1,148/60,566	12.4	11.54–13.33
50–59	6,282/19,880	3,696/79,538	6.06	5.80–6.34
60–69	6,219/15,721	6,945/62,844	3.54	3.41–3.67
>=70	4,366/8,342	8,934/33,406	2.34	2.25–2.43
**Stratified by Sex**				
Male	15,590/46,932	16,070/187,719	3.84	3.75–3.94
Female	6,499/19,133	4,869/76,530	5.47	5.25–5.70
**Stratified by Ethnicity**				
White	15,757/50,982	14,820/203,916	4.22	4.12–4.33
Black, African, Caribbean or Black British	256/1,116	294/4,461	3.12	2.60–3.74
South Asian	461/1,998	395/7,992	4.92	4.26–5.68
Mixed or Multiple ethnic groups	53/300	36/1,200	4.93	3.10–7.84
Other ethnic group	84/386	65/1,544	4.52	3.11–6.59
Missing	5,478/11,284	5,329/45,136	4.13	3.97–4.30
**Stratified by Region**				
London	3,110/11,234	3,079/44,936	3.69	3.50–3.90
East Midlands	495/1,475	442/5,900	4.8	4.17–5.53
East of England	687/2,069	615/8,276	5.34	4.74–6.02
North East	1,212/3,312	1,240/13,248	3.99	3.67–4.34
North West	5,786/15,896	5,571/63,584	4.04	3.88–4.20
South East	3,604/10,894	3,382/43,576	4.26	4.05–4.48
South West	2,607/8,038	2,315/32,148	4.69	4.41–4.98
West Midlands	3,690/10,534	3,377/42,136	4.5	4.28–4.73
Yorkshire And The Humber	797/2,302	806/9,208	4.37	3.93–4.85
Northern Ireland	91/277	96/1,105	4.2	3.02–5.84
Missing	10/35	19/132	5.25	1.61–17.13
**Stratified by IMD quintile**				
1 Least deprived	2,942/8,687	3,635/54,188	4.76	4.52–5.02
2	3,363/10,300	4,034/55,403	4.28	4.07–4.50
3	3,685/11,511	3,826/50,371	4.36	4.15–4.57
4	4,916/14,778	4,248/50,158	4.07	3.89–4.25
5 Most deprived	6,852/19,380	4,890/47,831	3.75	3.61–3.90
Missing	331/1,410	306/6,298	4.95	4.16–5.90

14.72 x 8.35 in

SFigure 7. Adjusted hazard ratios for all-cause mortality in people with Probable ARLD compared to those without liver disease, stratified by age, sex, ethnicity, region and IMD quintile

ARLD = alcohol related liver disease; IMD = Index of Multiple Deprivation; HR = hazard ratio; CI = confidence interval

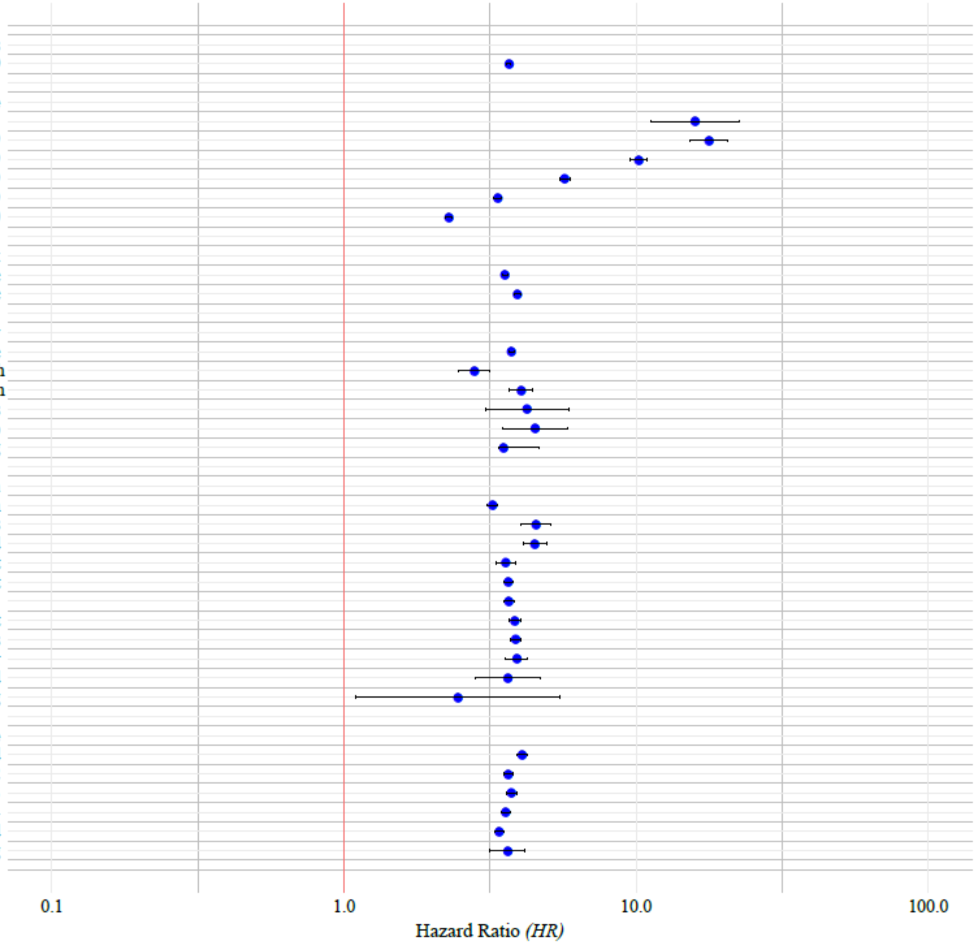

	Events/Patients: Patients with Possible ARLD	Events/Patients: Matched patients with no liver disease (unexposed)	HR	95% CI
**Overall analysis**				
Possible ARLD	29,335/91,091	32,263/364,338	3.67	3.61–3.73
**Stratified by Age**				
18–29	206/3,104	40/12,484	15.88	11.21–22.49
30–39	1,221/8,683	226/34,980	17.76	15.29–20.63
40–49	4,430/18,912	1,436/75,283	10.19	9.56–10.87
50–59	7,242/24,443	4,367/97,908	5.69	5.46–5.92
60–69	7,954/20,574	9,042/82,216	3.36	3.25–3.47
>=70	8,282/15,375	17,152/61,467	2.28	2.22–2.34
**Stratified by Sex**				
Male	19,267/59,607	21,784/238,413	3.55	3.4–3.62
Female	10,068/31,483	10,479/125,925	3.92	3.81–4.03
**Stratified by Ethnicity**				
White	20,366/66,262	22,018/265,032	3.73	3.66–3.81
Black, African, Caribbean or Black British	472/3,001	643/12,001	2.79	2.47–3.15
South Asian	974/4,900	1,026/19,600	4.04	3.69–4.43
Mixed or Multiple ethnic groups	87/660	85/2,637	4.23	3.05–5.97
Other ethnic group	137/739	125/2,952	4.51	3.48–5.85
Missing	7,299/15,529	8,366/62,116	3.51	3.40–3.63
**Stratified by Region**				
London	4,338/17,214	5,062/68,856	3.22	3.09–3.36
East Midlands	666/2,021	665/8,083	4.54	4.04–5.10
East of England	989/2,970	1,064/11,880	4.49	4.10–4.93
North East	1,418/3,899	1,630/15,590	3.57	3.31–3.85
North West	7,253/20,436	7,803/81,744	3.65	3.53–3.77
South East	5,070/15,491	5,662/61,964	3.66	3.52–3.81
South West	3,537/11,161	3,780/44,640	3.84	3.66–4.04
West Midlands	4,891/14,335	5,253/57,340	3.86	3.71–4.02
Yorkshire And The Humber	1,033/3,096	1,152/12,384	3.9	3.57–4.26
Northern Ireland	125/416	163/1,661	3.64	2.82–4.70
Missing	15/52	29/196	2.45	1.10–5.46
**Stratified by IMD quintile**				
1 Least deprived	4,149/12,392	5,759/73,157	4.07	3.90–4.24
2	4,680/14,524	6,386/75,371	3.65	3.51–3.80
3	5,097/16,133	5,943/69,451	3.74	3.59–3.89
4	6,400/20,348	6,500/70,817	3.57	3.45–3.71
5 Most deprived	8,564/25,710	7,159/66,622	3.39	3.28–3.51
Missing	445/1,984	516/8,920	3.63	3.16–4.17

SFigure 8. Adjusted hazard ratios for all-cause mortality in people with Possible ARLD compared to those without liver disease, stratified by age, sex, ethnicity, region and IMD quintile

ARLD = alcohol related liver disease; IMD = Index of Multiple Deprivation; HR = hazard ratio; CI = confidence interval

STable 15. Baseline characteristics of patients included in the ARLD-related mortality and hospitalisation cohort analyses

	**Definite ARLD analysis cohort** (n=50 409)	**Probable ARLD analysis cohort** (n=64681)	**Possible ARLD analysis cohort** (n=89097)
Age, years			
Mean (SD)	54·9 (12·1)	55·6 (12·3)	56·1 (14·1)
18–29	835 (1·7%)	1045 (1·6%)	3020 (3·4%)
30–39	4674 (9·3%)	5684 (8·8%)	8447 (9·5%)
40–49	12110 (24·0%)	14875 (23.0%)	18458 (20·7%)
50–59	15476 (30·7%)	19467 (30·1%)	23924 (26·9%)
60–69	11776 (23·3%)	15411 (23·8%)	20154 (22·6%)
≥70††	5538 (11·0%)	8199 (12·7%)	15094 (16·9%)
Sex*			
Male	35398 (7·2 %)	45962 (71·1%)	58340 (65·5%)
Female	15010 (29·8%)	18718 (28·9%)	30756 (34·5%)
Ethnicity			
White	38814 (77·0%)	50035 (77·4%)	65011 (73·0%)
Black, African, Caribbean, or Black British	850 (1·7%)	1102 (1·7%)	2950 (3·3%)
South Asian	1587 (3·2%)	1972 (3·1%)	4809 (5·4%)
Mixed or multiple ethnic groups	224 (0·4%)	298 (0.5%)	649 (0·7%)
Other ethnicity	303 (0·6%)	383 (0·6%)	730 (0·8%)
Missing data	8631 (17·1%)	10891 (16·8%)	14948 (16.8%)
Geographical region			
North East	2573 (5·1%)	3295 (5·1%)	3876 (4·4%)
North West	11956 (23·7%)	15583 (24.1%)	20015 (22·5%)
Yorkshire and The Humber	1771 (3·5%)	2262 (3·5%)	3041 (3·4%)
East Midlands	1139 (2·3%)	1429 (2·2%)	1961 (2·2%)
West Midlands	8330 (16.5%)	10428 (16·1%)	14173 (15·9%)
East of England	1564 (3·1%)	2041 (3·1%)	2930 (3.3%)
South West	6137 (12·2%)	7872 (12·2%)	10930 (12·3%)
South East	8197 (16·2%)	10659 (16·5%)	15164 (17·0%)
London	8713 (17·3%)	11077 (17·1%)	16956 (19.0%)
Missing data	29 (0·1%)	35 (0·1%)	51 (0·1%)
IMD quintile			
1 (least deprived)	6609 (13.1%)	8677 (13.4%)	12367 (13·9%)
2	7925 (15·7%)	10298 (15·9%)	14508 (16.3%)
3	9016 (17·9%)	11502 (17·8%)	16112 (18·1%)
4	11482 (22·8%)	14757 (22·8%)	20319 (22·8%)
5 (most deprived)	15291 (30·3%)	19352 (29·9%)	25668 (28·8%)
Missing data	86 (0.2%)	103 (0·2%)	123 (0·1%)
Smoking status			
Never smoked	6981 (13·9%)	8934 (13·5%)	16508 (18·5%)
Ex-smoker	10234 (20·3%)	13916 (21.5%)	22148 (24·9%)

Current smoker	32335 (64·1%)	40988 (63.4%)	48702 (54·7%)
Missing data	859 (1·7%)	1043 (1·6%)	1739 (2·0%)
BMI, kg/m²			
<18·5	1856 (3·7%)	2294 (3·6%)	2894 (3·3%)
18·5to <25	15034 (29·8%)	19248 (29·8%)	25936 (29·1%)
25 to <30	14568 (28·9%)	18852 (29·2%)	25471 (28·6%)
30 to <35	8250 (16·4%)	10747 (16·6%)	14882 (16·7%)
35 to <40	3052 (6·0%)	4034 (6·2%)	5989 (6·7%)
≥40	1276 (2·5%)	1772 (2.7%)	2969 (3·3%)
Missing data	6373 (12·6%)	7734 (12·0%)	10956 (12·3%)
Alcohol misuse	50409 (100·0%)	64681 (100·0%)	63215 (71·0%)
Excess alcohol consumption, units per week			
14–28	11801 (23·4%)	17295 (26·7%)	16912 (19·0%)
>28	18924 (37·5%)	23988 (37·1%)	23732 (26·6%)
Medical and psychiatric comorbidities			
Cardiovascular disease	7293 (14·5%)	10082 (15·6%)	14777 (16·6%)
Hypertension	17539 (34·8%)	23218 (35·9%)	32095 (36·0%)
Type 1 diabetes	499 (1·0%)	733 (1·1%)	1247 (1·4%)
Type 2 diabetes	7233 (14·4%)	10458 (16·2%)	17201 (19·3%)
Chronic kidney disease	1875 (3·7%)	2875 (4·4%)	5826 (6·5%)
Cancer	2377 (4·7%)	3565 (5·5%)	5947 (6.7%)
COPD	4915 (9·8%)	6407 (9·9%)	8061 (9·1%)
Asthma	9636 (19·1%)	12308 (19·0%)	16573 (18·6%)
Dementia	853 (1·7%)	1108 (1·7%)	1634 (1·8%)
Anxiety	15290 (30·3%)	18966 (29·3%)	22641 (25·4%)
Depression	20204 (40·1%)	25305 (39·1%)	30836 (34·6%)
Severe mental illness†	2114 (4·2%)	2766 (4·3%)	3214 (3·6%)

Data are n (%) except where otherwise specified. ARLD=alcohol-related liver disease. COPD=chronic obstructive pulmonary disease. IMD=Index of Multiple Deprivation. *The number of patients with ARLD stratified by sex adds up to one less that the total as one patient was coded as intersex. †Severe mental illness: schizophrenia, bipolar disorder, psychosis, paranoid ideation, manic disorders or delusional disorders. ††Patients aged 70–79 years and those aged ⩾80 years were combined into a single ⩾70 years age group in the mortality analysis due to the limited number of patients in these two groups.

STable 16. Crude hazard ratios for ARLD-related mortality among people with ARLD, stratified by age, sex, ethnicity, region and index of multiple deprivation (IMD) quintile

	Definite ARLD				Probable ARLD				Possible ARLD			
	Number of patients	Person years	IR	Crude HR (95% CI)	Number of patients	Person years	IR	Crude HR (95% CI)	Number of patients	Person years	IR	Crude HR (95% CI)
**Age, years**												
18-29	83	3233	25·68	1	100	4031	24·81	1	140	12503	11.20	1
30-39	664	18914	35.10	1·40(1·11, 1·75)	928	22732	40·82	1·67 (1·36, 2·05)	1019	35555	28·66	2·59 (2·17, 3·09)
40-49	2068	53196	38·88	1·58 (1·27, 1·97)	3056	65274	46.81	1·96 (1·61, 2·39)	3465	84426	41.04	3·80 (3·21, 4·50)
50-59	2482	67734	36·64	1·48 (1·20, 1·85)	3940	83715	47.06	1·95 (1·60, 2·38)	4763	104801	45.45	4·15 (3·51, 4·91)
60-69	1724	49705	34·68	1·38 (1·11, 1·73)	3092	62790	49.24	2·01 (1·65, 2·45)	4316	83444	51.72	4·64 (3·92, 5·49)
≥70	597	19416	30·74	1·15 (0·92, 1·45)	1501	26444	56·76	2·16 (1·77, 2·65)	3227	47131	68.47	5.68 (4·80, 6·73)
**Sex**												
Male	5046	149905	33·66	1	8669	189171	45.83	1	11080	240331	46.10	1
Female	2572	62289	41.29	1·22(1·16, 1·28)	3948	75810	52.08	1·13 (1·09, 1·17)	5850	127525	45.87	1·00 (0·97, 1·03)
**Ethnicity**												
White	5214	168312	30·98	1	8756	210846	41.53	1	11588	274515	42.21	1
Black, African, Caribbean, or Black British	62	3639	17.03	0·55 (0·43, 0·71)	122	4571	26·69	0·64 (0·54, 0·77)	211	13027	16.20	0·39 (0·34, 0·44)
South Asian	161	6494	24·79	0·79 (0·68, 0·92)	266	7907	33·64	0·80 (0·71, 0·91)	524	20569	25·47	0·60 (0·55, 0·66)
Mixed or Multiple ethnic groups	17	916	18·56	0·59 (0·37, 0·96)	27	1163	23·21	0·55 (0·38, 0·81)	51	2520	20·24	0·47 (0·36, 0·62)
Other ethnicity	24	1214	19·76	0·63 (0·42, 0·93)	43	1501	28·64	0·68 (0·51, 0·92)	60	2972	20.19	0·47 (0·37, 0·61)
Missing	2140	31623	67.67	2·09 (1.99, 2·20)	3403	38997	87.26	2.02 (1·94, 2·10)	4496	54256	82.87	1·89 (1·83, 1·96)
**Region**												
London	891	36704	24·28	1	1681	45765	36.73	1	2386	71211	32.19	1
East Midlands	178	4812	36·99	1·52 (1·30, 1·79)	298	5757	51·76	1·40 (1·24, 1·58)	387	7711	50.18	1·47 (1·32, 1·64)

East of England	238	6408	37.14	1·51 (1·31, 1·75)	424	8096	52.37	1·42 (1·27, 1·57)	612	11712	52.25	1·55 (1·42, 1·69)
North East	433	11657	37·14	1·58 (1·41, 1·78)	702	14338	48·96	1·37 (1·25, 1·50)	843	17086	46.12	1·51 (1·40, 1·63)
North West	2012	49751	40.44	1·68 (1·55, 1·81)	3287	63090	52.10	1·42 (1·34, 1·50)	4203	81945	49.76	1·54 (1·46, 1·62)
South East	1158	34118	33·94	1·40 (1·28, 1·52)	2060	42984	47.92	1·30 (1·22, 1·39)	2912	61235	45.85	1·42 (1·34, 1·49)
South West	986	25603	38.51	1·59 (1·46, 1·75)	1576	32085	49.12	1·34 (1·25, 1·43)	2052	45227	43.45	1·36 (1·28, 1·44)
West Midlands	1470	35233	41·72	1·73 (1·60, 1·89)	2187	43140	50·70	1·39 (1·31, 1·49)	2990	58760	49.09	1·53 (1·45, 1·61)
Yorkshire And The Humber	249	7841	31·76	1·33 (1·16, 1·53)	393	9649	40.73	1·12 (0·99, 1·25)	536	12854	38.31	1·26 (1·14, 1·38)
Missing	3	70	42.66	1·53 (0·49, 4.76)	9	80	112.00	2.66 (1·38, 5·12)	9	119	39.36	1.96 (1·02, 3·78)
**IMD quintile**												
1 - Least deprived	967	28128	34·38	1	1641	35787	45·85	1	2320	51981	44·63	1
2	1123	34818	32·25	0·95 (0·87, 1·03)	1932	43643	44·26	0·97 (0·91, 1·04)	2703	61307	44·09	0·99 (0·94, 1·05)
3	1237	38068	32·49	0·94 (0·86, 1·02)	2117	47322	44·74	0·97 (0·91, 1·04)	2925	66404	44·05	0·98 (0·93, 1·03)
4	1690	47815	35·34	1·02 (0·94, 1·11)	2850	59933	47·55	1·03 (1·06, 1·19)	3764	83453	45·10	1·00 (0·95, 1·05)
5 - Most deprived	2581	63075	40·91	1·18 (1·10, 1·27)	4043	77979	51·85	1·12 (1·06, 1·19)	5179	104341	49·63	1·10 (1·05, 1·15)
Missing	20	294	68.12	1.88 (1.21, 2.92)	34	321	105.9	2.16 (1.54, 3.04)	39	374	104.28	2.15 (1.56, 2.94)

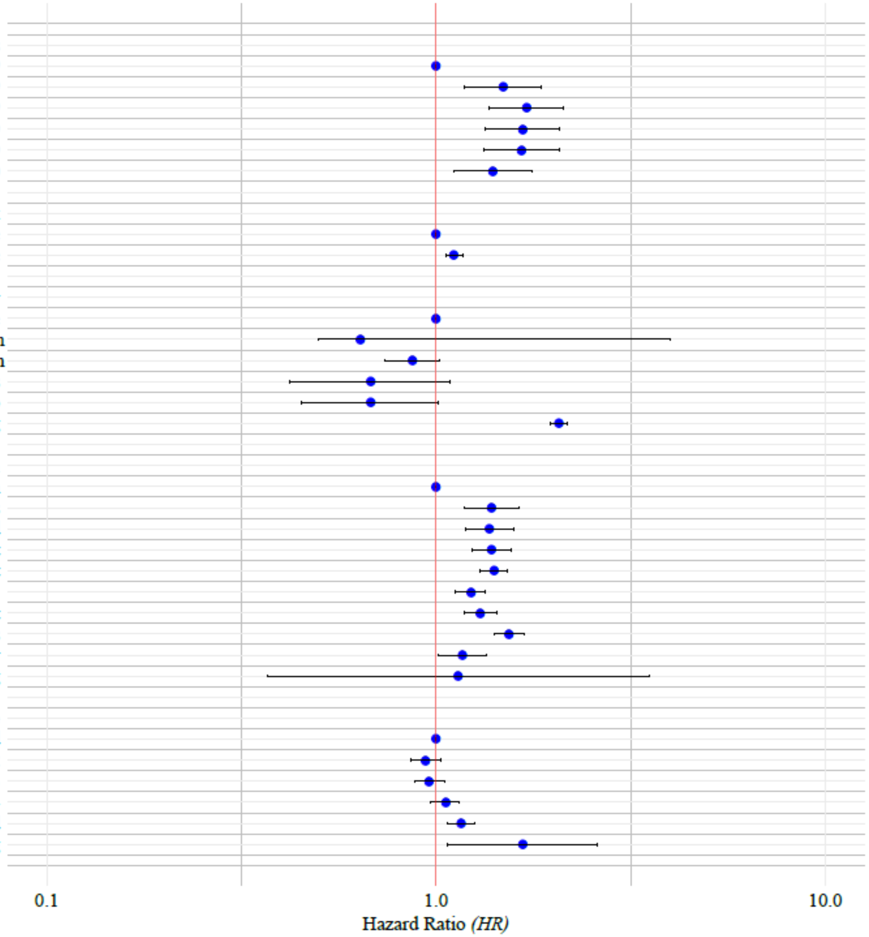

	Events/Patients	HR	95% CI
**Age, years**			
18–29	83/835	1	Ref
30–39	664/4,674	1.49	1.18–1.87
40–49	2,068/12,110	1.71	1.37–2.13
50–59	2,482/15,476	1.67	1.34–2.07
60–69	1,724/11,776	1.66	1.33–2.08
>=70	597/5,538	1.4	1.11–1.77
**Sex**			
Male	5,046/35,398	1	Ref
Female	2,572/15,010	1.11	1.06–1.17
**Ethnicity**			
White	5,214/38,814	1	Ref
Black, African, Caribbean or Black British	62/850	0.64	0.50–8.28
South Asian	161/1,587	0.87	0.74–1.02
Mixed or Multiple ethnic groups	17/224	0.68	0.42–1.09
Other ethnic group	24/303	0.68	0.45–1.01
Missing	2,140/8,631	2.07	1.97–2.18
**Region**			
London	891/8,713	1	Ref
East Midlands	178/1,139	1.39	1.18–1.63
East of England	238/1,564	1.37	1.19–1.59
North East	433/2,573	1.39	1.24–1.56
North West	2,012/11,956	1.41	1.30–1.53
South East	1,158/8,197	1.23	1.12–1.34
South West	986/6137	1.3	1.18–1.43
West Midlands	1,470/8,330	1.54	1.41–1.68
Yorkshire And The Humber	249/1,771	1.17	1.01–1.35
Missing	3/29	1.14	0.37–3.54
**IMD quintile**			
1 Least deprived	967/6,609	1	Ref
2	1,123/7,925	0.94	0.86–1.03
3	1,237/9,016	0.96	0.88–1.05
4	1,690/11,482	1.06	0.97–1.15
5 Most deprived	2,581/15,291	1.16	1.07–1.26
Missing	20/86	1.67	1.07–2.60

SFigure 9. Adjusted hazard ratios for ARLD-related mortality among patients with Definite ARLD by age, sex, ethnicity, region and IMD quintile

ARLD = alcohol related liver disease; IMD = Index of Multiple Deprivation; HR = hazard ratio; CI = confidence interval; Ref = reference. Missing categories were included in the regression analyses to minimise selection bias; however, HRs for missing categories should not be interpreted since many have small numbers and since it is not possible to know the characteristic of patients included in this group.

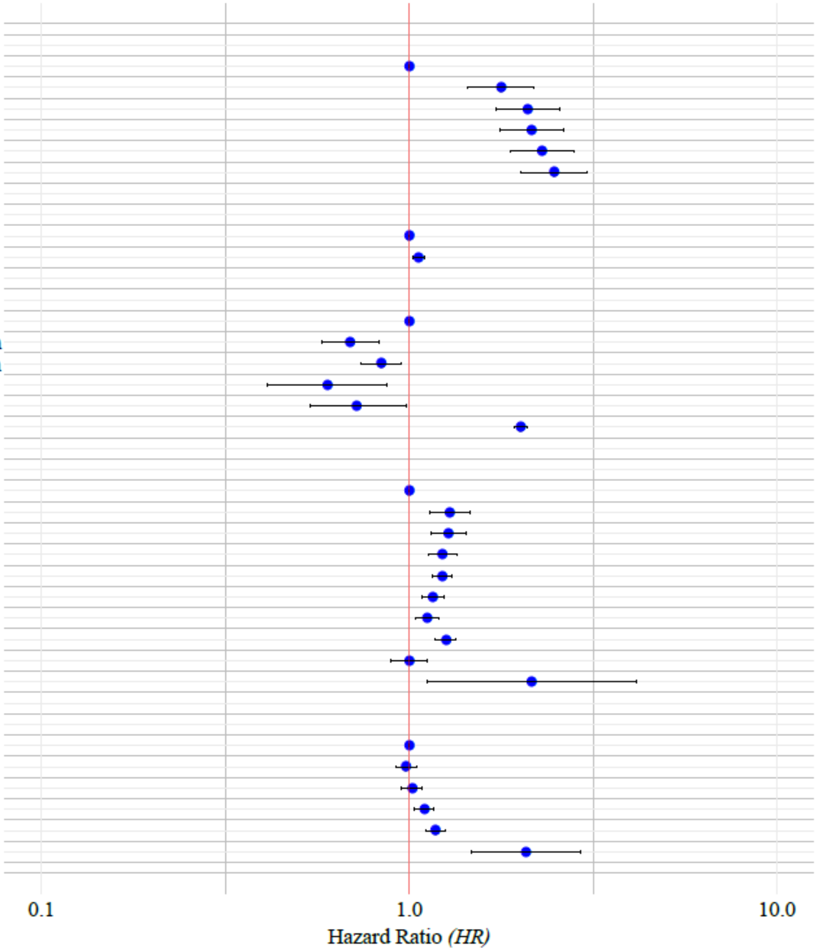

	Events/Patients	HR	95% CI
**Age, years**			
18–29	100/1,045	1	Ref
30–39	928/5,684	1.78	1.44, 2.18
40–49	3056/14,875	2.1	1.72, 2.56
50–59	3940/19,467	2.15	1.76, 2.63
60–69	3092/15,411	2.3	1.88, 2.81
>=70	1501/8,199	2.48	2.02, 3.04
**Sex**			
Male	8,669/45,962	1	Ref
Female	3,948/18,718	1.06	1.02, 1.10
**Ethnicity**			
White	8,756/50,035	1	Ref
Black, African, Caribbean or Black British	122/1,102	0.69	0.58, 0.83
South Asian	266/1,972	0.84	0.74, 0.95
Mixed or Multiple ethnic groups	27/298	0.6	0.41, 0.87
Other ethnic group	43/383	0.72	0.54, 0.98
Missing	3,403/10,891	2.01	1.93, 2.09
**Region**			
London	1,681/11,077	1	Ref
East Midlands	298/1,429	1.29	1.14, 1.46
East of England	424/2,041	1.28	1.15, 1.43
North East	702/3,295	1.23	1.13, 1.35
North West	3,287/15,583	1.23	1.16, 1.31
South East	2,060/10,659	1.16	1.08, 1.24
South West	1,576/7,872	1.12	1.04, 1.20
West Midlands	2,187/10,428	1.26	1.18, 1.34
Yorkshire And The Humber	393/2,262	1	0.89, 1.12
Missing	9/35	2.15	1.12, 4.14
**IMD quintile**			
1 Least deprived	1,641/8,677	1	Ref
2	1,932/10,290	0.98	0.92, 1.05
3	2,117/11,502	1.02	0.95, 1.08
4	2,850/14,757	1.1	1.03, 1.17
5 Most deprived	4,043/19,352	1.18	1.11, 1.25
Missing	34/103	2.08	1.48, 2.92

SFigure 10. Adjusted hazard ratios for ARLD-related mortality among patients with Probable ARLD by age, sex, ethnicity, region and IMD quintile

ARLD = alcohol related liver disease; IMD = Index of Multiple Deprivation; HR = hazard ratio; CI = confidence interval; Ref = reference. Missing categories were included in the regression analyses to minimise selection bias; however, HRs for missing categories should not be interpreted since many have small numbers and since it is not possible to know the characteristic of patients included in this group.

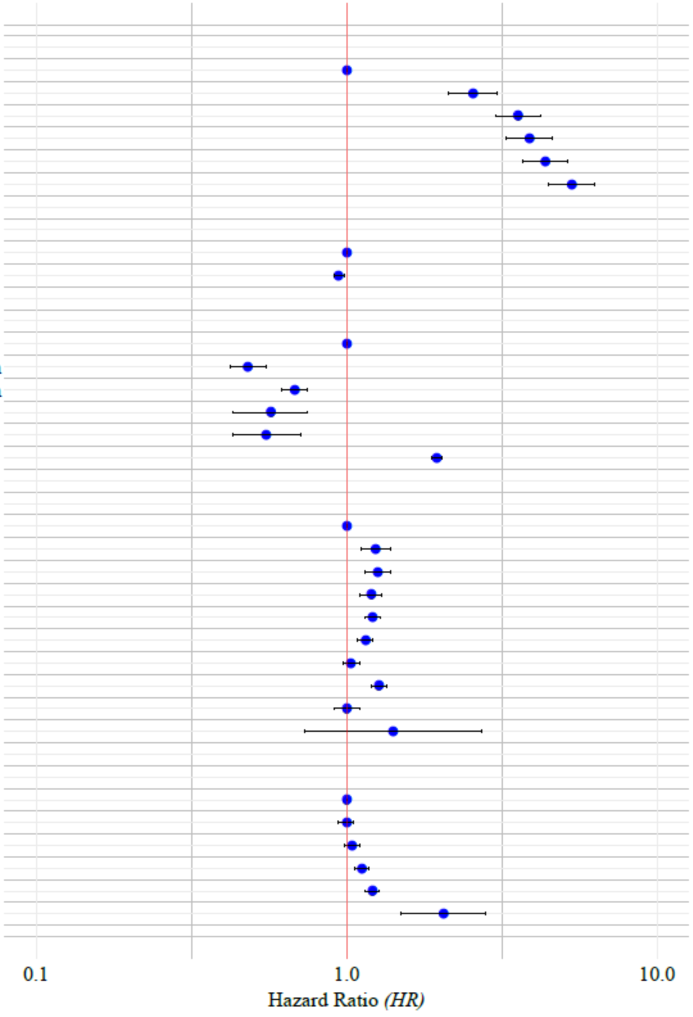

	Events/Patients	HR	95% CI
**Age, years**			
18–29	140/3,020	1	Ref
30–39	1,019/8,447	2.55	2.13, 3.04
40–49	3,465/18,458	3.56	3.01, 4.22
50–59	4,763/23,924	3.87	3.27, 4.58
60–69	4,316/20,154	4.36	3.68, 5.17
>=70	3,227/15,094	5.3	4.46, 6.30
**Sex**			
Male	11,080/58,340	1	Ref
Female	5,850/30,756	0.94	0.91, 0.98
**Ethnicity**			
White	11,588/65,011	1	Ref
Black, African, Caribbean or Black British	211/2,950	0.48	0.42, 0.55
South Asian	524/4,809	0.68	0.62, 0.75
Mixed or Multiple ethnic groups	51/649	0.57	0.43, 0.75
Other ethnic group	60/730	0.55	0.43, 0.71
Missing	4,496/14,948	1.95	1.88, 2.02
**Region**			
London	2,386/16,956	1	Ref
East Midlands	387/1,961	1.24	1.11, 1.38
East of England	612/2,930	1.26	1.15, 1.38
North East	843/3,876	1.2	1.10, 1.30
North West	4,203/20,015	1.21	1.15, 1.28
South East	2,912/15,164	1.15	1.08, 1.21
South West	2,052/10,930	1.03	0.97, 1.10
West Midlands	2,990/14,173	1.27	1.20, 1.34
Yorkshire And The Humber	536/3,041	1	0.91, 1.10
Missing	9/51	1.41	0.73, 2.71
**IMD quintile**			
1 Least deprived	2,320/12,367	1	Ref
2	2,703/14,508	1	0.94, 1.05
3	2,925/16,112	1.04	0.98, 1.10
4	3,764/20,319	1.12	1.06, 1.18
5 Most deprived	5,179/25,668	1.21	1.15, 1.27
Missing	39/123	2.05	1.50, 2.81

SFigure 11. Adjusted hazard ratios for ARLD-related mortality among patients with Possible ARLD by age, sex, ethnicity, region and IMD quintile

ARLD = alcohol related liver disease; IMD = Index of Multiple Deprivation; HR = hazard ratio; CI = confidence interval; Ref = reference. Missing categories were included in the regression analyses to minimise selection bias; however, HRs for missing categories should not be interpreted since many have small numbers and since it is not possible to know the characteristic of patients included in this group.

STable 17. Crude incidence rate ratios for hospitalisations among patients with ARLD by sociodemographic characteristics

	Definite ARLD			Probable ARLD			Possible ARLD		
	**Hospitalisations**	**Person years**	**Crude IRR (95% CI)**	**Hospitalisations**	**Person years**	**Crude IRR (95% CI)**	**Hospitalisations**	**Person years**	**Crude IRR (95% CI)**
**Age, years**									
18-29	2947	3233	1	3718	4031	1	11479	12503	1
30-39	19423	18914	1·04 (0·93, 1·17)	23181	22732	1·09 (0·98, 1·20)	32157	35555	1·08 (1·01, 1·15)
40-49	57170	53196	1·11 (1·00, 1·24)	73484	65274	1·20 (1·08, 1·32)	91837	84426	1·39 (1·31, 1·48)
50-59	76334	67734	1·13 (1·02, 1·27)	99342	83715	1·23 (1·12, 1·36)	128801	104801	1·56 (1·47, 1·65)
60-69	63282	49705	1·31 (1·17, 1·46)	86219	62790	1·47 (1·33, 1·62)	124807	83444	1·98 (1·86, 2·10)
≥70	29414	19416	1·48 (1·32, 1·65)	42968	26444	1·73 (1·56, 1·91)	85957	47131	2·38 (2·23, 2·53)
**Sex**									
Male	171584	149905	1	233391	189171	1	309727	240331	1
Female	76986	62289	1·05 (1·02, 1·08)	95521	75810	0·99 (0·96, 1·01)	165311	127525	0·96 (0·94, 0·99)
**Ethnicity**									
White	193940	168312	1	257061	210846	1	347703	274515	1
Black, African, Caribbean, or Black British	5306	3639	1·06 (0·95, 1·17)	6159	4571	0·93 (0·85, 1·01)	18513	13027	0·94 (0·88, 0·99)
South Asian	7514	6494	0·99 (0·92, 1·07)	10328	7907	1·07 (1·00, 1·14)	26139	20569	1·01 (0·97, 1·06)
Mixed or Multiple ethnic groups	729	916	0·70 (0·57, 0·86)	982	1163	0·68 (0·57, 0·81)	1964	2520	0·54 (0·47, 0·61)
Other ethnicity	970	1214	0·83 (0·69, 0·99)	1212	1501	0·75 (0·64, 0·87)	2809	2972	0·79 (0·70, 0·89)
Missing	40111	31623	1·25 (1·20, 1·29)	53170	38997	1·27 (1·23, 1·31)	77910	54256	1·27 (1·24, 1·31)

**Region**									
London	40959	36704	1	53369	45765	1	86299	71211	1
East Midlands	5577	4812	1·11 (1·01, 1·22)	6952	5757	1·13 (1·04, 1·23)	9339	7711	1·11 (1·03, 1·19)
East of England	7311	6408	1·15 (1·06, 1·25)	10088	8096	1·23 (1·14, 1·32)	15283	11712	1·21 (1·14, 1·29)
North East	13314	11657	1·12 (1·05, 1·20)	17353	14338	1·12 (1·05, 1·18)	20754	17086	1·09 (1·03, 1·15)
North West	63000	49751	1·23 (1·18, 1·29)	85638	63090	1·28 (1·23, 1·32)	113966	81945	1·27 (1·23, 1·31)
South East	37603	34118	1·08 (1·03, 1·13)	49873	42984	1·11 (1·07, 1·16)	75715	61235	1·14 (1·10, 1·18)
South West	32090	25603	1·23 (1·17, 1·29)	42502	32085	1·23 (1·18, 1·29)	63545	45227	1·24 (1·20, 1·29)
West Midlands	40020	35233	1·13 (1·08, 1·18)	51888	43140	1·14 (1·09, 1·18)	74584	58760	1·16 (1·12, 1·20)
Yorkshire And The Humber	8,633	7841	1.00 (0·92, 1·08)	11184	9649	1·06 (0·99, 1·13)	15465	12854	1·07 (1·01, 1·14)
Missing	63	70	1·23 (0·67, 2·28)	65	80	1·05 (0·59, 1·86)	88	119	0·98 (0·58, 1·65)
**IMD quintile**									
1 - Least deprived	30973	28128	1	42856	35787	1	67523	51981	1
2	37209	34818	0·96 (0·92, 1·01)	49882	43643	0·95 (0·91, 0·99)	75155	61307	0·95 (0·91, 0·98)
3	44467	38068	1·01 (0·97, 1·07)	57850	47322	0·97 (0·93, 1·02)	83044	66404	0·94 (0·90, 0·97)
4	55156	47815	1·03 (0·98, 1·08)	74361	59933	1·00 (0·96, 1·04)	108864	83453	0·96 (0·92, 0·99)
5 - Most deprived	80357	63075	1·14 (1·09, 1·19)	103494	77979	1·06 (1·02, 1·11)	139917	104341	1.00 (0·96, 1·03)
Missing	408	294	1·66 (1·20, 2·29)	469	321	1·45 (1·08, 1·95)	535	374	1·24 (0·93, 1·64)

Person years = total number of person years in the denominator population (per 100,000 person-years); IMD = Index of Multiple Deprivation; CI = confidence interval.

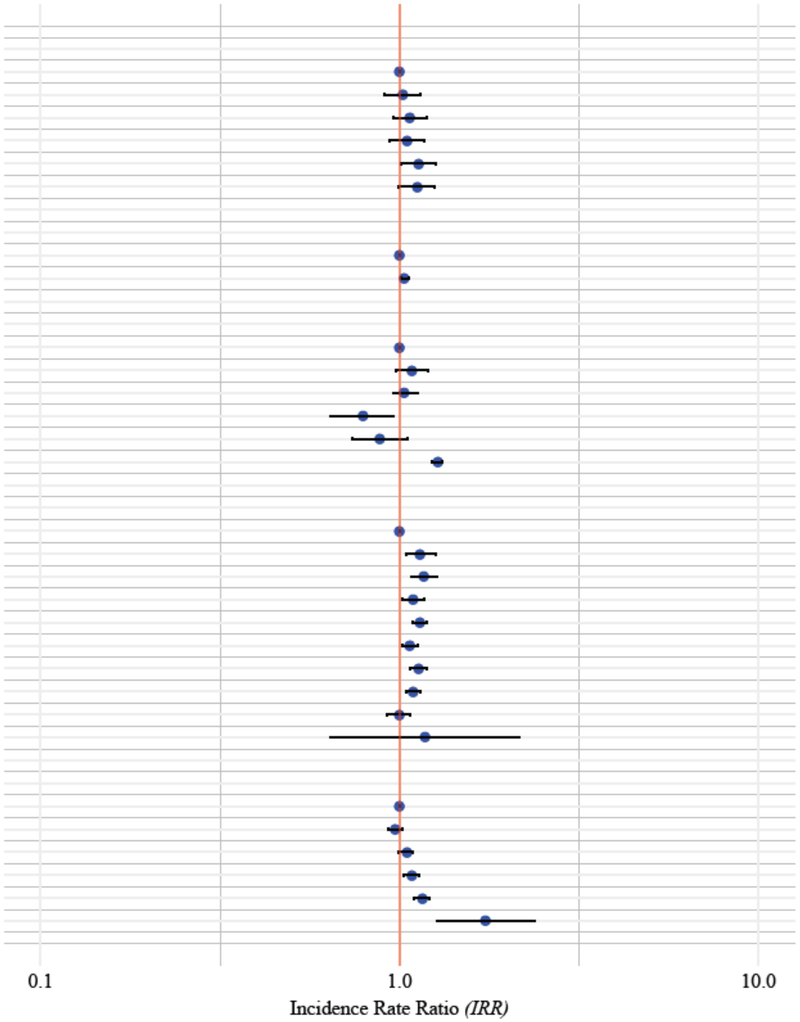

	Events/Patients	IRR	95% CI
**Age**			
18–29	2,947/835	1	Ref
30–39	19,423/4674	1.02	0.91–1.14
40–49	57,170/12,110	1.07	0.96–1.19
50–59	76,334/15,476	1.05	0.94–1.17
60–69	63,282/11,776	1.13	1.01–1.26
≥70	29,414/5,538	1.12	0.99–1.25
**Sex**			
Male	171,584/35,398	1	Ref
Female	76,986/15,010	1.03	1.01–1.06
**Ethnicity**			
White	193,940/38,814	1	Ref
Black, African, Caribbean or Black British	5,306/850	1.08	0.98–1.2
South Asian	7,514/1,587	1.03	0.96–1.12
Mixed or Multiple ethnic groups	729/224	0.79	0.64–0.96
Other ethnic group	970/303	0.88	0.74–1.05
Missing	40,111/8,631	1.28	1.23–1.32
**Region**			
London	40,959/8,713	1	Ref
East Midlands	5,577/1,139	1.14	1.04–1.26
East of England	7,311/1,564	1.17	1.08–1.27
North East	13,314/2,573	1.09	1.02–1.17
North West	63,000/11,956	1.14	1.09–1.19
South East	37,603/8,197	1.07	1.02–1.12
South West	32,090/6,137	1.13	1.07–1.19
West Midlands	40,020/8,330	1.09	1.04–1.14
Yorkshire And The Humber	8,633/1,771	1	0.92–1.07
Missing	63/29	1.18	0.64–2.16
**IMD**			
1 Least deprived	30,973/6,609	1	Ref
2	37,209/7,925	0.97	0.93–1.02
3	44,467/9,016	1.05	0.99–1.09
4	55,156/11,482	1.08	1.03–1.13
5 Most Deprived	80,357/15,291	1.16	1.1–1.21
Missing	408/86	1.74	1.27–2.38

SFigure 12. Adjusted incidence rate ratios for hospitalisations among patients with Definite ARLD by age, sex, ethnicity, region and IMD quintile

ARLD = alcohol related liver disease; IMD = Index of Multiple Deprivation; IRR = incidence rate ratio; CI = confidence interval; Ref = reference. Missing categories were included in the regression analyses to minimise selection bias; however, HRs for missing categories should not be interpreted since many have small numbers and since it is not possible to know the characteristic of patients included in this group.

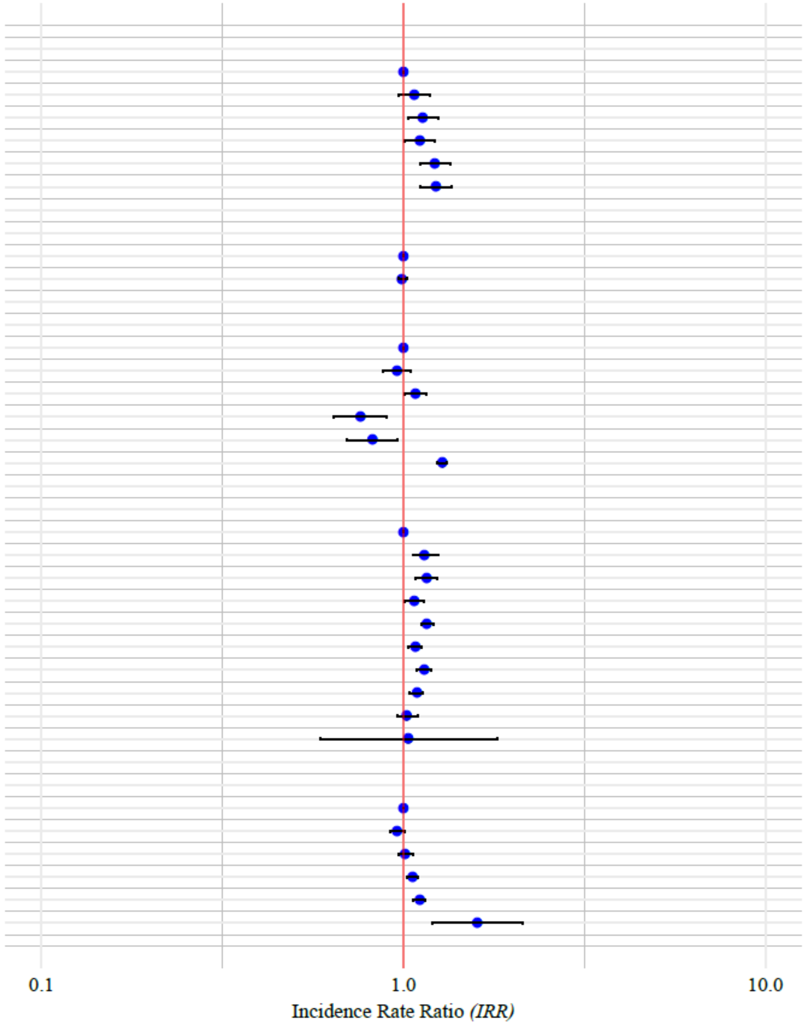

	Events/Patients	IRR	95% CI
**Age**			
18−29	3,718/1,045	1	Ref
30−39	23,181/5,684	1.07	0.97−1.18
40−49	73,484/14,875	1.13	1.03−1.25
50−59	99,342/19,467	1.11	1.01−1.22
60−69	86,219/15,411	1.22	1.11−1.35
≥70	42,968/8,199	1.23	1.11−1.36
**Sex**			
Male	233,391/45,962	1	Ref
Female	95,521/18,718	0.99	0.97−1.02
**Ethnicity**			
White	257,061/50,035	1	Ref
Black, African, Caribbean or Black British	6,159/1,102	0.96	0.88−1.05
South Asian	10,328/1,972	1.08	1.01−1.16
Mixed or Multiple ethnic groups	982/298	0.76	0.64−0.90
Other ethnic group	1,212/383	0.82	0.70−0.96
Missing	53,170/10,891	1.28	1.24−1.32
**Region**			
London	53,369/11,077	1	Ref
East Midlands	6,952/1,429	1.14	1.06−1.25
East of England	10,088/2,041	1.16	1.08−1.24
North East	17,353/3,295	1.07	1.01−1.14
North West	85,638/15,583	1.16	1.12−1.21
South East	49,873/10,659	1.08	1.03−1.12
South West	42,502/7,872	1.14	1.09−1.19
West Midlands	51,888/10,428	1.09	1.04−1.13
Yorkshire And The Humber	11,184/2,262	1.02	0.96−1.10
Missing	65/35	1.03	0.59−1.82
**IMD**			
1 Least deprived	42,856/8,677	1	Ref
2	49,882/10,290	0.96	0.92−1.01
3	57,850/11,502	1.01	0.97−1.06
4	74,361/14,757	1.06	1.02−1.10
5 Most Deprived	103,494/19,352	1.11	1.06−1.15
Missing	469/103	1.6	1.20−2.14

SFigure 13. Adjusted incidence rate ratios for hospitalisations among patients with Probable ARLD by age, sex, ethnicity, region and IMD quintile

ARLD = alcohol related liver disease; IMD = Index of Multiple Deprivation; IRR = incidence rate ratio; CI = confidence interval; Ref = reference. Missing categories were included in the regression analyses to minimise selection bias; however, HRs for missing categories should not be interpreted since many have small numbers and since it is not possible to know the characteristic of patients included in this group.

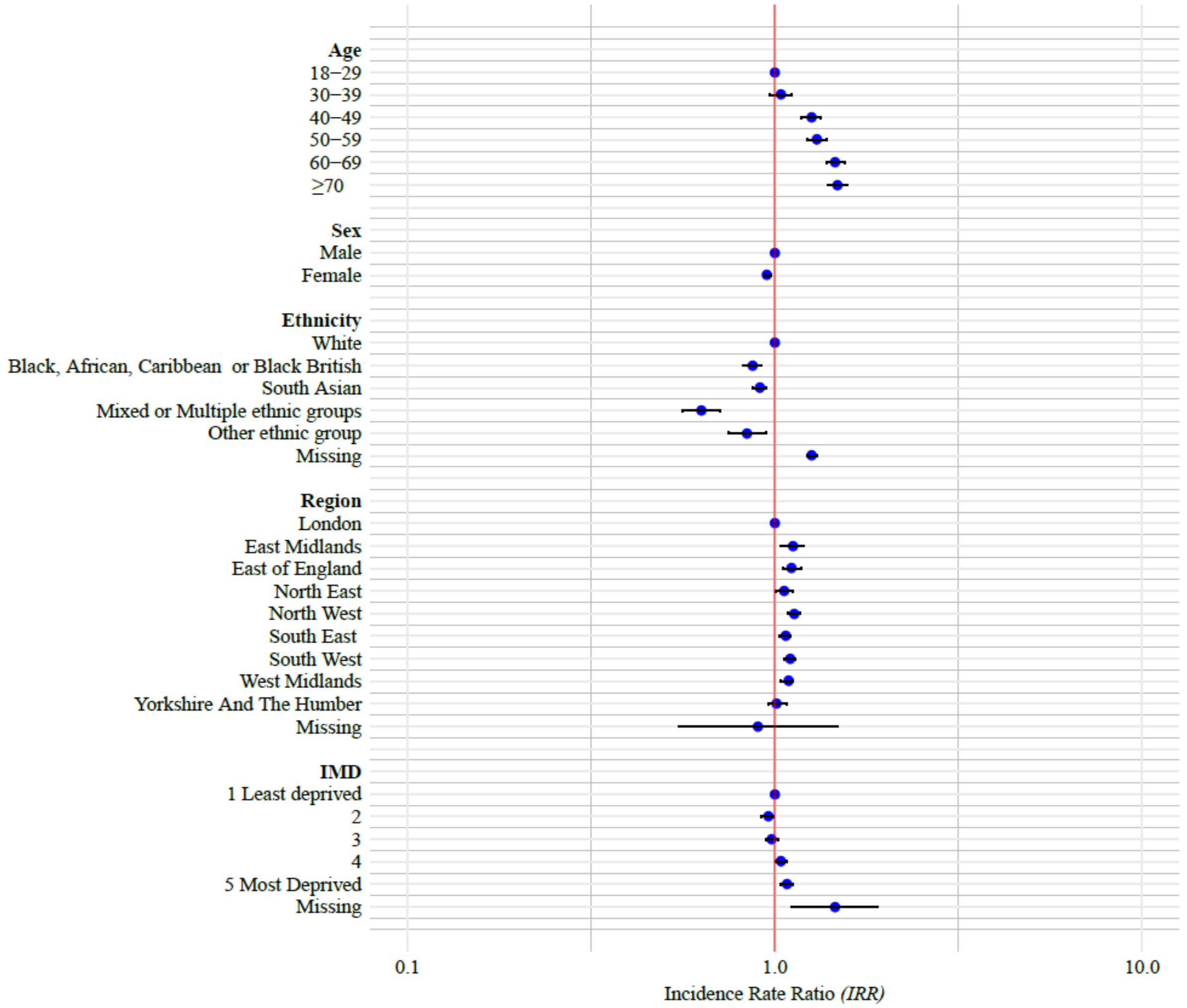

	Events/Patients	IRR	95% CI
**Age**			
18−29	11,479/3,020	1	Ref
30−39	32,157/8,447	1.04	0.97−1.11
40−49	91,837/18,458	1.26	1.18−1.33
50−59	128,801/23,924	1.3	1.23−1.38
60−69	124,807/20,154	1.46	1.38−1.55
≥70	85,957/15,094	1.48	1.39−1.58
**Sex**			
Male	309,727/58,340	1	Ref
Female	165,311/30,756	0.95	0.94−0.98
**Ethnicity**			
White	347,703/65,011	1	Ref
Black, African, Caribbean or Black British	18,513/2,950	0.87	0.82−0.92
South Asian	26,139/4,809	0.91	0.87−0.95
Mixed or Multiple ethnic groups	1,964/649	0.63	0.56−0.71
Other ethnic group	2,809/730	0.84	0.75−0.95
Missing	77,910/14,948	1.26	1.23−1.30
**Region**			
London	86,299/16,956	1	Ref
East Midlands	9,339/1,961	1.12	1.04−1.20
East of England	15,283/2,930	1.11	1.05−1.18
North East	20,754/3,876	1.06	1.01−1.12
North West	113,966/20,015	1.13	1.09−1.17
South East	75,715/15,164	1.07	1.03−1.10
South West	63,545/10,930	1.1	1.06−1.14
West Midlands	74,584/14,173	1.09	1.04−1.12
Yorkshire And The Humber	15,465/3,041	1.01	0.96−1.08
Missing	88/51	0.9	0.55−1.48
**IMD**			
1 Least deprived	67,523/12,367	1	Ref
2	75,155/14,508	0.96	0.92−0.99
3	83,044/16,112	0.98	0.95−1.02
4	108,864/20,319	1.04	1.01−1.08
5 Most Deprived	139,917/25,668	1.08	1.04−1.12
Missing	535/123	1.46	1.11−1.91

SFigure 14. Adjusted incidence rate ratios for hospitalisations among patients with Possible ARLD by age, sex, ethnicity, region and IMD quintile

ARLD = alcohol related liver disease; IMD = Index of Multiple Deprivation; IRR = incidence rate ratio; CI = confidence interval; Ref = reference. Missing categories were included in the regression analyses to minimise selection bias; however, HRs for missing categories should not be interpreted since many have small numbers and since it is not possible to know the characteristic of patients included in this group.

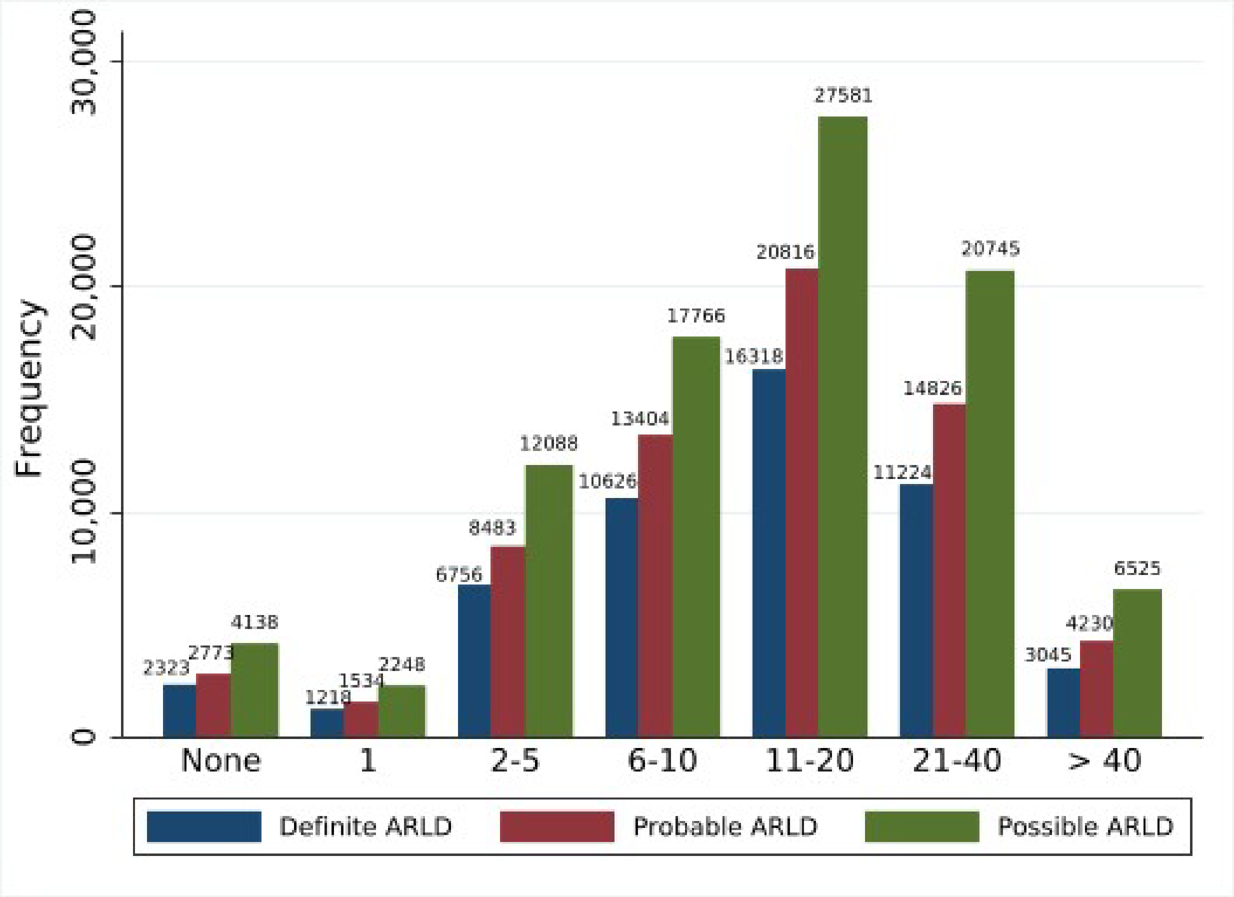

SFigure 15. Consultations in primary care within 2 years before diagnosis among patients with ARLD

ARLD = alcohol related liver disease

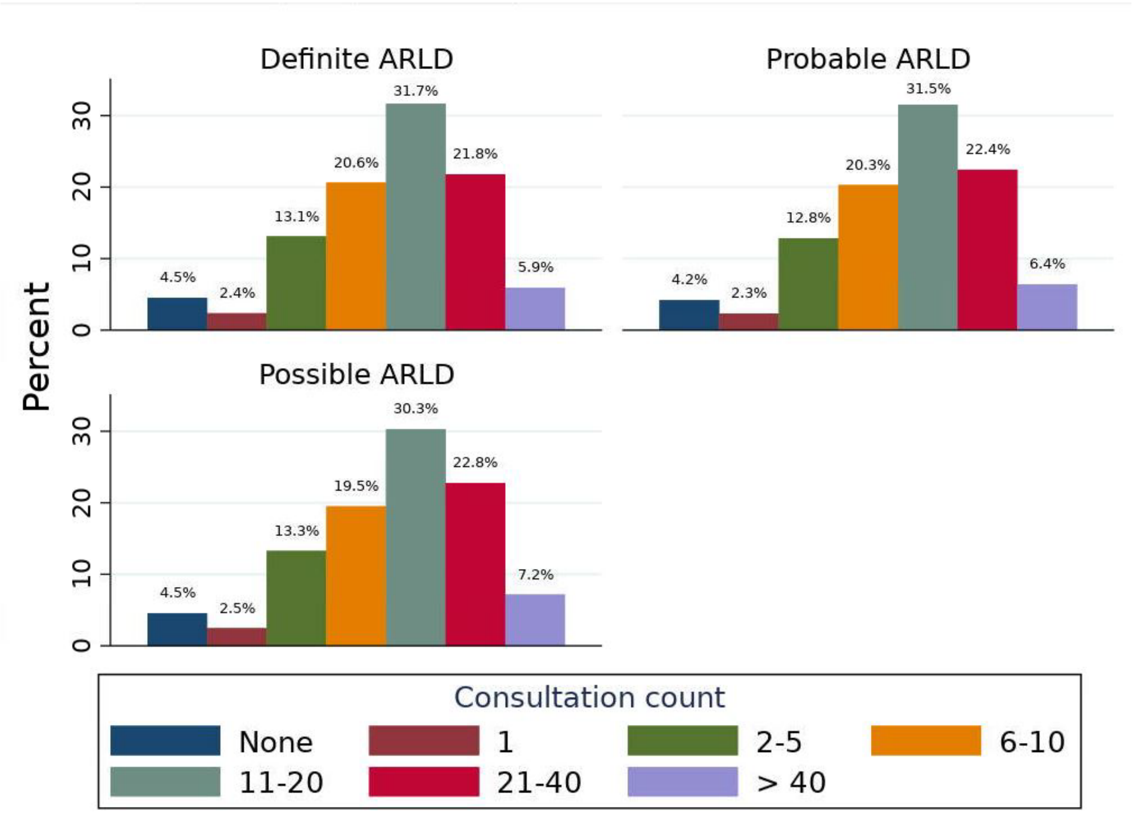

SFigure 16. Percentage of consultations in primary care within 2 years before diagnosis among patients with ARLD

ARLD = alcohol related liver disease

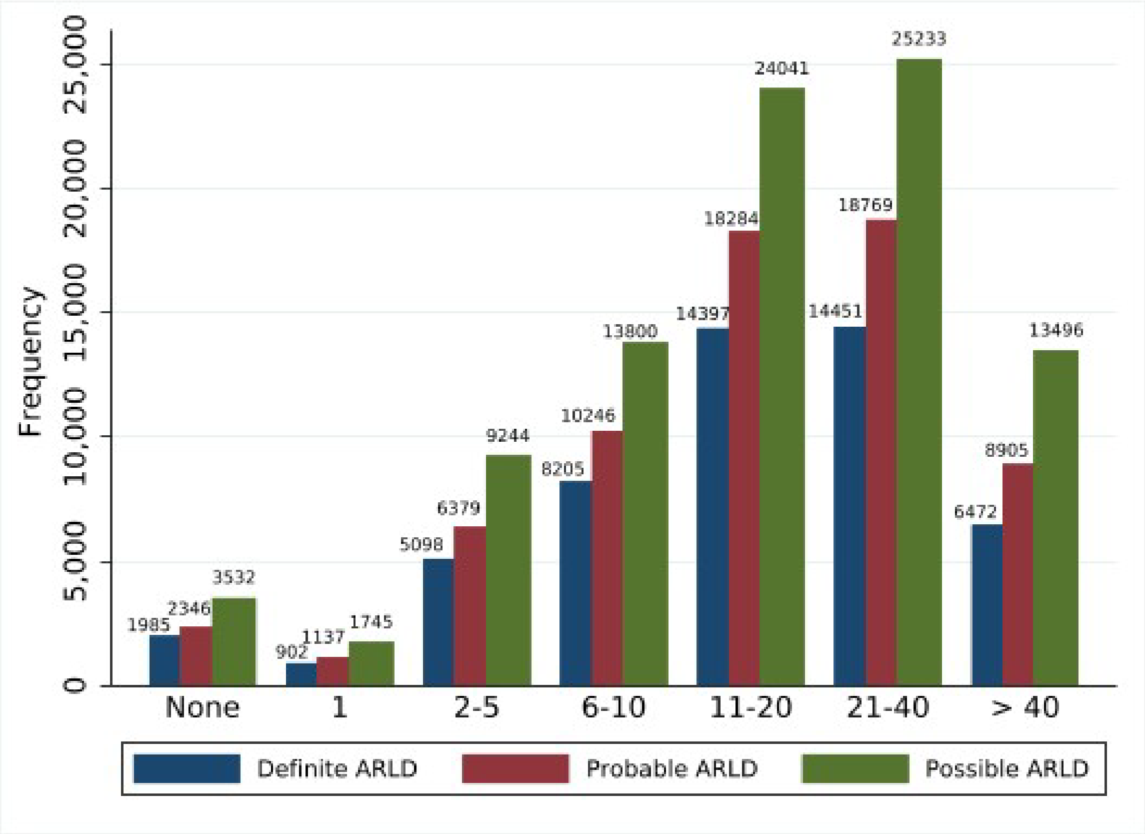

SFigure 17. Consultations in primary care within 3 years before diagnosis among patients with ARLD

ARLD = alcohol related liver disease

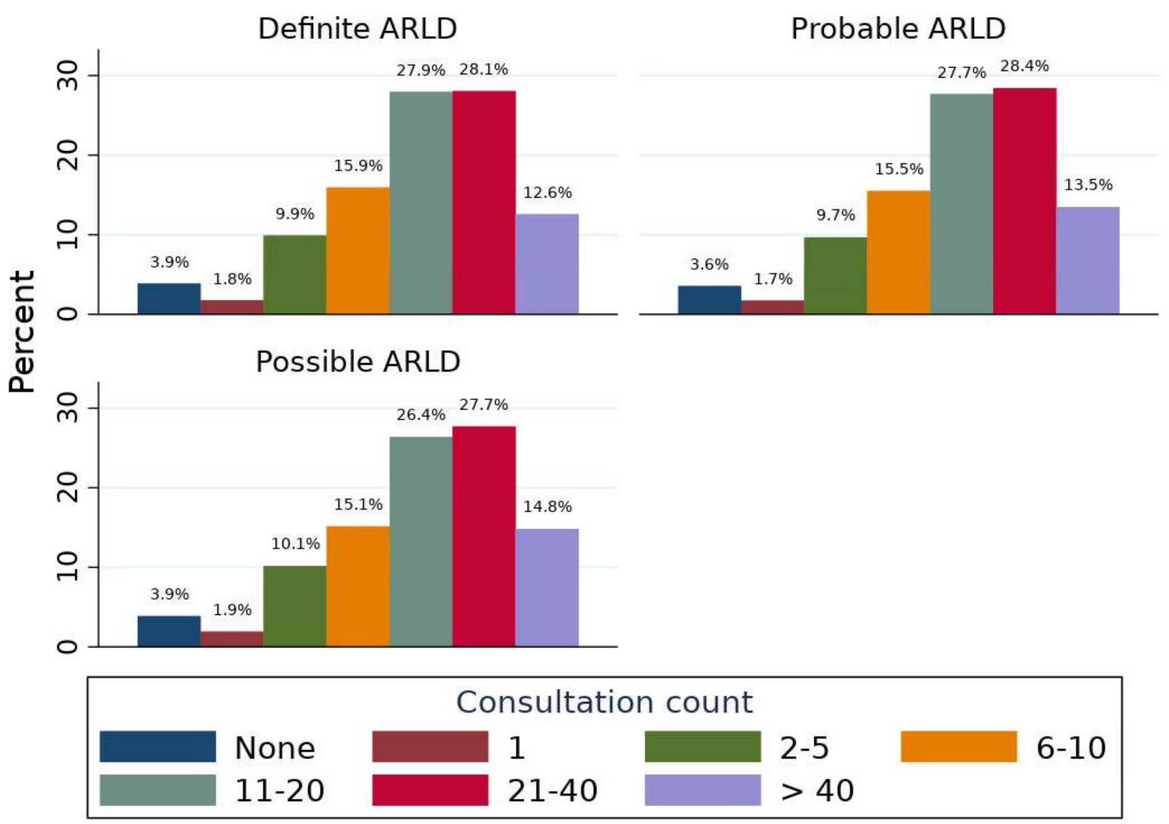

SFigure 18. Percentage of consultations in primary care within years before diagnosis among patients with ARLD
ARLD = alcohol related liver disease

---

[1] Wolf A, Dedman D, Campbell J, Booth H, Lunn D, Chapman J, et al. Data resource profile: Clinical Practice Research Datalink (CPRD) Aurum. Int J Epidemiol. 2019;48(6):1740-g.
[2] Clinical Practice Research Datalink. (2022). CPRD Aurum May 2022 (Version 2022.05.001) [Data set]. Clinical Practice Research Datalink. https://doi.org/10.48329/t89s-kf12
[3] Herrett E, Gallagher AM, Bhaskaran K, Forbes H, Mathur R, van Staa T, et al. Data Resource Profile: Clinical Practice Research Datalink (CPRD). Int J Epidemiol. 2015;44(3):827-36.
[4] Shiekh S.I., Harley M., Ghosh R.E. *et al.* Completeness, agreement, and representativeness of ethnicity recording in the United Kingdom's Clinical Practice Research Datalink (CPRD) and linked Hospital Episode Statistics (HES). *Popul Health Metrics* **21**, 3 (2023).
[5] Ministry of Housing, Communities and Local Government. The English Indices of Deprivation 2019 (IoD2019). 2019. Available at: https://assets.publishing.service.gov.uk/media/5d8e26f6ed915d5570c6cc55/IoD2019_Statistical_Release.pdf [accessed 12/02/2025]
